# Direct functionalization of white phosphorus with anionic dicarbenes and mesoionic carbenes: facile access to 1,2,3-triphosphol-2-ides†
†Electronic supplementary information (ESI) available. CCDC 1939608–1939615. For ESI and crystallographic data in CIF or other electronic format see DOI: 10.1039/c9sc04441h


**DOI:** 10.1039/c9sc04441h

**Published:** 2019-10-18

**Authors:** Dennis Rottschäfer, Sebastian Blomeyer, Beate Neumann, Hans-Georg Stammler, Rajendra S. Ghadwal

**Affiliations:** a Molecular Inorganic Chemistry and Catalysis , Inorganic and Structural Chemistry , Center for Molecular Materials , Faculty of Chemistry , Universität Bielefeld , Universitätsstr. 25 , Bielefeld , D-33615 , Germany . Email: rghadwal@uni-bielefeld.de ; https://www.ghadwalgroup.de

## Abstract

A series of unique C_2_P_3_-ring compounds [(ADC^Ar^)P_3_] (ADC^Ar^ = ArC{(DippN)C}_2_; Dipp = 2,6-iPr_2_C_6_H_3_; Ar = Ph **4a**, 3-MeC_6_H_4_**4b**, 4-MeC_6_H_4_**4c**, and 4-Me_2_NC_6_H_4_**4d**) are readily accessible in an almost quantitative yield by the direct functionalization of white phosphorus (P_4_) with appropriate anionic dicarbenes [Li(ADC^Ar^)]. The formation of 1,2,3-triphosphol-2-ides (**4a–4d**) suggests unprecedented [3 + 1] fragmentation of P_4_ into P_3_^+^ and P^–^. The P_3_^+^ cation is trapped by the (ADC^Ar^)^–^ to give **4**, while the putative P^–^ anion reacts with additional P_4_ to yield the Li_3_P_7_ species, a useful reagent in the synthesis of organophosphorus compounds. Remarkably, the P_4_ fragmentation is also viable with the related mesoionic carbenes (iMICs^Ar^) (iMIC^Ar^ = ArC{(DippN)_2_CCH}, i stands for imidazole-based) giving rise to **4**. DFT calculations reveal that both the C_3_N_2_ and C_2_P_3_-rings of **4** are 6π-electron aromatic systems. The natural bonding orbital (NBO) analyses indicate that compounds **4** are mesoionic species featuring a negatively polarized C_2_P_3_-ring. The HOMO–3 of **4** is mainly the lone-pair at the central phosphorus atom that undergoes σ-bond formation with a variety of metal-electrophiles to yield complexes [{(ADC^Ar^)P_3_}M(CO)_*n*_] (M = Fe, *n* = 4, Ar = Ph **5a** or 4-Me-C_6_H_4_**5b**; M = Mo, *n* = 5, Ar = Ph **6**; M = W, *n* = 5, Ar = 4-Me_2_NC_6_H_4_**7**).

## Abstract 2

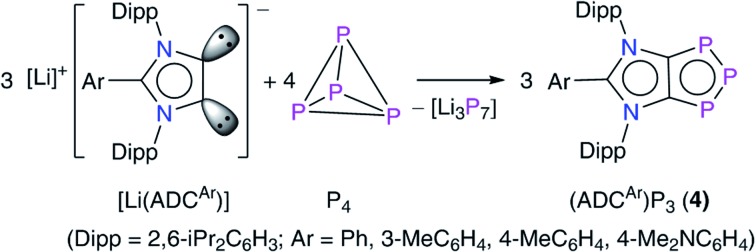
A series of unique C_2_P_3_-ring compounds [(ADC^Ar^)P_3_] (**4**) are readily accessible in an almost quantitative yield by the direct functionalization of white phosphorus (P_4_) with appropriate anionic dicarbenes [Li(ADC^Ar^)].

## Introduction

The direct conversion of white phosphorus (P_4_) into useful organophosphorus compounds (OPCs) is of significant interest because this excludes the involvement of corrosive Cl_2_ gas that is required to convert P_4_ into PCl_3_, a common starting material for OPCs, and thus minimizes the waste and energy consumption.^1^ The activation and subsequent functionalization of P_4_ has therefore become a topical objective.^2^ Both transition metal^3^ as well as main-group element^4^ compounds have been shown to activate or functionalize P_4_.^5^ In particular, compounds featuring a low-valent main-group element have made significant advances over the past years.^6^

**Fig. 1:**
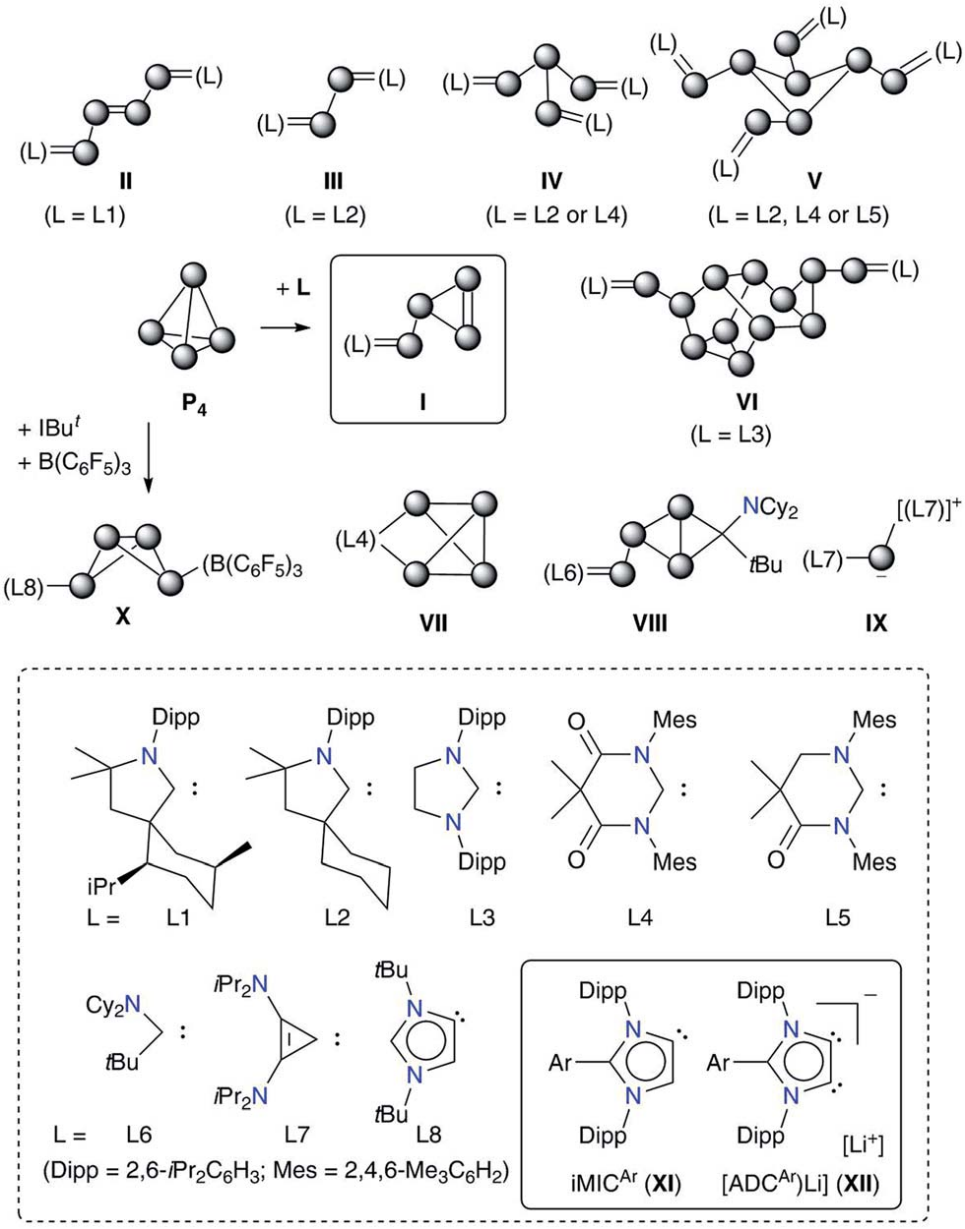
Singlet carbene-mediated P_4_ activation and fragmentation to **II–X** and a plausible intermediate **I**. Mesoionic carbenes (iMICs^Ar^, **XI**) and anionic dicarbenes (**XII**) ([Li^+^] = solvated lithium ion) investigated in the current study.

Among nonmetals, the use of singlet carbenes^7^ has given new impetus to the field of P_4_ activation as it leads to the direct C–P bond formation (Fig. 1).^8^ Several stable carbenes (L1–L7) undergo reactions with P_4_ and the fate of P_4_ fragmentation to give P_*n*_ (*n* = 1, 2, 4, 8 or 12) containing products **II–IX** depends on the relative σ-donor/π-acceptor (ambiphilic) property as well as the steric demand of carbenes.^7^ Weakly π-accepting NHCs such as IPr (IPr = C{(DippN)CH}_2_) do not react with P_4_, however, related derivatives containing the [P_2_] or [P_3_^–^] moiety are accessible by alternative methods.^9^ Sterically demanding 1,3-bis(*t*Bu)imidazol-2-ylidene (IBu^*t*^) activates P_4_ in combination with B(C_6_F_5_)_3_ to give **X**.6*h* This frustrated Lewis pair (FLP) type reactivity^10^ led to the transformation of the classical NHC (IBu^*t*^) into the mesoionic carbene (iMIC) L8 based on an 1,3-imidazole framework.

**Scheme 1:**
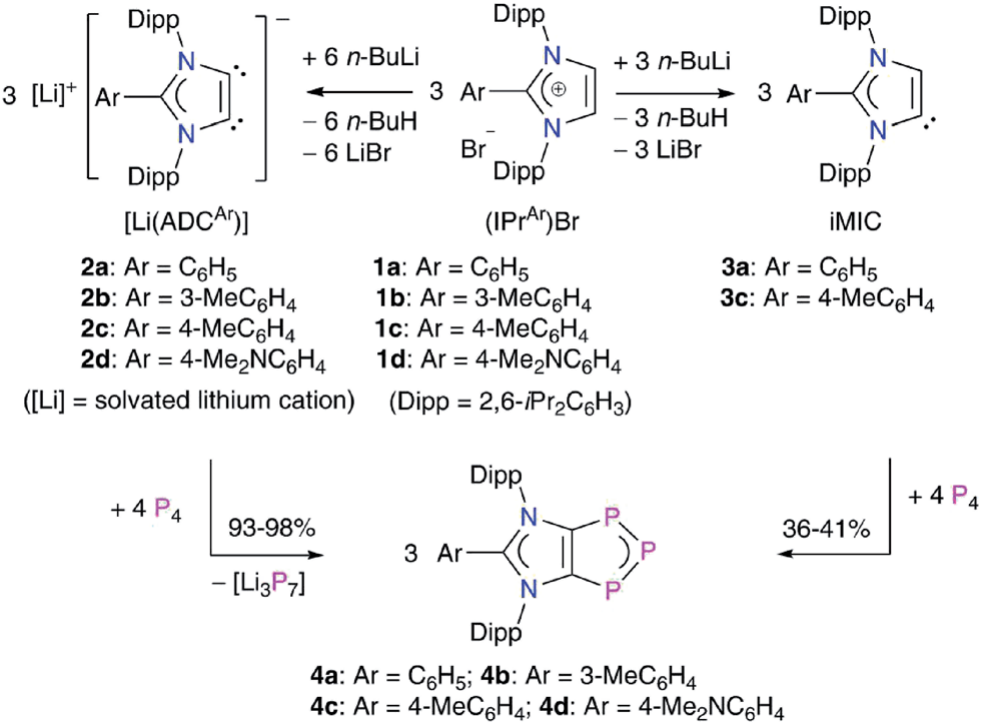
Synthesis of 1,2,3-triphosphol-2-ide derivatives **4a–4d** by the direct fragmentation of white phosphorus with [Li(ADC^Ar^)] (**2a–2d**). Reaction of iMICs^Ar^**3a** and **3c** with P_4_ to form **4a** and **4c**.

iMICs are very potent σ-donor ligands with almost negligible π-acceptor properties.^11^ Nonetheless, no reaction of an iMIC alone with P_4_ has been described so far. This is most likely due to their limited synthetic accessibility.11*a* We recently reported^12^ C5-protonated iMICs^Ar^ (**XI**) as well as C4/C5-ditopic anionic dicarbenes [Li(ADC^Ar^)] **XII** (Fig. 1) by the deprotonation of C2-arylated 1,3-imidazolium salts.^13^ The dicarbenes **XII** feature two adjacent C4/C5-nucleophilic sites, and thus are well endowed to affect unique dual P_4_ functionalization.5i,^14^ Herein, we showcase the direct functionalization of P_4_*via* unprecedented [3 + 1] fragmentation with [Li(ADC^Ar^] and iMICs^Ar^ to give the 1,2,3-triphosphol-2-ide derivatives [(ADC^Ar^)P_3_] (ADC^Ar^ = ArC{NDipp)C}_2_; Dipp = 2,6-iPr_2_C_6_H_3_; Ar = C_6_H_5_**4a**, 3-MeC_6_H_4_**4b**, 4-MeC_6_H_4_**4c**, and 4-Me_2_NC_6_H_4_**4d**) (Scheme 1).

## Results and discussion

Treatment of [Li(ADC^Ar^)] (**2a–2d**),^12^ which are readily accessible by the double deprotonation of C2-arylated 1,3-imidazolium salts **1a–1d** with *n*-BuLi, with P_4_ at room temperature afforded the 1,2,3-triphosphol-2-ides **4a–4d** as crystalline solids in almost quantitative yields (Scheme 1). Compounds **4a–4d** are indefinitely stable (as solids as well as in solutions) under an inert gas atmosphere. The formation of **4a–4d** indicates formal [3 + 1] fragmentation of P_4_ into P_3_^+^ and P^–^. The cationic P_3_^+^ species is captured by the ADCs to give **4a–4d**, whereas the P^–^ nucleophile reacts with additional P_4_ to eventually form the phosphide (P_7_)^3–^ anion, a very common species in metal mediated fragmentation of P_4_.^15^ Indeed, Li_3_P_7_ can be isolated as a red-brown solid,^15^,^16^ which was confirmed by its reaction with (IPr)HCl to give (IPr)PH, reported previously using Na_3_P_7_.^17^

Interestingly, treatment of iMICs^Ar^**3a** and **3c** with P_4_ also afforded, *albeit* in a lower yield, the corresponding products **4a** and **4c**, respectively. ^1^H NMR analyses of the crude reaction product indicate the presence of a 1 : 1 mixture of **4a** : **1a** and **4c** : **1c**, suggesting the reprotonation of iMICs^Ar^**3a** and **3b** to 1,3-imidazolium salts **1a** and **1c**. Pure **4a** and **4c** can be extracted from the mixture using toluene.

The ^1^H NMR spectra of **4a–4d** are very symmetric and show two doublets and one septet for the isopropyl groups along with the signals due to the aryl protons. The ^13^C{^1^H} NMR resonances for **4a–4d** are fully consistent with their ^1^H NMR spectra. The ^13^C{^1^H} NMR spectrum of **4a–4d** each exhibits a doublet at 167 ppm (*J*_P–C_ ≈ 84 Hz) for the backbone carbon atoms due to coupling with the ^31^P nucleus. The ^31^P{^1^H} NMR spectrum of **4a–4d** each shows a doublet at ∼73 ppm and a triplet at 325 ± 6 ppm in 2 : 1 ratio (*J*_P–P_ ≈ 500 Hz), indicating the presence of an AB_2_ type system with unsaturated P–P bonds.^18^

**Fig. 2:**
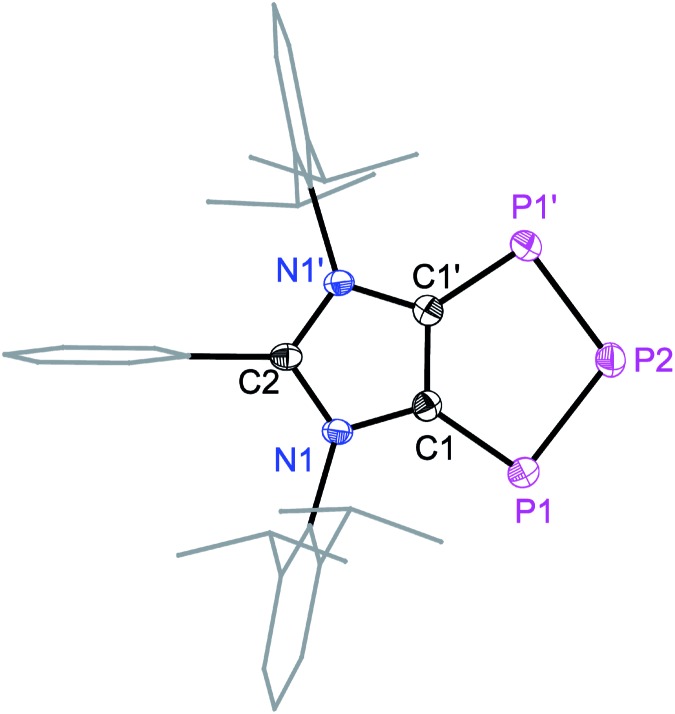
Solid-state molecular structure of **4a**. Hydrogen atoms are omitted for clarity. Symmetry code: 1 – *X*, +Y, 3/2 – *Z*. Selected experimental and calculated [M06-2X/def2SVP] bond lengths (Å) and angles (°): C1–C1′ 1.395(5) [1.402], N1–C1 1.404(3) [1.399], P1–C1 1.757(3) [1.764], P1–P2 2.103(1) [2.112], C1–P1–P2 94.9(1) [94.8], and P1–P2–P1′ 104.0(1) [104.1].

Solid-state molecular structures^19^ of **4a** (Fig. 2), **4b** (Fig. S47†), and **4c** (Fig. S48†) reveal the presence of a C_2_P_3_-ring that is coplanar with the imidazole C_3_N_2_-ring plane. The metrical parameters of **4a–4c** are comparable (Table S1†) and hence, for brevity, only **4a** is discussed here. The P1–P2 bond length of **4a** (2.103(1) Å) is intermediate of the sum of covalent radii for P=P double (2.04 Å) and P–P single (2.22 Å) bond lengths,^20^ indicating a partial π-bond character. Similarly, the C1–P1 (1.757(3) Å) bond length of **4a** is shorter compared to a classical C–P single bond length (1.85 Å)^15^ but compares well with C=P bond lengths (*ca.* 1.75 Å) of inversely polarized phosphaalkenes.^17^ The C1–C1′ (1.395(5) Å) and C2–N1 (1.404(3) Å) bond lengths of **4a** are elongated in comparison with those of **1a** (1.350(2) and 1.344(2) Å, respectively).^13^ The C1–C1′, C1/C2–P1 and P1–P2 bond lengths of **4a–4c** are comparable with the corresponding bond lengths of triphospholide anions [P_3_C_2_R_2_]^–^ (R = H, C–P 1.726(2) and 1.781(3), and P–P 2.081(1) and 2.094(1) Å; R = Ph, C–P 1.760(2) and 1.762(2), and P–P 2.091(2), 2.098(2) Å).^21^ Thus, **4a–4d** may be considered as the neutral analogues of the triphospholide anions.

**Scheme 2:**
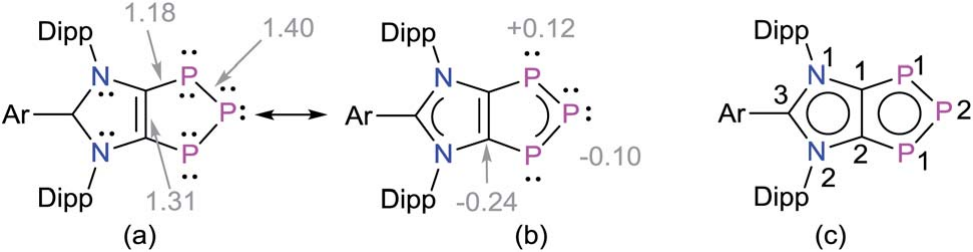
(a) Calculated Wiberg Bond Indices (WBIs) and (b) NPA atomic charges of 1,2,3-triphosphol-1,2-ides **4**. (c) Schematic representation of **4** with atom numberings.

To gain further insight into the electronic structures of **4a–4d**, we performed DFT calculations at the M06-2X/def2-TZVPP//M06-2X/def2-SVP level of theory. The computed NPA charges (Table S7†) at the P2 (–0.10*e*) and the C1/C2 (–0.24*e*) atoms are negative, whereas both the P1 atoms bear a positive charge (0.12*e*) (Scheme 2). The Wiberg Bond Indices (WBIs) of 1.40 (P–P), 1.18 (C–P), and 1.31 (C–C) indicate a partial double bond character. The WBI for the C1–C2 bond of **4a** (1.31) is significantly smaller compared to that of the imidazolium salt **1a** (WBI = 1.64). The WBIs for the C3–N1/2 bonds in **1** (1.28) and **4** (1.26) are, however, almost equal. Thus, compounds **4** may be described as mesoionic species with 6π-electron C_2_P_3_ and C_3_N_2_ aromatic systems (Scheme 2c). The nitrogen atoms contribute 4π-electrons to the C_3_N_3_-ring, whereas the P_3_ unit shares 4π-electrons with the C_2_P_3_-ring. The 2π-electrons of the C1=C2 bond are pooled by both the ring systems. Indeed, calculated nucleus-independent chemical shift (NICS)^22^ values for **4a–4d** (Table 1) suggest the aromaticity of the C_3_N_2_- and C_2_P_3_-rings. For comparison, we also calculated the NICS values for C_6_H_6_ and cyclobutadiene (CBD) molecules.

**Table 1:** Calculated NICS values for the C_3_N_2_/C_2_P_3_ units of **4a–4d** at the M06-2X/def2TZVPP//M06-2X/def2SVP level of theory

C_3_N_2_/C_2_P_3_	**4a**	**4b**	**4c**	**4d**	**5a**	C_6_H_6_/CBD^*a*^
NICS(0)	–7.08/–10.19	–7.29/–10.31	–7.29/–10.31	–6.77/–10.37	–7.57/–9.95	–7.53/33.21
NICS(1)	–5.94/–10.18	–6.11/–10.28	–6.11/–10.23	–5.64/–10.21	–6.29/–9.58	–10.19/21.09
NICS(2)	–2.43/–5.51	–2.53/–5.21	–2.53/–5.53	–2.36/–5.52	–2.52/–5.12	–5.22/4.98

^*a*^CBD (cyclobutadiene).

**Fig. 3:**
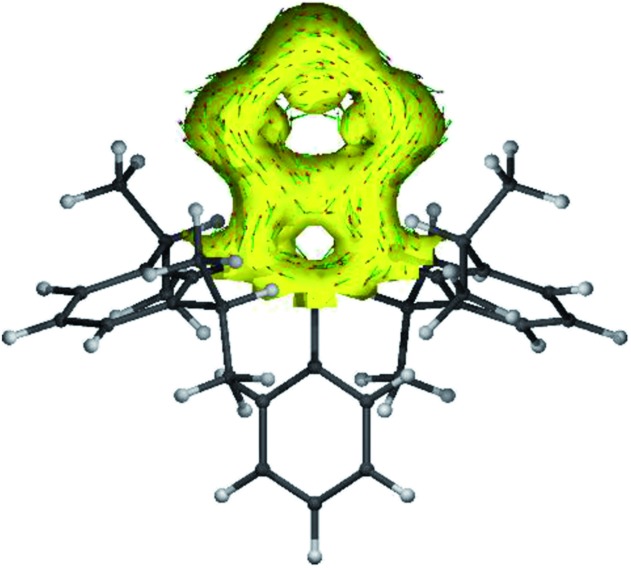
AICD plot (based on M06-2X/def2-TZVPP//def2-SVP calculations) of the C_3_N_2_P_3_ core of compound **4a**. The isovalue was arbitrarily chosen to be 0.03, the magnetic field is orthogonal to the C_2_P_3_-plane and points towards the viewer, and thus clockwise ring currents represent aromatic systems, whereas counter-clockwise ring currents are indicative of antiaromatic systems. AICD plots of the complete molecules **4a–4d** are given in the ESI.†

**Fig. 4:**
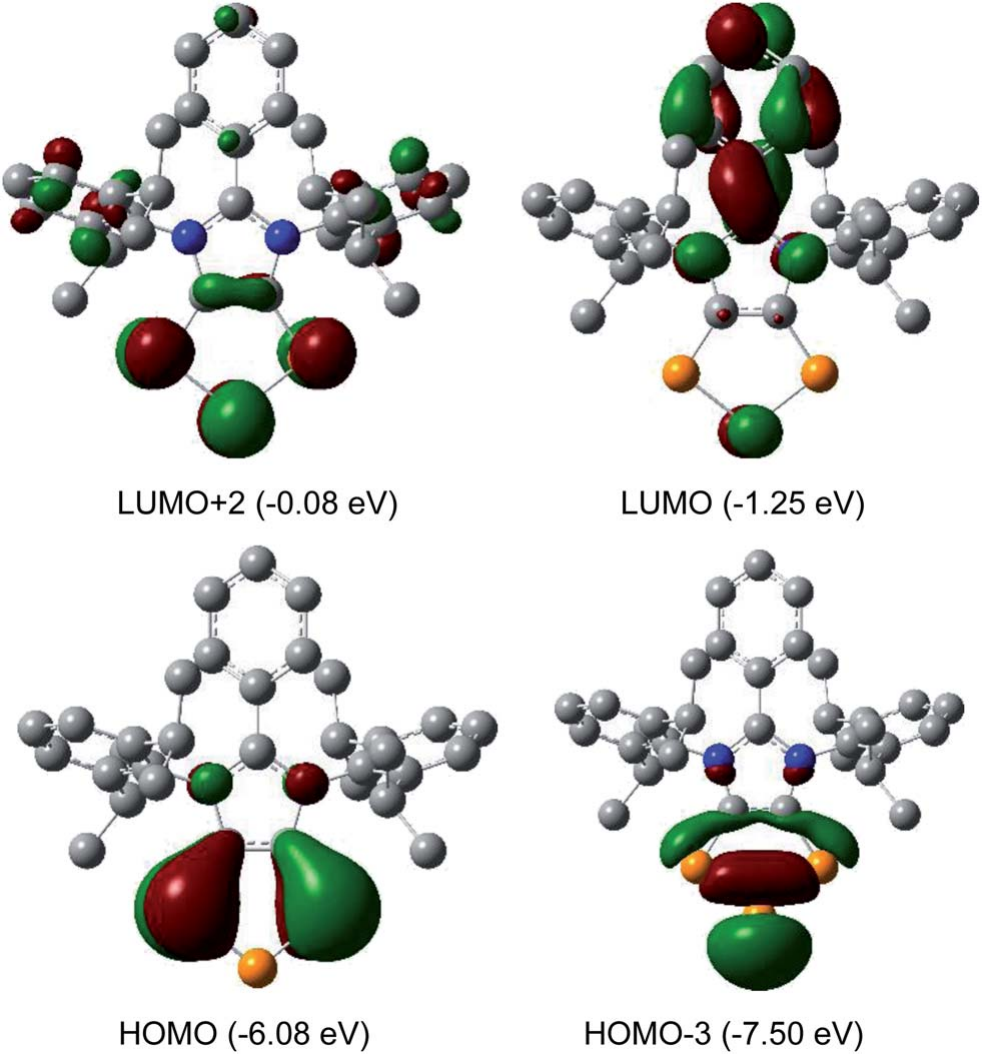
Selected MOs of **4a** calculated at the M06-2X/def2-TZVPP//def2-SVP level of theory with an isovalue of 0.04. Hydrogen atoms were omitted for clarity.

The anisotropy of current-induced density (AICD) has been used to study the aromatic behavior of several molecules.^23^ The AICD plots of **4a** (Fig. 3) and **4b–4d** (Fig. S62†) clearly show significant delocalization of the π-electrons of both the C_3_N_2_ and the C_2_P_3_ heterocycles, forming one coherent π-system.

The HOMO of compounds **4a** (Fig. 4) and **4b–4c** (Fig. S58–S60†) corresponds to the π-orbitals of the C–P bonds with a small contribution from the lone pairs at the nitrogen atoms. The HOMO–1 corresponds mainly to the π-orbitals of the P_3_ and the C_2_ moieties of the C_2_P_3_-ring. Like in alkali metal 1,2,3-triphospholides,21*b* the analyses of frontier molecular orbitals, HOMO and HOMO–1 in particular, of **4a–4d** reveal the mixing of phosphorus orbitals with lone-pair character amongst the π-manifold frontier orbitals. The HOMO–3 and HOMO–2 are the lone pairs on the central and neighbouring P atoms, respectively. The LUMO of **4a–4d** corresponds to the π* orbital of the aryl group on the C3 carbon atom along with a p-orbital at the central phosphorus atom. The LUMO+2 corresponds mainly to the π*-orbitals of the C_2_P_3_ unit.

**Scheme 3:**
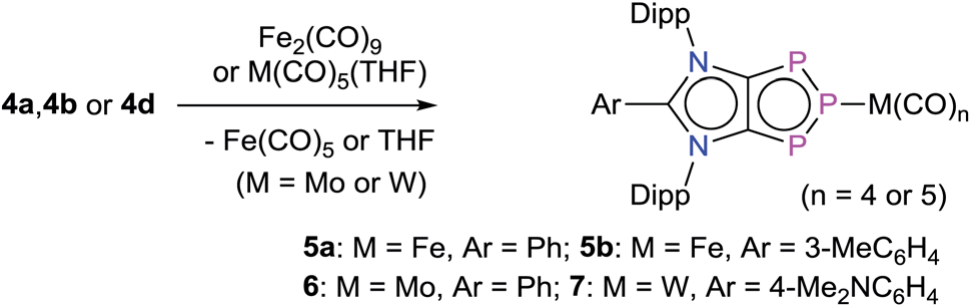
Synthesis of complexes [{(ADC^Ar^)P_3_}M(CO)_*n*_] **5a**, **5b**, **6**, and **7**.

The intriguing electronic structures of **4** prompted us to investigate their ligand properties as they may function as neutral two electron σ-donors (*via* phosphorus atoms) and/or 6π-electron η^5^-donors (C_2_P_3_-ring) like triphospholide^21^ and cyclopentadienyl anions. Treatment of **4a**, **4b**, and **4c** with Fe_2_(CO)_9_ or M(CO)_5_(THF) (M = Mo or W) led to the formation of related complexes **5a**, **5b**, **6**, and **7** (Scheme 3). In all complexes, the central phosphorus atom functions as a two-electron σ-donor ligand to bind to the M(CO)_*n*_ moiety. This is consistent with the NBO analysis, which suggests higher charge accumulation at the central phosphorus atom with respect to that of the neighbouring phosphorus atoms. The ^31^P{^1^H} NMR spectrum of **5a**, **5b**, **6**, and **7** each exhibits one doublet (**5a**: 145; **5b**: 145; **6**: 160; **7**: 157 ppm) and one triplet (**5a**: 316; **5b**: 315; **6**: 299; **7**: 250 ppm), which have been upfield shifted with respect to that of **4a** (173, 332 ppm), **4b** (173, 331 ppm), and **4d** (173, 319 ppm). In the ^31^P{^1^H} NMR spectrum of **7**, the triplet at 250 ppm is accompanied by the ^183^W satellites (*J*_P–W_ = 202 Hz).

**Fig. 5:**
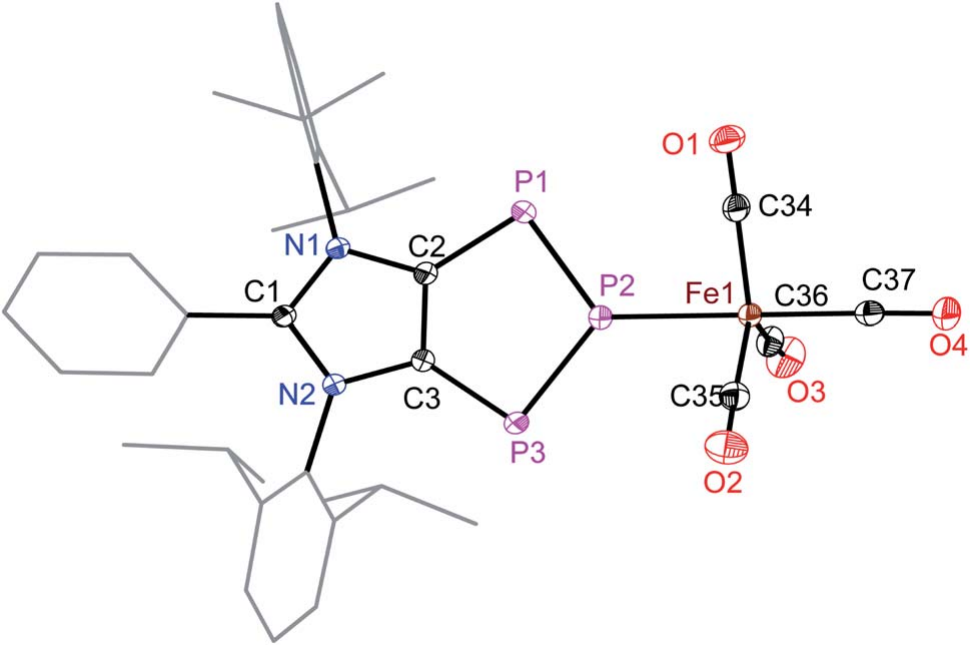
Solid-state molecular structure of **5a**. Hydrogen atoms and one solvent toluene molecule are omitted for clarity. Selected bond lengths (Å) and angles (°): C2–C3 1.394(2), C2–N1 1.399(1), C3–N2 1.403(1), C2–P1 1.756(1), C3–P3 1.764(1), P1–P2 2.081(1), P2–P3 2.089(1), P2–Fe1 2.240(1), Fe1–C34 1.791(1), Fe1–C35 1.797(2), Fe1–C36 1.810(1), Fe1–C37 1.783(1), P1–P2–P3 109.1(2), and P2–Fe1–C37 178.5(1).

The iron atom in **5a** (Fig. 5) and **5b** (Fig. S49†) each features a trigonal-bipyramidal geometry. Three equatorial positions are occupied by CO ligands, whereas one CO and one **4a** or **4b** are present at the axial positions. The P–Fe bond length of **5a** (2.240(1) Å) compares well with that of triphosphaindane-derived P_3_Fe_3_ iron-carbonyl clusters (av. 2.244 Å).^24^ Interestingly, the metrical parameters of the C_3_N_2_- and C_2_P_3_-rings of **5a** and **5b** are very similar to those of the precursors **4a** and **4b**, respectively. This indicates that the aromatic π-systems remain virtually intact upon complexation of **4a** and **4b** with the Fe(CO)_4_ fragment. As expected, the molecular structures of **6** (Fig. S50†) and **7** (Fig. S51†) feature six-fold coordinated Mo and W atoms, respectively.

**Fig. 6:**
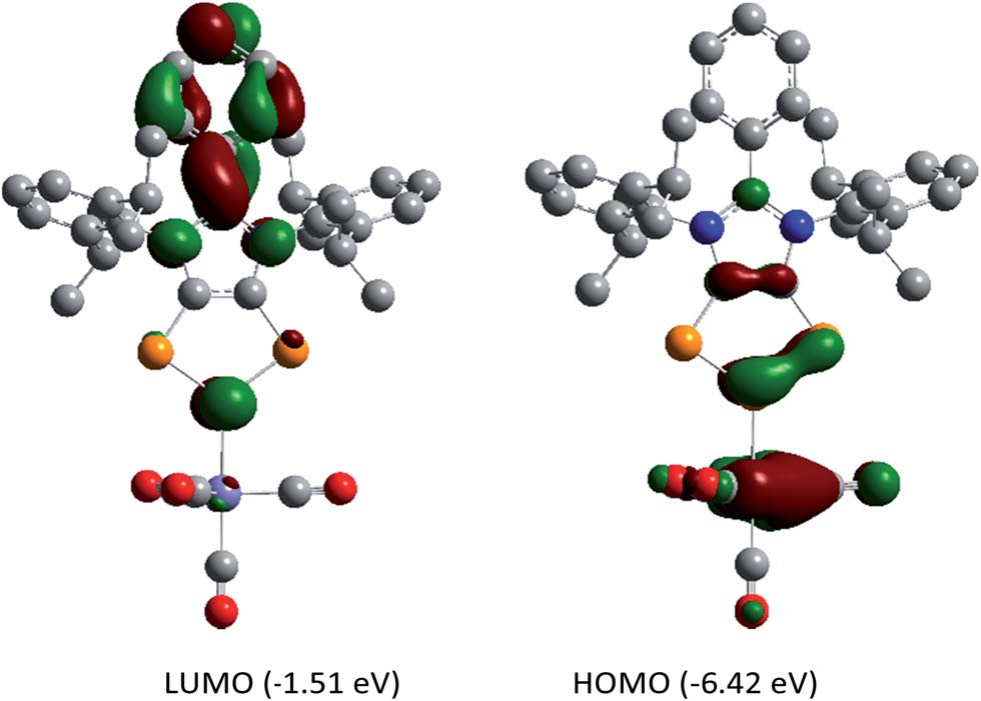
Frontier molecular orbitals of **5a** calculated at the M06-2X/def2-TZVPP//def2-SVP level of theory. The isovalue was arbitrarily chosen to be 0.04. Hydrogen atoms were omitted for clarity.

DFT calculations suggest that the HOMO of **5a** (Fig. 6) is mainly located at the iron atom and has some contribution from the π-orbitals of the C–C and one P–P bond. The LUMO is comparable to that of **4a** but is lower in energy by –0.26 eV, indicating metal-to-ligand π-back bonding. The aromaticity of the C_2_P_3_ moiety in **5a** remains almost unchanged as indicated by NICS(0) = –9.95, NICS(1) = –9.58, and NICS(2) = –5.12 values. The aromaticity of **5a** is also corroborated by the AICD plot (Fig. S62†).

## Experimental

All syntheses and manipulations were carried out under an inert gas atmosphere (Ar or N_2_) using standard *Schlenk* techniques or a glove box (MBraun LABMasterPro). Solvents were dried over appropriate drying agents, distilled, and stored over a 3 Å molecular sieve prior to use. Deuterated solvents were dried over appropriate drying agents, distilled, and stored inside a glove box. NMR spectra were recorded on a Bruker Avance III 500 or a Bruker Avance III 500 HD spectrometer. Chemical shifts (in *δ*, ppm) are referenced to the solvent residual signals of CD_2_Cl_2_: ^1^H 5.32; ^13^C 53.84 and C_6_D_6_: ^1^H 7.16; ^13^C 128.62 ppm. ESI mass spectra were recorded using an Esquire 3000 ion trap mass spectrometer (Bruker Daltonik GmbH, Bremen, Germany) equipped with a nano-ESI source. Samples were dissolved in CH_2_Cl_2_ and introduced by static nano-ESI using in-house pulled glass emitters. Nitrogen served as a nebulizer gas as well as a dry gas and was generated by a Bruker nitrogen generator NGM 11. Helium served as a cooling gas for the ion trap. The mass axis was externally calibrated with ESI-L Tuning Mix (Agilent Technologies, Santa Clara, CA, USA) as the calibration standard. UV/vis spectra were recorded on a ThermoFisher Evolution 300 spectrophotometer. Infrared spectra were recorded using a Bruker Alpha-T FTIR spectrometer equipped with a Bruker Platinum diamond ATR unit. Melting points were measured with a Büchi B-545 melting point apparatus. (IPr^Ar^)Br salts **1a–1d** (Ar = Ph, 3-MeC_6_H_4_, 4-MeC_6_H_4_ or 4-Me_2_NC_6_H_4_) were synthesized following the reported method.13*a**n*-BuLi (2.5 M solution in hexanes, Sigma-Aldrich) was used as received. White phosphorus was sublimed and stored inside a glovebox. Commercially available Fe_2_(CO)_9_ (Sigma-Aldrich), Mo(CO)_6_ (Fluorochem), and W(CO)_6_ (Sigma-Aldrich) were used as supplied.

### Synthesis of compound (ADC^Ph^)P_3_ (**4a**)

To a 15 mL THF suspension of **1a** (0.88 g, 1.6 mmol), *n*-BuLi (2.5 M, 1.4 mL, 3.5 mmol) was added at –40 °C. The resulting reaction mixture was stirred at –20 °C for 1 h and then at room temperature (25 °C) for 15 minutes to obtain a clear light brown solution of **2a**.^12^ To this solution, solid P_4_ (0.4 g, 3.2 mmol) was added in one portion and then stirred overnight at rt. The resulting dark suspension was refluxed for 2 h and the red insoluble material (probably a mixture of Li_3_P_7_ and other polyphosphides) was removed by filtration. The volatiles from the filtrate were removed under vacuum to give a brown residue, which was extracted with dichloromethane, dried under vacuum, washed with toluene (2 × 10 mL), and re-dried to obtain compound **4a** as a yellow solid. Yield: 96% (0.86 g). Single crystals suitable for X-ray diffraction analysis were grown by storing a saturated toluene solution of **4a** at –24 °C for three days. Mp: 343 °C. Elem. anal. (%), calcd for C_33_H_39_N_2_P_3_ (556.6): C, 71.21; H, 7.06; N, 5.03; found: C, 71.02; H, 6.84; N, 4.87. ^1^H NMR (500 MHz, CD_2_Cl_2_, 298 K): *δ* = 7.59 (t, *J* = 7.7 Hz, 2H, *p*-C_6_*H*_3_), 7.38 (d, *J* = 7.8 Hz, 4H, *m*-C_6_*H*_3_), 7.34 (t, *J* = 6.6 Hz, 1H, *p*-C_6_*H*_5_), 7.23–7.18 (m, 4H, *o*-, *m*-C_6_*H*_5_), 2.62 (sept, *J* = 6.6 Hz, 4H, C*H*(CH_3_)_2_), 1.26 (d, *J* = 6.6 Hz, 12H, CH(C*H*_3_)_2_), and 1.03 (d, *J* = 6.7 Hz, 12H, CH(C*H*_3_)_2_) ppm. ^13^C{^1^H} NMR (126 MHz, CD_2_Cl_2_, 298 K): *δ* = 167.6 (d, *J*_P–C_ = 84.4 Hz, *C*P); 149.2 (N*C*N); 146.2, 133.5, 132.0, 131.8, 129.8, 129.1, 125.8, and 123.8 (*C*_6_H_3_, *C*_6_H_5_); 29.7 (*C*H(CH_3_)_2_); 26.1 and 23.5 (CH(*C*H_3_)_2_) ppm. ^31^P{^1^H} NMR (202 MHz, CD_2_Cl_2_, 298 K): *δ* = 332.3 (t, *J*_P–P_ = 506 Hz) and 173.7 (d, *J*_P–P_ = 506 Hz) ppm. MS (ESI, positive mode): *m*/*z* = 557.3 [**4a** + H]^+^. UV-vis (*λ*/nm *ε* (M^–1^ cm^–1^)): 282 (22336), 346 (31017), and 361 (31397).

Compounds **4b–4d** were prepared by employing a similar protocol to that described for **4a** using the appropriate precursor **1b**, **1c** or **1d**, *n*-BuLi, and P_4_.

### (ADC^3-Tol^)P_3_ (**4b**)

Yield: 98% (0.90 g). Mp: 338–341 °C. Elem. anal. (%), calcd for **4b**, C_34_H_41_N_2_P_3_, (570.6): C, 71.56; H, 7.24; N 4.91; found C, 70.64; H, 7.33; N 4.68. ^1^H NMR (500 MHz, CD_2_Cl_2_, 298 K): *δ* = 7.57 (t, *J* = 7.8 Hz, 2H, *p*-C_6_*H*_3_), 7.35 (d, *J* = 7.8 Hz, 4H, *m*-C_6_H_3_), 7.14 (d, *J* = 7.6 Hz, 1H, *o*-C_6_*H*_4_), 7.08 (t, *J* = 7.8 Hz, 1H, *m*-C_6_*H*_4_), 7.02 (s, 1H, *o*-C_6_*H*_4_), 6.96 (d, *J* = 7.8 Hz, 1H, *p*-C_6_*H*_4_), 2.60 (sept, *J* = 6.7 Hz, 4H, C*H*(CH_3_)_2_), 2.10 (s, 3H, C*H*_3_), 1.24 (d, *J* = 6.7 Hz, 12H, CH(C*H*_3_)_2_), and 1.03 (d, *J* = 6.8 Hz, 12H, CH(C*H*_3_)_2_) ppm. ^13^C{^1^H} NMR (126 MHz, CD_2_Cl_2_, 298 K): *δ* = 167.5 (d, *J*_P–C_ = 84.5 Hz, *C*P); 149.6 (N*C*N); 146.3, 139.3, and 133.6 (i-*C*_6_H_4_ and i-, *m-C*_6_H_3_); 132.7 (*o-C*_6_H_4_); 131.7 (*p-C*_6_H_3_); 130.5 (*o-C*_6_H_4_); 129.5 and 128.9 (*m-C*_6_H_4_); 126.9, 125.8, and 123.71 (*m-C*_6_H_3_); 29.7 (*C*(CH_3_)_2_); 26.2 and 23.5 (C(*C*H_3_)_2_); 21.2 (*C*H_3_) ppm. ^31^P{^1^H} NMR (202 MHz, CD_2_Cl_2_, 298 K): *δ* = 331.1 (t, *J*_P–P_ = 506 Hz) and 173.6 (d, *J*_P–P_ = 506 Hz) ppm. MS (ESI, positive mode): *m*/*z* = 571.3 [**4b** + H]^+^. UV-vis (*λ*/nm *ε* (M^–1^ cm^–1^)): 280 (25637), 345 (36323), and 361 (36539).

### (ADC^4-Tol^)P_3_ (**4c**)

Yield: 93% (0.85 g). Single crystals suitable for X-ray diffraction were obtained by storing a saturated toluene solution of **4c** for three days at –24 °C. Mp: 339–343 °C. Elem. anal. (%), calcd for C_34_H_41_N_2_P_3_ (570.6): C, 71.56; H, 7.24; N, 4.91; found C, 71.11; H, 7.06; N, 4.65. ^1^H NMR (500 MHz, CD_2_Cl_2_, 298 K): *δ* = 7.59 (t, *J* = 7.8 Hz, 2H, *p*-C_6_*H*_3_), 7.38 (d, *J* = 7.8 Hz, 4H, *m*-C_6_*H*_3_), 7.05 (d, *J* = 8.4 Hz, 2H, C_6_*H*_4_), 7.02 (d, *J* = 8.3 Hz, 2H, C_6_*H*_4_), 2.61 (sept, *J* = 6.8 Hz, 4H, C*H*(CH_3_)_2_), 2.23 (s, 3H, C*H*_3_), 1.26 (d, *J* = 6.7 Hz, 12H, CH(C*H*_3_)_2_), and 1.03 (d, *J* = 6.9 Hz, 12H, CH(C*H*_3_)_2_) ppm. ^13^C{^1^H} NMR (126 MHz, CD_2_Cl_2_, 298 K): *δ* = 167.5 (d, *J*_P–C_ = 84.1 Hz, *C*P); 146.2 (N*C*N); 143.1, 133.7, 131.7, 129.8, 129.7, 125.8, and 120.9 (*C*_6_H_3_ and *C*_6_H_4_); 29.7 (*C*H(CH_3_)_2_); 26.1 and 23.5 (CH(*C*H_3_)_2_); 21.7 (*C*H_3_) ppm. ^31^P{^1^H} NMR (202 MHz, CD_2_Cl_2_, 298 K): *δ* = 329.9 (t, *J*_P–P_ = 506 Hz) and 173.6 (d, *J*_P–P_ = 506 Hz) ppm. MS (ESI, positive mode): *m*/*z* = 571.3 [**4c** + H]^+^. UV-vis (*λ*/nm *ε* (M^–1^ cm^–1^)): 283 (23295), 336 (28771), 346 (29238), and 362 (29676).

### (ADC^4-DMP^)P_3_ (**4d**)

Yield: 94% (0.93 g). Mp: 270–273 °C (decomp.). Elem. anal. (%), calcd for C_35_H_44_N_3_P_3_ (599.3): C, 70.10; H, 7.40; N, 7.01; found: C, 69.66; H, 7.18; N 6.59. ^1^H NMR (500 MHz, CD_2_Cl_2_, 298 K): *δ* = 7.56 (t, *J* = 7.8 Hz, 2H, *p*-C_6_*H*_3_), 7.35 (d, *J* = 7.8 Hz, 4H, *m*-C_6_*H*_3_), 6.90 (d, *J* = 9.0 Hz, 2H, C_6_*H*_4_), 6.28 (d, *J* = 9.0 Hz, 2H, C_6_*H*_4_), 2.88 (s, 6H, N(*CH*_3_)_2_), 2.67 (sept, *J* = 6.8 Hz, 4H, C*H*(CH_3_)_2_), 1.24 (d, *J* = 6.7 Hz, 12H, CH(C*H*_3_)_2_), and 0.97 (d, *J* = 6.8 Hz, 12H, CH(C*H*_3_)_2_) ppm. ^13^C{^1^H} NMR (126 MHz, CD_2_Cl_2_, 298 K): *δ* = 167.0 (d, *J*_P–C_ = 82.8 Hz, *C*P); 151.9 (N*C*N); 146.2, 138.4, 134.6, 131.4, 130.8, 126.3, 125.8, and 111.1 (*C*_6_H_3_ and *C*_6_H_4_); 40.0 (N(*C*H_3_)_2_); 29.6 (*C*H(CH_3_)_2_); 25.7 and 23.5 (CH(*C*H_3_)_2_) ppm. ^31^P{^1^H} NMR (202 MHz, CD_2_Cl_2_, 298 K): *δ* = 319.5 (t, *J*_P–P_ = 504 Hz) and 173.3 (d, *J*_P–P_ = 504 Hz) ppm. MS (ESI, positive mode): *m*/*z* = 600.3 [**4d** + H]^+^. UV-vis (*λ*/nm (*ε* M^–1^ cm^–1^)): 285 (37768), 322 (46655), 343 (47543), 366 (48310), and 398 (45288).

#### Experimental identification of the insoluble material

A mixture of the insoluble material (20 mg, 80 μmol, calcd for Li_3_P_7_) and IPrHCl (80 mg, 188 μmol) was stirred in 3 mL of THF for three days at rt, resulting in a dark red suspension. A black solid was removed by filtration and the filtrate was dried in a vacuum, affording a dark red solid which was identified as IPr=PH^17^ by NMR spectroscopy. ^1^H NMR (500 MHz, C_6_D_6_, 298 K): *δ* = 7.23 (t, *J* = 7.7 Hz, 2H, *p*-C_6_*H*_3_), 7.14 (d, *J* = 7.6 Hz, 4H, *m*-C_6_*H*_3_), 6.18 (s, 2H, NC*H*), 3.06 (sept, *J* = 6.7 Hz, 4H, C*H*(CH_3_)_2_), 1.92 (d, *J*_PH_ = 165.2 Hz, 1H, P*H*), 1.47 (d, *J* = 6.8 Hz, 12H, CH(C*H*_3_)_2_), and 1.15 (d, *J* = 6.9 Hz, 12H, CH(C*H*_3_)_2_) ppm. ^31^P NMR (C_6_D_6_, 298 K, 500 MHz): *δ* = –134.4 (d, *J*_P–H_ = 165.2 Hz) ppm. ^31^P{^1^H} NMR (C_6_D_6_, 298 K, 500 MHz): *δ* = –134.4 ppm.

### Alternative synthesis of **4a** and **4c** from iMICs^Ar^**2a** and **2c**

To a 15 mL THF suspension of **1a** (0.98 g, 1.8 mmol), *n*-BuLi (2.5 M, 0.8 mL, 2.0 mmol) was added at –40 °C. The resulting brown solution was stirred at –20 °C for 45 min and then for 15 min at rt. Subsequently, P_4_ (0.3 g, 2.4 mmol) was added in one portion and the resulting reaction mixture was stirred overnight at rt. The volatiles were removed under vacuum to obtain a dark residue, which was extracted with toluene (3 × 10 mL). The filtrate was dried in a vacuum to obtain **4a**. Yield: 41% (0.4 g).

#### (ADC^4-Tol^)P_3_ (**4c**)

Similarly, treatment of **3c** with P_4_ gave **4c**. Yield: 36% (0.4 g).

### Syntheses of complexes **5a**, **5b**, **6**, and **7**

#### [(ADC^Ph^)P_3_]Fe(CO)_4_ (**5a**)

To a mixture of **4a** (651 mg, 1.2 mmol) and Fe_2_(CO)_9_ (510 mg, 1.4 mmol), 30 mL THF was added at rt. The brown colored solution changed to a dark red colored solution after 15 min, which was further stirred overnight. The volatiles were removed in a vacuum to afford a red solid, which was extracted with 30 mL toluene. The volume of the filtrate was reduced to 10 mL and stored at –30° for one week to obtained orange needles of **5a** (696 mg, 80%), which were also suitable for X-ray diffraction. Mp: 167–172 °C (decomp.). Elem. anal. (%), calcd for C_37_H_39_FeN_2_O_4_P_3_ (724.5): C, 61.34; H, 5.43; N, 3.87; found: C, 59.66; H, 5.24; N, 3.71. ^1^H NMR (500 MHz, CD_2_Cl_2_, 298 K): *δ* = 7.60 (t, *J* = 7.8 Hz, 2H, *p*-C_6_*H*_3_), 7.38 (d, *J* = 7.8 Hz, 4H, *m*-C_6_*H*_3_), 7.34 (t, *J* = 7.5 Hz, 1H, *p*-C_6_*H*_5_), 7.22–7.15 (m, 4H, *o*-, *m*-C_6_*H*_5_), 2.57 (sept, *J* = 6.7 Hz, 4H, C*H*(CH_3_)_2_), 1.27 (d, *J* = 6.7 Hz, 12H, CH(C*H*_3_)_2_), and 1.01 (d, *J* = 6.8 Hz, 12H, CH(C*H*_3_)_2_) ppm. ^13^C{^1^H} NMR (126 MHz, CD_2_Cl_2_, 298 K): *δ* = 215.1 (*C*O); 161.7 (d, *J*_P–C_ = 70.3 Hz, *C*P); 146.0 (N*C*N), 133.1, 132.2, 129.7, 129.5, 129.3, 128.7, 126.1, and 123.1 (*C*_6_H_3_ and *C*_6_H_5_); 29.8 (*C*H(CH_3_)_2_); 26.0 and 23.5 (CH(*C*H_3_)_2_) ppm. ^31^P{^1^H} NMR (202 MHz, CD_2_Cl_2_, 298 K): *δ* = 316.8 (t, *J*_P–P_ = 531 Hz) and 145.4 (d, *J*_P–P_ = 531 Hz) ppm. MS (ESI, positive mode): *m*/*z* = 725.1 [**5a** + H]^+^. UV-vis (*λ*/nm *ε* (M^–1^ cm^–1^)): 285 (33061), 328 (31345), and 428 (37184). IR (ATR, diamond): ν̃/cm^–1^ = 2041, 1966, 1937, and 1919.

#### [(ADC^3-Tol^)P_3_]Fe(CO)_4_ (**5b**)

Compound **5b** was synthesized following a similar procedure to that described above for **5a** using **4b** (300 mg, 0.53 mmol) and Fe_2_(CO)_9_ (191 mg, 0.53 mmol) as an orange crystalline solid. Yield: 84% (333 mg). Crystals suitable for X-ray diffraction were obtained by storing a saturated toluene solution of **5b** overnight at rt. Mp: 180–182 °C (decomp.) Elem. anal. (%), calcd for **5b**, C_38_H_41_FeN_2_O_4_P_3_ (738.5): C, 61.80; H, 7.56; N, 3.79; found C, 62.69; H, 5.95; N, 3.45. ^1^H NMR (500 MHz, CD_2_Cl_2_, 298 K): *δ* = 7.59 (t, *J* = 7.8 Hz, 2H, *p*-C_6_*H*_3_), 7.37 (d, *J* = 7.8 Hz, 4H, *m*-C_6_*H*_3_), 7.15 (d, *J* = 7.4 Hz, 1H, *o*-C_6_*H*_4_), 7.08 (t, *J* = 7.7 Hz, 1H, *m*-C_6_*H*_4_), 7.00 (s, 1H, *o*-C_6_*H*_4_), 6.94 (d, *J* = 7.7 Hz, 1H, *p*-C_6_*H*_4_), 2.56 (sept, *J* = 6.6 Hz, 4H, C*H*(CH_3_)_2_), 2.09 (s, 3H, C*H*_3_), 1.27 (d, *J* = 6.6 Hz, 12H, CH(C*H*_3_)_2_), and 1.04 (d, *J* = 6.7 Hz, 12H, CH(C*H*_3_)_2_) ppm. ^13^C{^1^H} NMR (126 MHz, CD_2_Cl_2_, 298 K): *δ* = 215.1 and 214.9 (*C*O); 161.6 (d, *J*_P–C_ = 71 Hz, N*C*P); 147.6 (N*C*N); 146.1, 139.5, 133.1, 133.0, 132.1, 130.3, 129.9, 129.0, 126.8, 126.0, and 122.9 (*C*_6_H_3_ and *C*_6_H_5_); 29.8 (*C*H(CH_3_)_2_); 26.1 and 23.6 (CH(*C*H_3_)_2_); 21.2 (*C*H_3_) ppm. ^31^P{^1^H} NMR (202 MHz, CD_2_Cl_2_, 298 K): *δ* = 315.5 (t, *J*_P–P_ = 531 Hz) and 145.4 (d, *J*_P–P_ = 532 Hz) ppm. MS (ESI, positive mode): *m*/*z* = 739.1 [**5b** + H]^+^. UV-vis (*λ*/nm *ε* (M^–1^ cm^–1^)): 282 (35189), 327 (29758), and 416 (37568). IR (ATR, diamond): ν̃/cm^–1^ = 2039, 2007, 1962, and 1921.

#### [(ADC^Ph^)P_3_]Mo(CO)_5_ (**6**)

To a mixture of **4a** (447 mg, 0.8 mmol) and Mo(CO)_6_ (212 mg, 0.8 mmol), 20 mL THF was added at rt. The yellow suspension was stirred for three days at 60 °C. Filtration through a plug of Celite afforded an orange solution. The volatiles were removed under vacuum to obtain **6** as a yellow solid (523 mg, 81%). Crystals suitable for X-ray diffraction were obtained by a slow diffusion of *n*-hexane into a saturated toluene solution of **6**. Elem. anal. (%), calcd for **6**, C_38_H_39_MoN_2_O_5_P_3_ (792.6): C, 57.58; H, 4.96; N, 3.53; found: C, 57.06; H, 4.73; N, 3.25. ^1^H NMR (500 MHz, CD_2_Cl_2_, 298 K): *δ* = 7.60 (t, *J* = 7.8 Hz, 2H, *p*-C_6_*H*_3_), 7.38 (d, *J* = 7.8 Hz, 4H, *m*-C_6_*H*_3_), 7.35 (t, *J* = 7.6 Hz, 1H, *p*-C_6_*H*_5_), 7.21 (t, *J* = 7.8 Hz, 2H, *m*-C_6_*H*_5_), 7.17 (d, *J* = 7.9 Hz, 2H, *o*-C_6_*H*_5_), 2.58 (sept, *J* = 6.7 Hz, 4H, C*H*(CH_3_)_2_), 1.27 (d, *J* = 6.7 Hz, 12H, CH(C*H*_3_)_2_), and 1.02 (d, *J* = 6.8 Hz, 12H, CH(C*H*_3_)_2_). ^13^C{^1^H} NMR (126 MHz, CD_2_Cl_2_, 298 K): *δ* = 206.0 and 201.8 (*C*O); 164.4 (d, *J*_P–C_ = 73 Hz, N*C*P); 146.1 (N*C*N); 133.2, 132.2, 132.1, 129.7, 129.2, and 126.1 (*C*_6_H_3_ and *C*_6_H_5_); 29.8 (*C*H(CH_3_)_2_); 26.0 and 23.6 (CH(*C*H_3_)_2_) ppm. ^31^P{^1^H} NMR (202 MHz, CD_2_Cl_2_, 298 K): *δ* = 299.1 (t, *J*_P–P_ = 510 Hz) and 160.2 (d, *J*_P–P_ = 511 Hz) ppm. IR (ATR, diamond): ν̃/cm^–1^ = 2065, 2051, 1945, 1925, and 1911.

#### [(ADC^4-DMP^)P_3_]W(CO)_5_ (**7**)

A 10 mL THF solution of W(CO)_6_ (212 mg, 0.8 mmol) was irradiated under UV light for 3 h and then combined with a 6 mL THF solution of **4d** (447 mg, 0.8 mmol). The yellow solution was stirred overnight at rt. The volatiles were removed under vacuum to obtain **7** as a yellow solid (256 mg, 88%). Crystals suitable for X-ray diffraction were obtained by slow evaporation of a saturated toluene solution of **7** at rt. Elem. anal. (%), calcd for **7**, C_40_H_44_N_3_O_5_P_3_W (923.6): C, 52.02; H, 4.80; N, 4.55; found C, 51.40; H, 4.39; N, 4.10. ^1^H NMR (500 MHz, CD_2_Cl_2_, 298 K): *δ* = 7.61 (t, *J* = 7.8 Hz, 2H, *p*-C_6_*H*_3_), 7.41 (d, *J* = 7.8 Hz, 4H, *m*-C_6_*H*_3_), 6.91 (d, *J* = 9.2 Hz, 2H, C_6_*H*_4_), 6.33 (d, *J* = 9.2 Hz, 2H, C_6_*H*_4_), 2.88 (s, 6H, N(C*H*_3_)_2_), 2.60 (sept, *J* = 6.8 Hz, 4H, C*H*(CH_3_)_2_), 1.26 (d, *J* = 6.9 Hz, 12H, CH(C*H*_3_)_2_), and 1.00 (d, *J* = 6.8 Hz, 12H, CH(C*H*_3_)_2_) ppm. ^13^C{^1^H} NMR (126 MHz, CD_2_Cl_2_, 298 K): *δ* = 197.3 and 192.0 (*C*O); 152.1 (N*C*P); 146.1 (N*C*N); 134.3, 131.8, 130.7, 129.9, 126.1, 111.2, and 108.7 (*C*_6_H_3_ and *C*_6_H_5_); 40.0 (N(*C*H_3_)_2_); 29.7 (*C*H(CH_3_)_2_); 25.6 and 23.6 (CH(*C*H_3_)_2_) ppm. ^31^P{^1^H} NMR (202 MHz, CD_2_Cl_2_, 298 K): *δ* = 250.9 (t, *J*_P–P_ = 512 Hz, with ^183^W satellites, *J*_W–P_ = 202 Hz) and 157.0 (d, *J*_P–P_ = 505 Hz) ppm. MS (ESI): *m*/*z* = 924.2 [**7** + H]^+^. IR (ATR, diamond): ν̃/cm^–1^ = 2063, 1978, 1925, and 1907.

## Conclusions

In conclusion, the direct functionalization of white phosphorus (P_4_) with anionic dicarbenes (ADCs) (**2a–2d**) as well as with mesoionic carbenes (iMICs^Ar^) (**3a** and **3c**) that leads to the formation of unique 1,2,3-triphosphol-2-ide derivatives **4a–4d** as crystalline solids up to 98% yield has been reported. The isolation of C_2_P_3_-heterocycles **4a–4d** is unprecedented in the P_4_ activation by singlet carbenes and main-group compounds. The formation of **4a–4d** suggests unique [3 + 1] fragmentation of P_4_ into P_3_^+^ and P^–^. The former species combines with an ADC to give **4a–4d**, whereas the latter reacts with additional P_4_ to form (P_7_)^3–^ that can be isolated as Li_3_P_7_. Electronic structures of **4a–4d** have been analyzed by computational studies, which, along with the crystallographic data, show that both C_3_N_2_- and C_2_P_3_-rings of **4a–4d** are 6π-electron aromatic systems. Thus, **4a–4d** can be considered as neutral analogues of cyclopentadienyl anions. The C_2_P_3_-ring of **4a–4d** is negatively polarized towards the central phosphorus atom, and hence **4a–4d** may also function as potent two-electron σ-donor ligands. This feature has been demonstrated with the isolation of transition metal complexes **5a**, **5b**, **6**, and **7**. Consequently, **4a–4d** have interesting perspectives as ligands in main-group element as well as transition-metal chemistry and catalysis. Further investigations in this direction are currently underway in this laboratory.

## Conflicts of interest

There are no conflicts to declare.

## Supplementary Material

Supplementary information

Crystal structure data
