# Supplementary material for: Direct functionalization of white phosphorus with anionic dicarbenes and mesoionic carbenes: facile access to 1,2,3-triphosphol-2-ides
Source: Chem Sci. 2019 Oct 18;10(48):11078–85. doi: 10.1039/c9sc04441h (PMC7069227; doi:10.1039/c9sc04441h)
Supplement: Supplementary file 1 [file SC-010-C9SC04441H-s001.pdf]

Electronic Supplementary Material (ESI) for Chemical Science.
This journal is © The Royal Society of Chemistry 2019

Electronic Supplementary Information (ESI)

for

Direct functionalization of white phosphorus with anionic dicarbenes and mesoionic carbenes: Facile access to 1,2,3-triphosphol-2-ides

Dennis Rottschäfer, Beate Neumann, Hans-Georg Stammler, Sebastian Blomeyer, and Rajendra S. Ghadwal*

Molecular Inorganic Chemistry and Catalysis, Inorganic and Structural Chemistry, Center for Molecular Materials, Faculty of Chemistry, Universität Bielefeld,
Universitätsstr. 25, D-33615, Bielefeld, Germany.

E-mail: rghadwal@uni-bielefeld.de

Homepage: www.ghadwalgroup.de.

Contents:

Plots of NMR spectra	p. 2 – 19
UV-visible spectroscopy	p. 20 – 22
Infrared spectroscopy	p. 23 – 26
X-Ray diffraction studies	p. 27 – 35
Computational details	p. 36 – 60
References	p. 61

Plots of NMR spectra

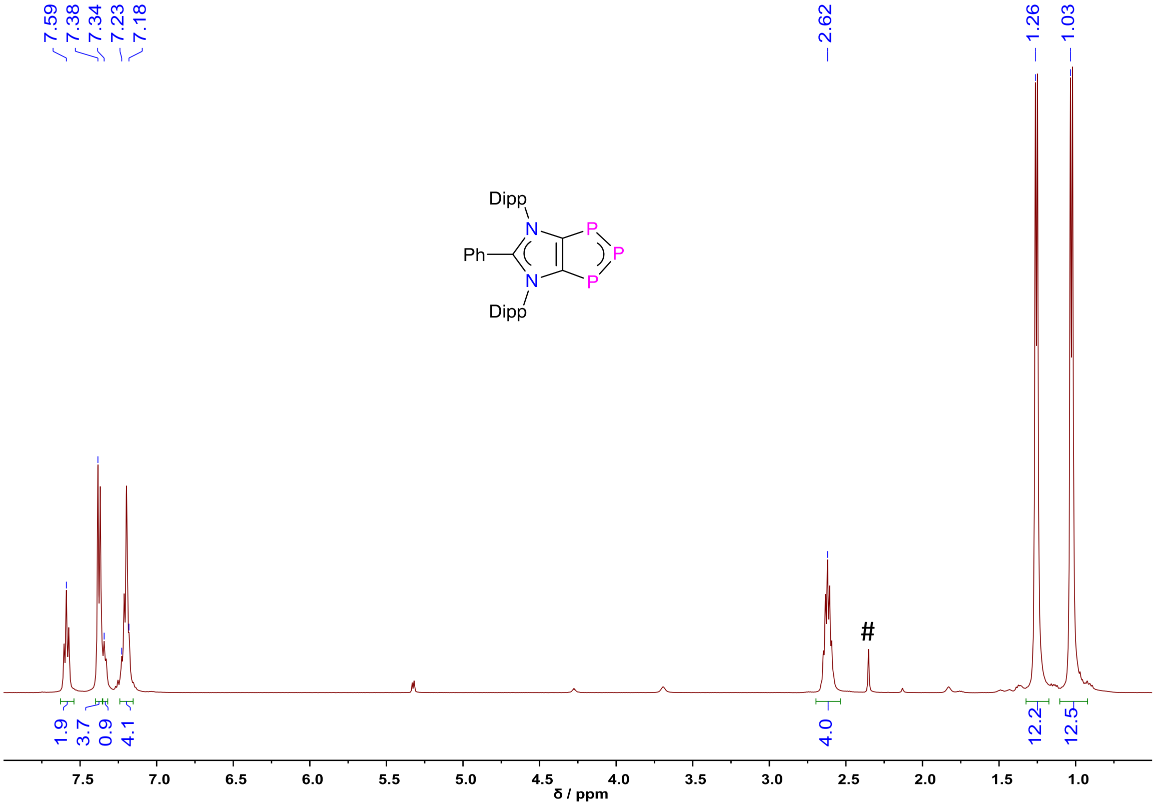

Fig. S1 ^{1}H NMR (500 MHz, CD_2Cl_2, 298 K) spectrum of compound **4a**. # = toluene.

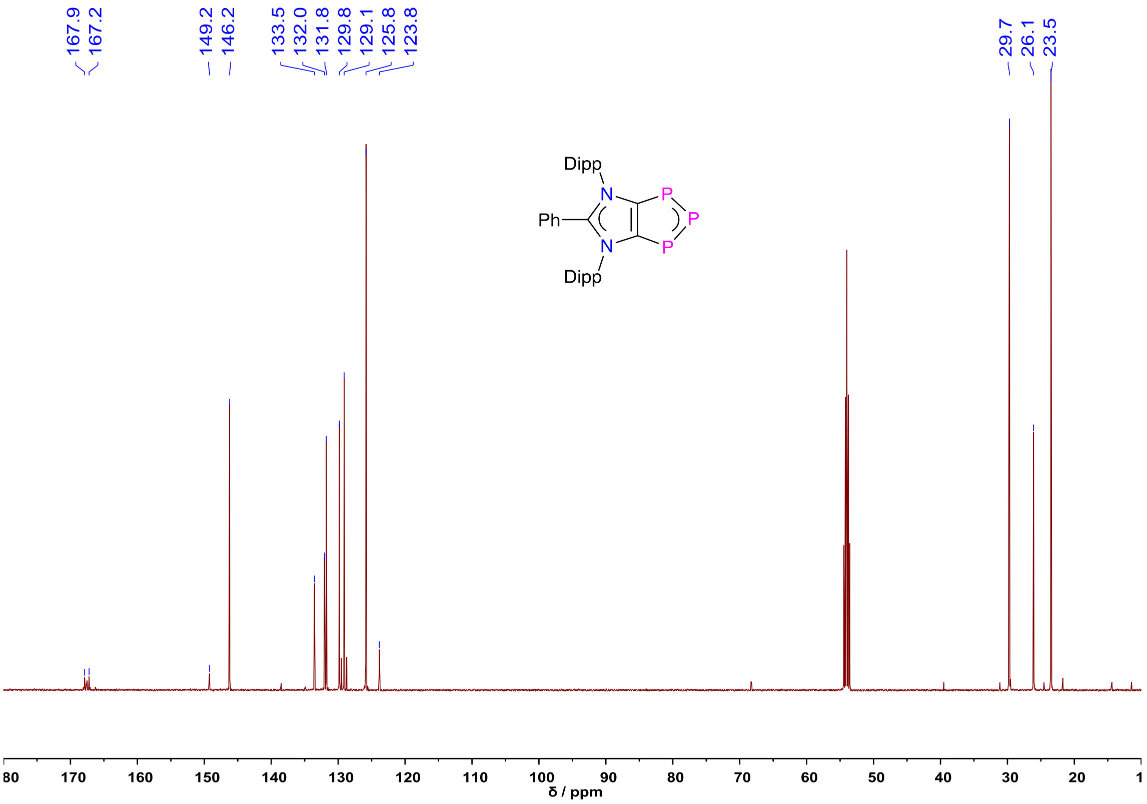

Fig. S2 ^{13}C NMR (125 MHz, CD_2Cl_2, 298 K) spectrum of compound **4a**.

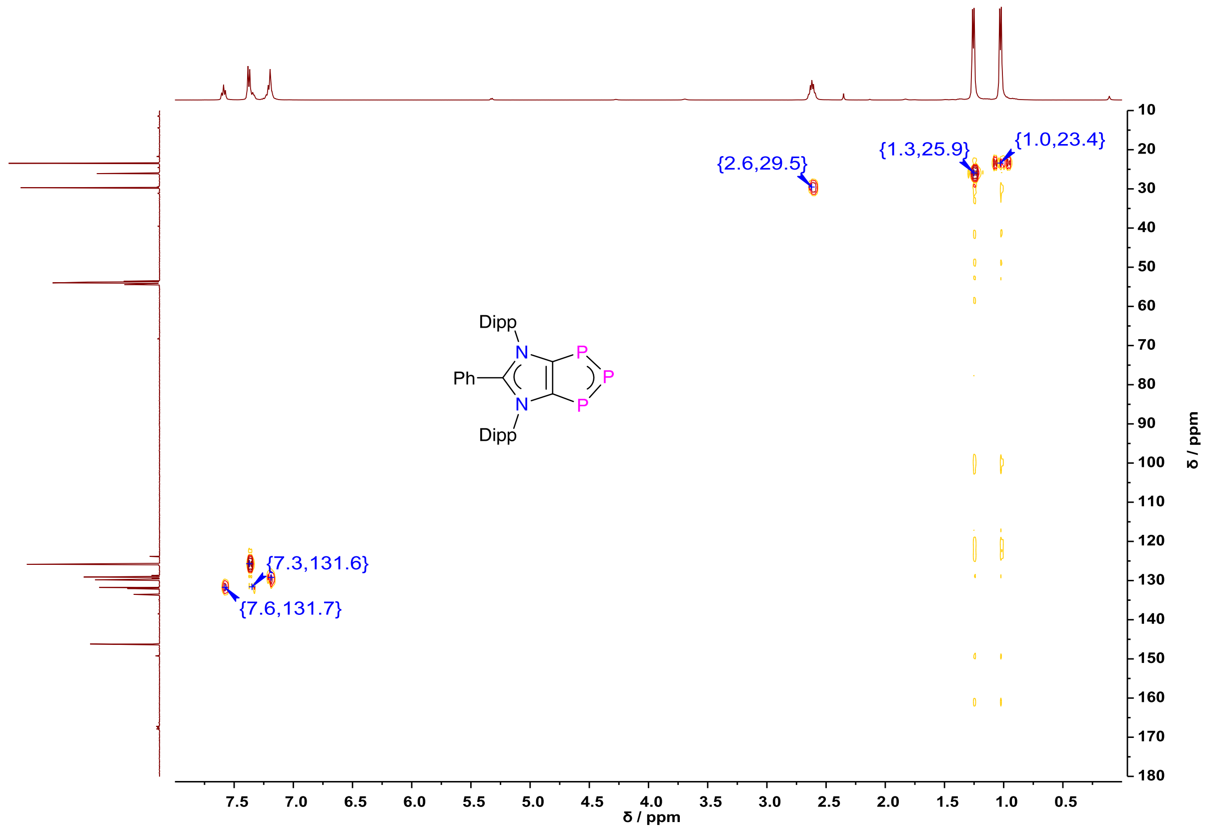

Fig. S3 ^{1}H-^{13}C-HMQC (500 MHz, CD_2Cl_2, 298 K) spectrum of compound **4a**.

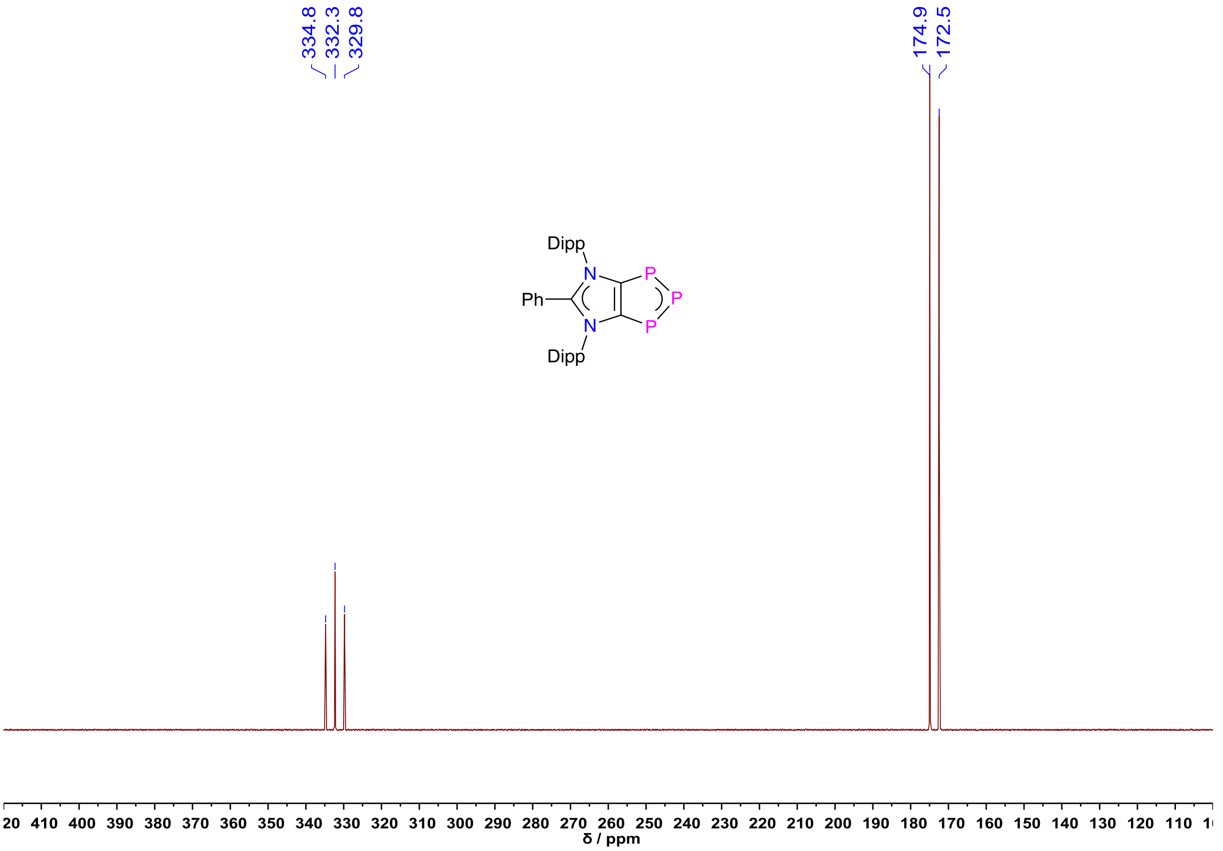

Fig. S4 $^{31}P\{^{1}H\}$ NMR (202 MHz, CD_2Cl_2, 298 K) spectrum of compound **4a**.

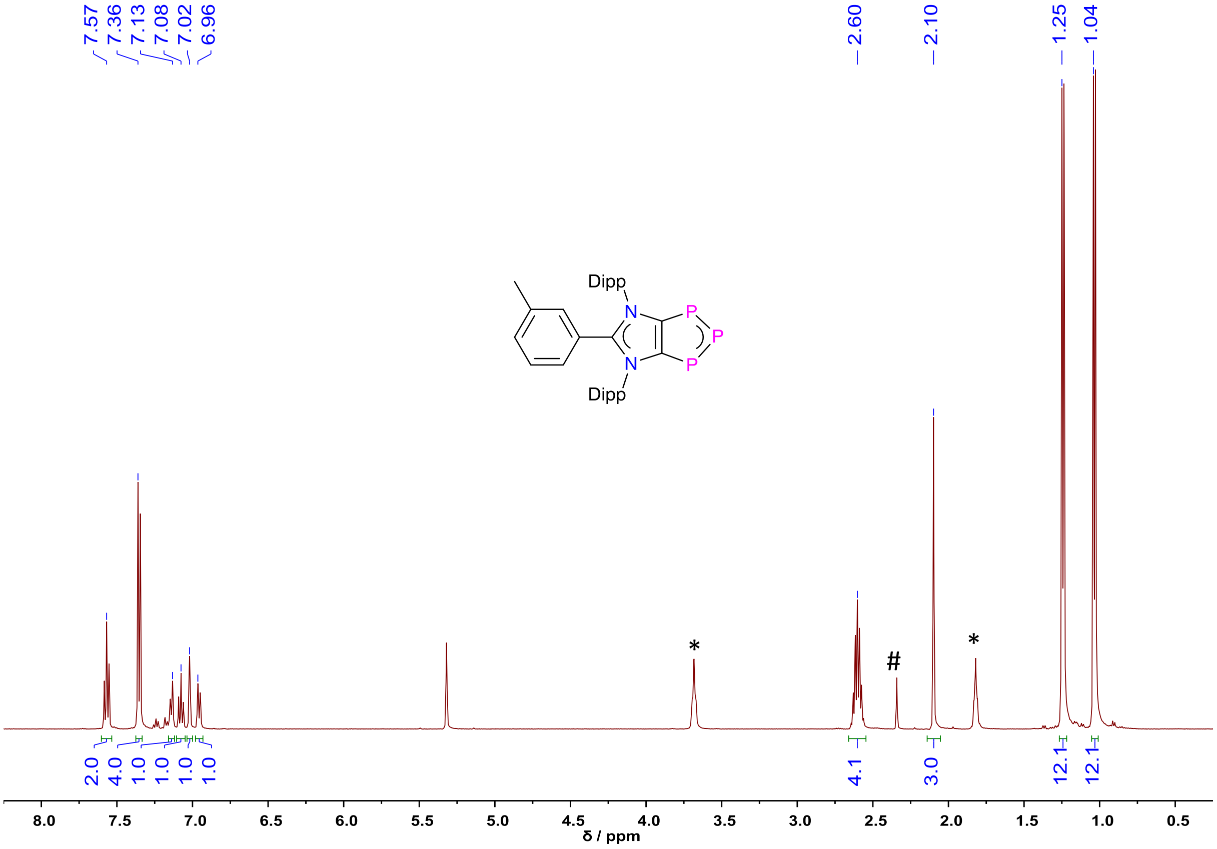

Fig. S5 ^{1}H NMR (500 MHz, CD_2Cl_2, 298 K) spectrum of compound **4b**. # = toluene, * = THF.

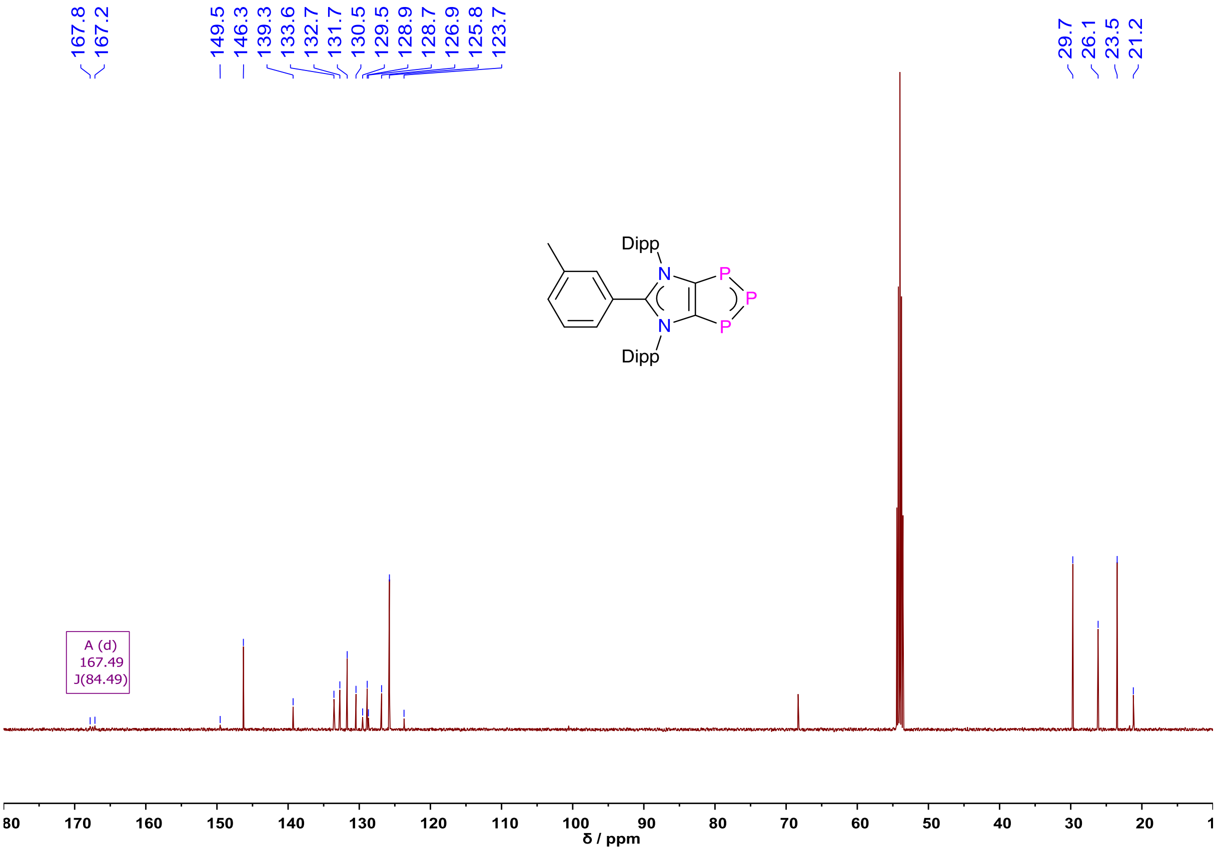

Fig. S6 ^{13}C NMR (125 MHz, CD_2Cl_2, 298 K) spectrum of compound **4b**.

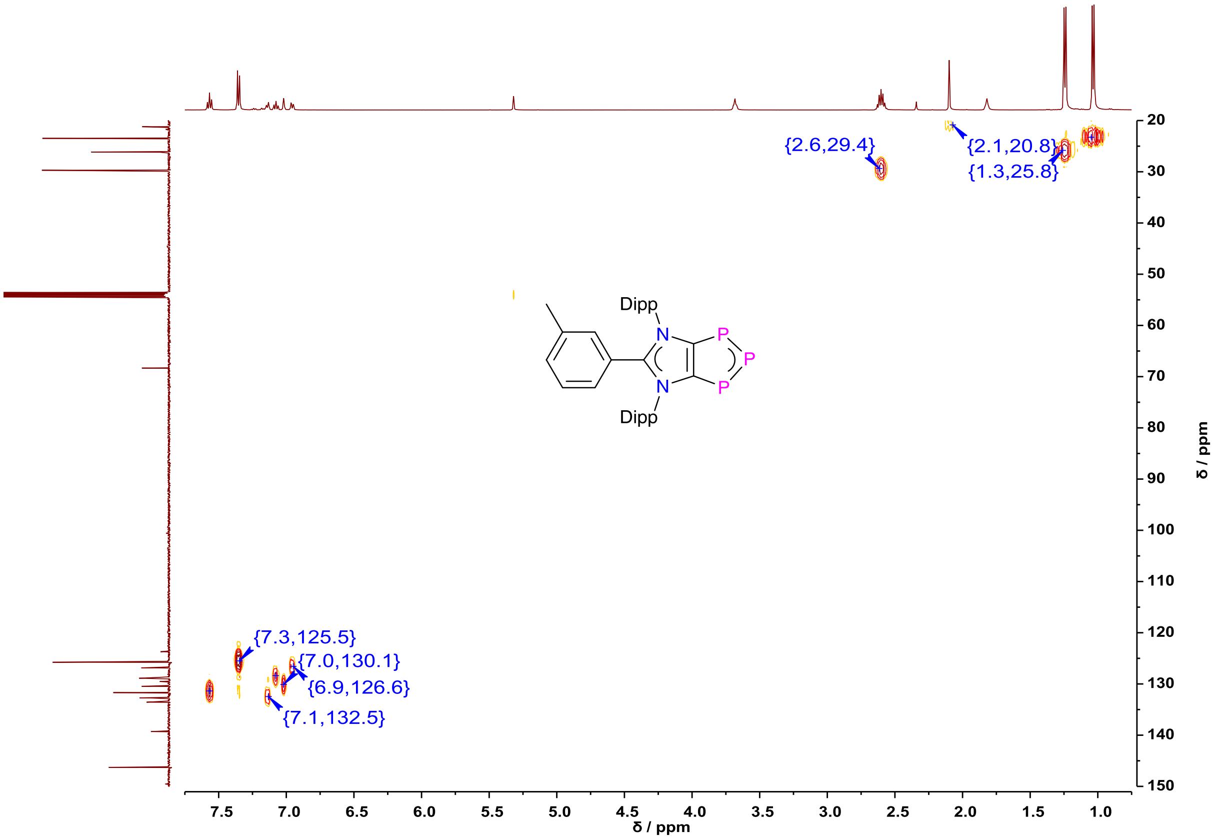

Fig. S7 ^{1}H-^{13}C-HMQC (500 MHz, CD_2Cl_2, 298 K) spectrum of compound **4b**.

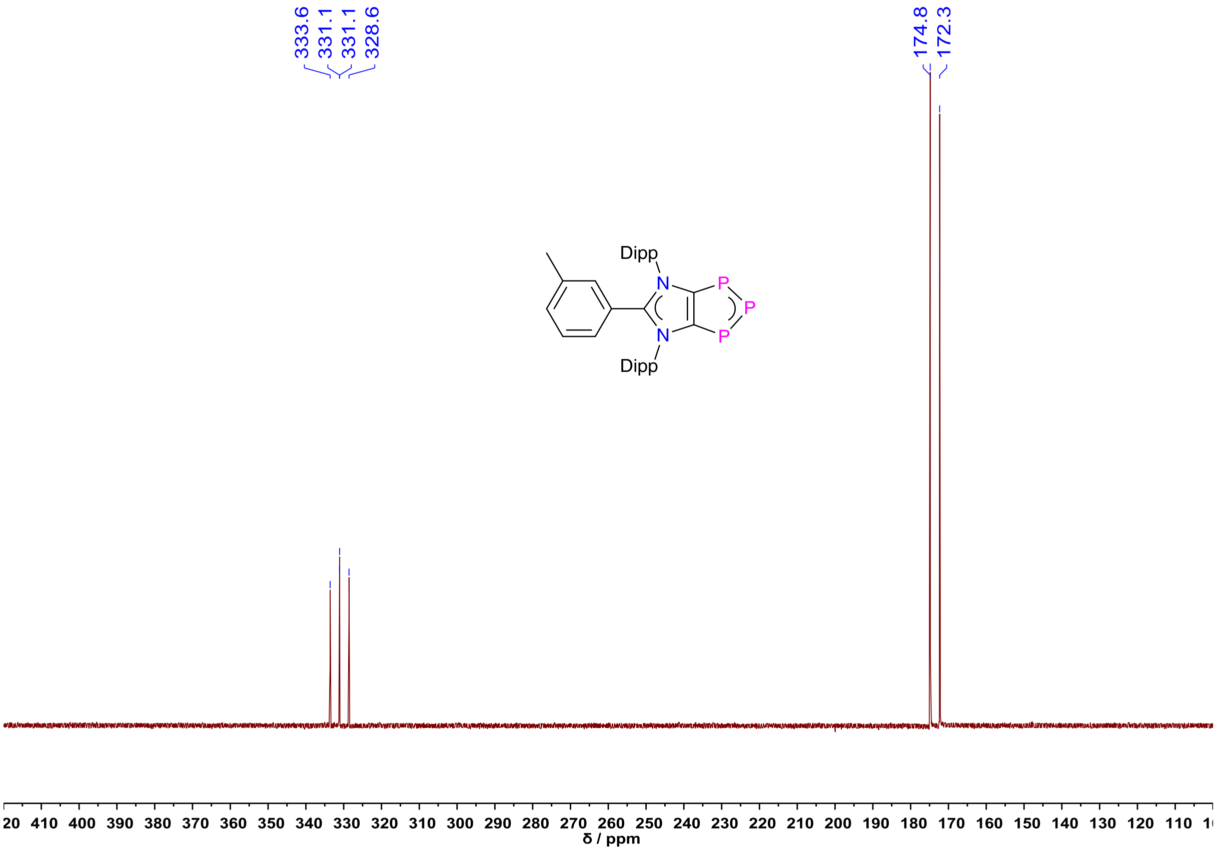

Fig. S8 $^{31}P\{^{1}H\}$ NMR (202 MHz, CD_2Cl_2, 298 K) spectrum of compound **4b**.

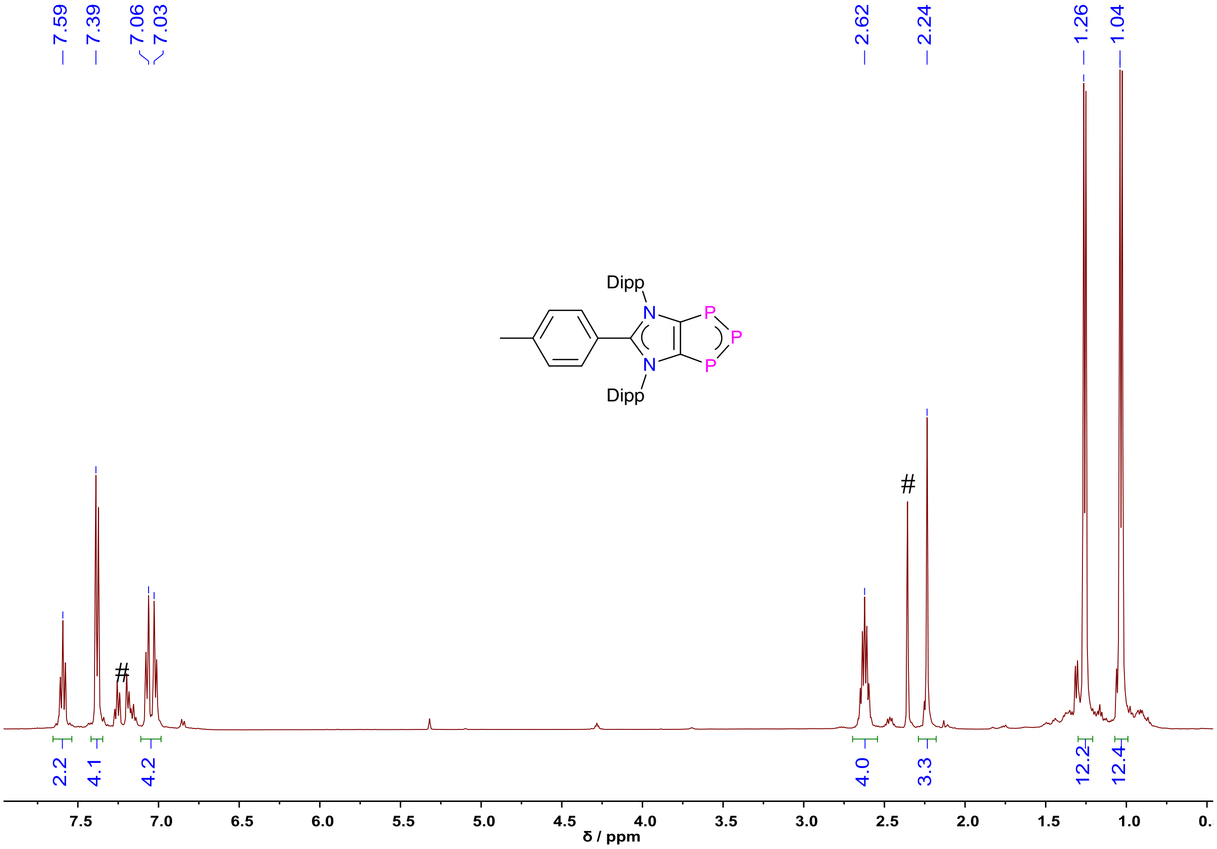

Fig. S9 ^{1}H NMR (500 MHz, CD_2Cl_2, 298 K) spectrum of compound **4c.** # = toluene.

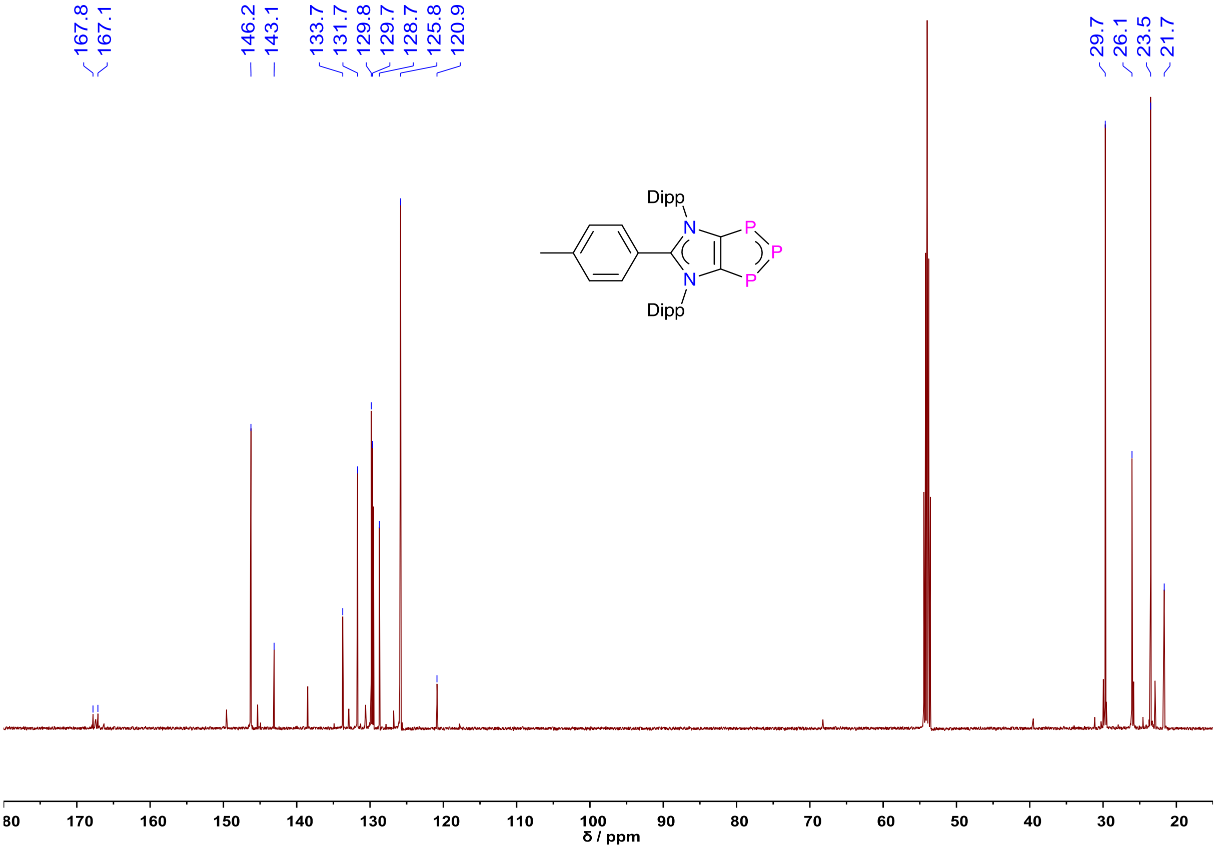

Fig. S10 ^{13}C NMR (125 MHz, CD_2Cl_2, 298 K) spectrum of compound **4c**.

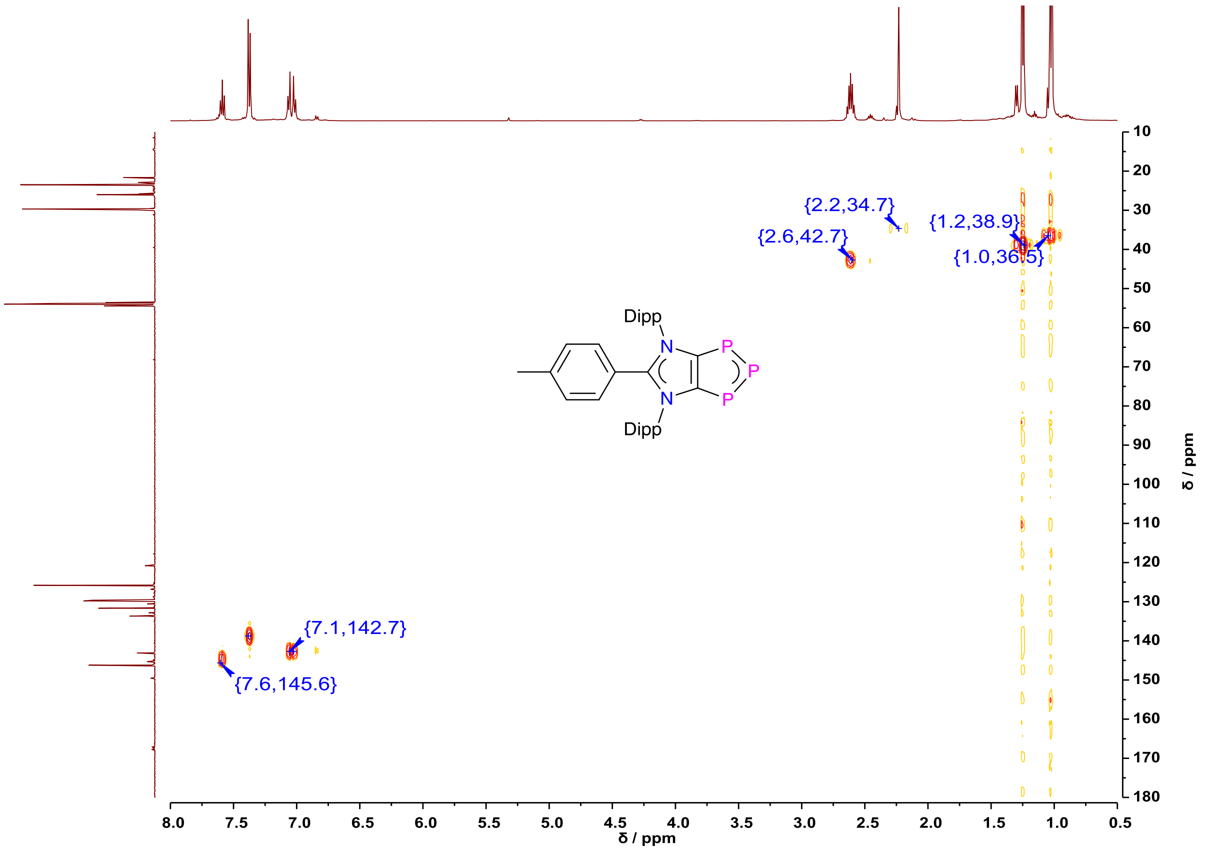

Fig. S11 ^{1}H-^{13}C-HMQC (500 MHz, CD_2Cl_2, 298 K) spectrum of compound **4c**.

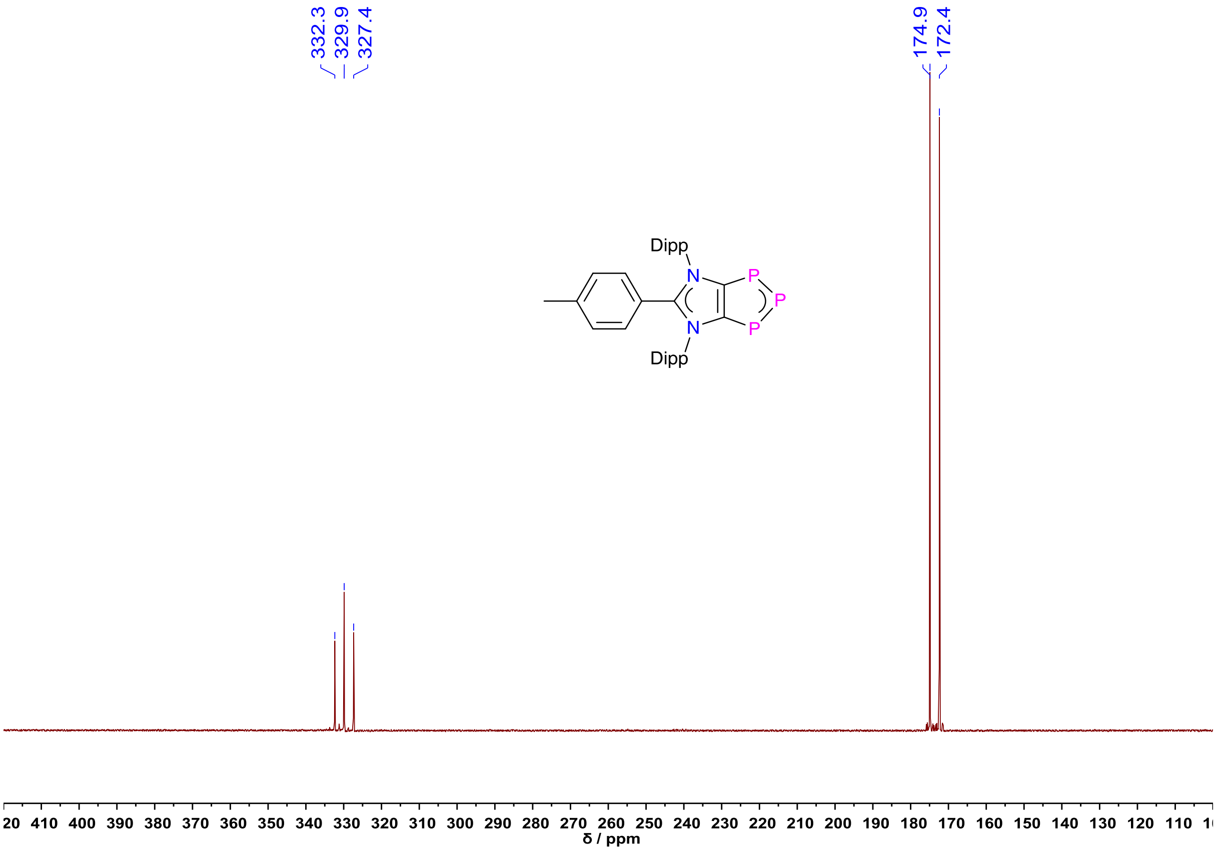

Fig. S12 ^{31}P{^{1}H} NMR (202 MHz, CD_2Cl_2, 298 K) spectrum of compound **4c**.

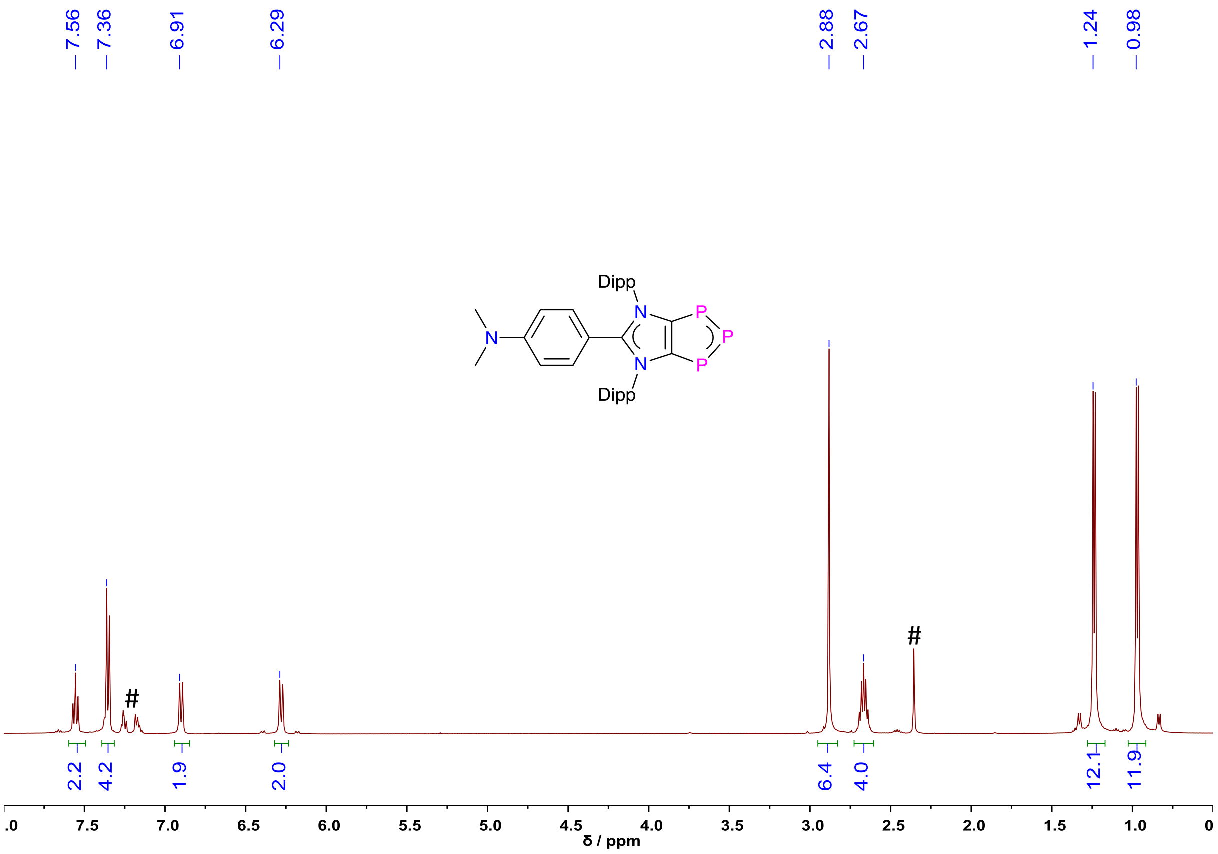

Fig. S13 ^{1}H NMR (500 MHz, CD_2Cl_2, 298 K) spectrum of compound **4d**. # = toluene.

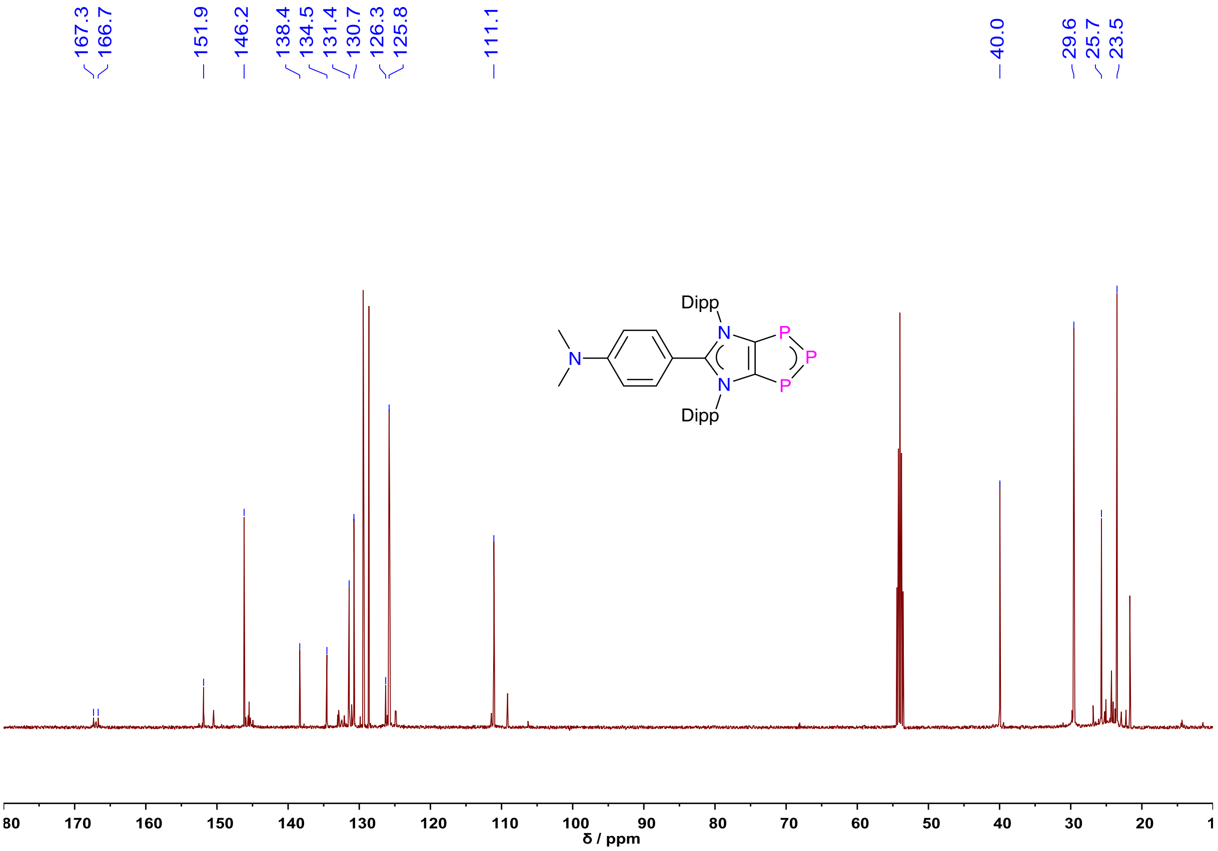

Fig. S14 ^{13}C NMR (125 MHz, CD_2Cl_2, 298 K) spectrum of compound **4d**.

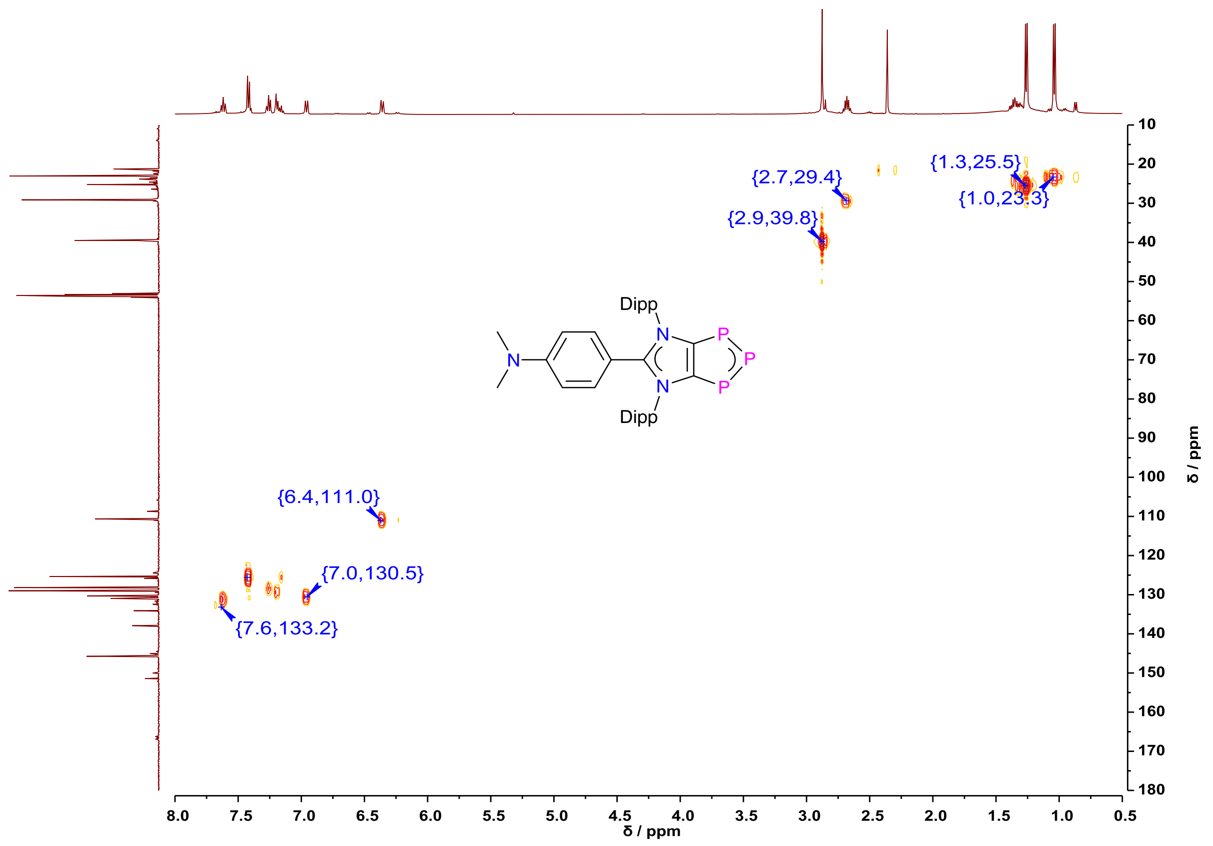

Fig. S15 ^{1}H-^{13}C-HMQC (500 MHz, CD_2Cl_2, 298 K) spectrum of compound **4d**.

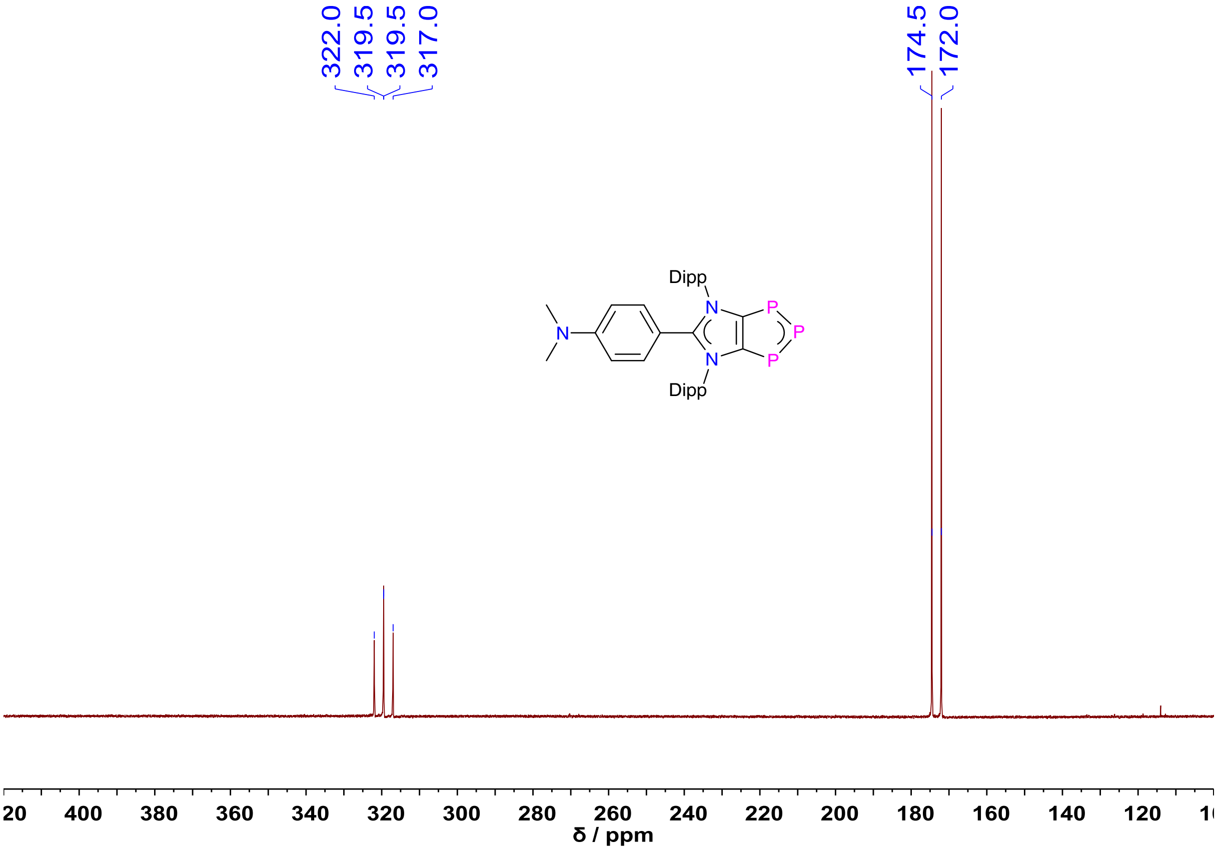

Fig. S16 $^{31}P\{^{1}H\}$ NMR (202 MHz, CD_2Cl_2, 298 K) spectrum of compound **4d**.

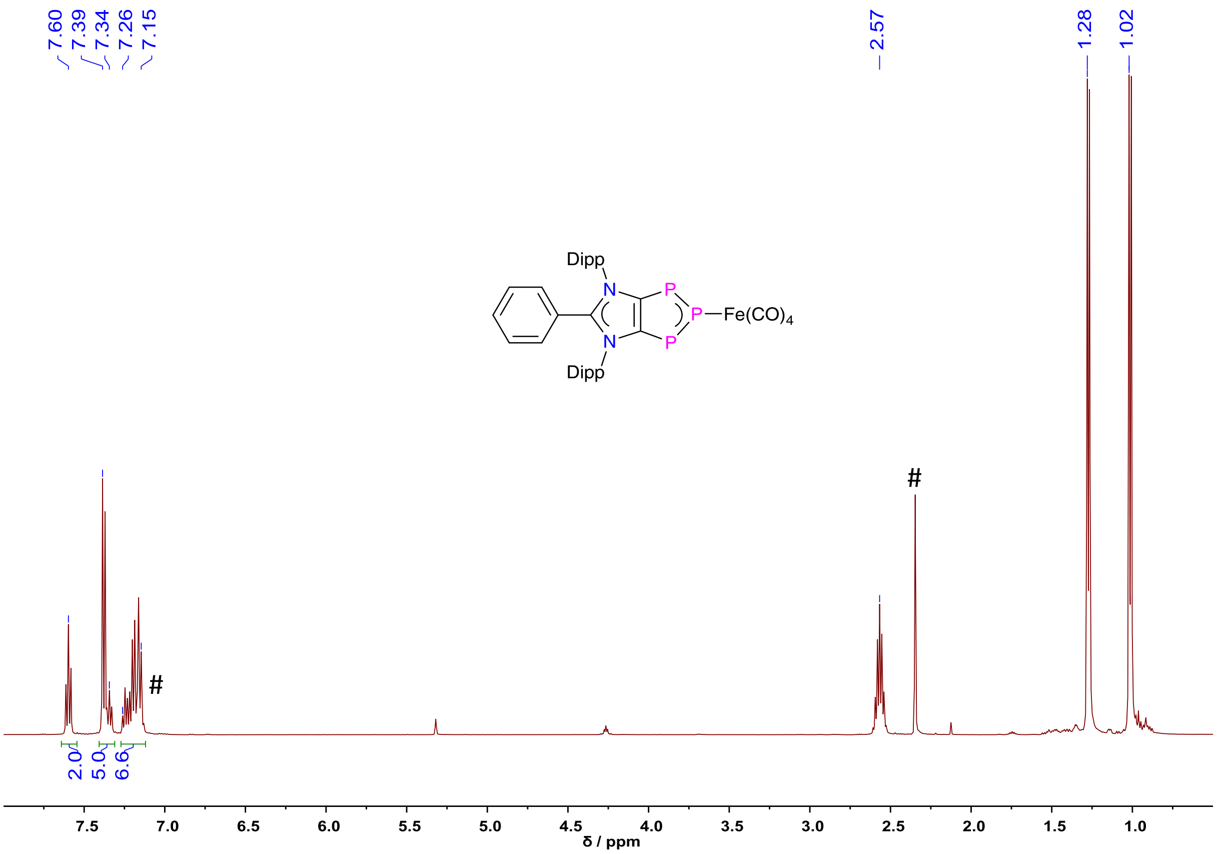

Fig. S17 ^{1}H NMR (500 MHz, CD_2Cl_2, 298 K) spectrum of compound **5a**. # = toluene.

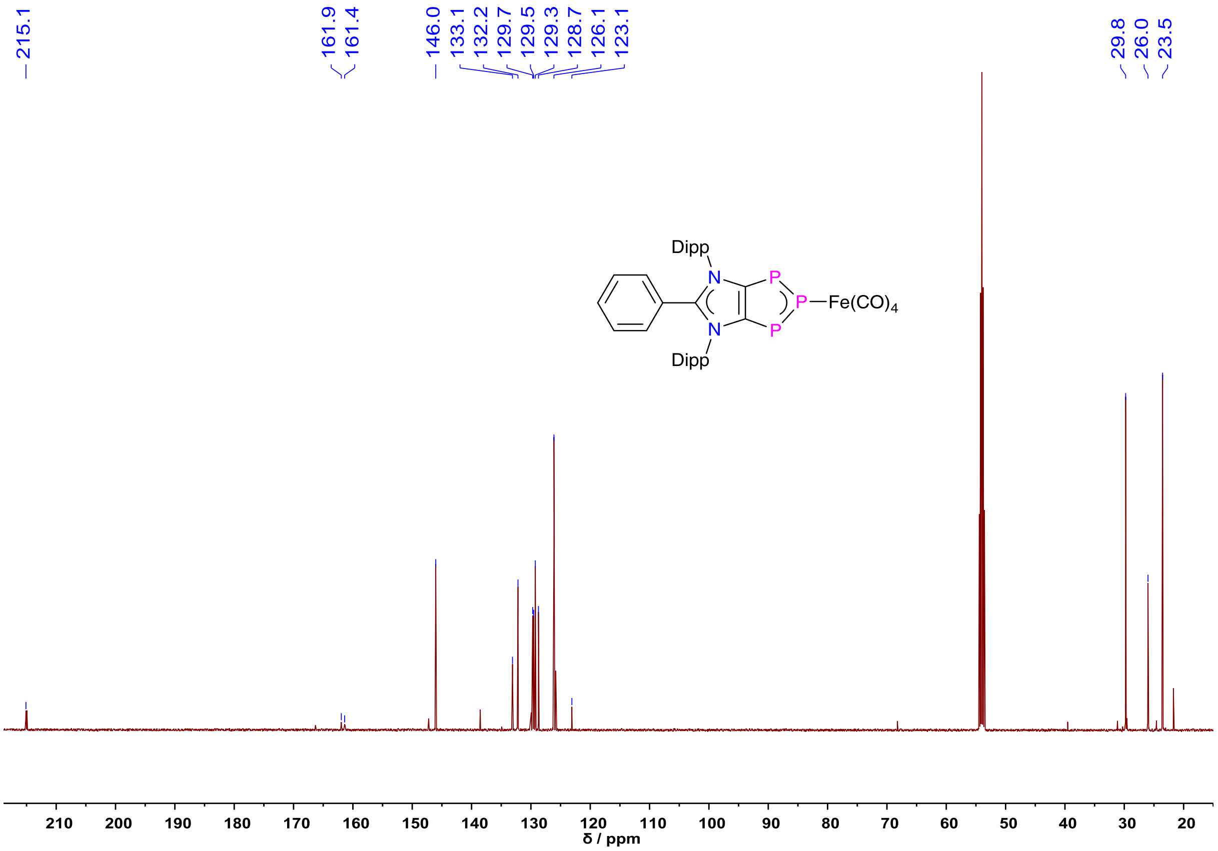

Fig. S18 ^{13}C NMR (125 MHz, CD_2Cl_2, 298 K) spectrum of compound **5a**.

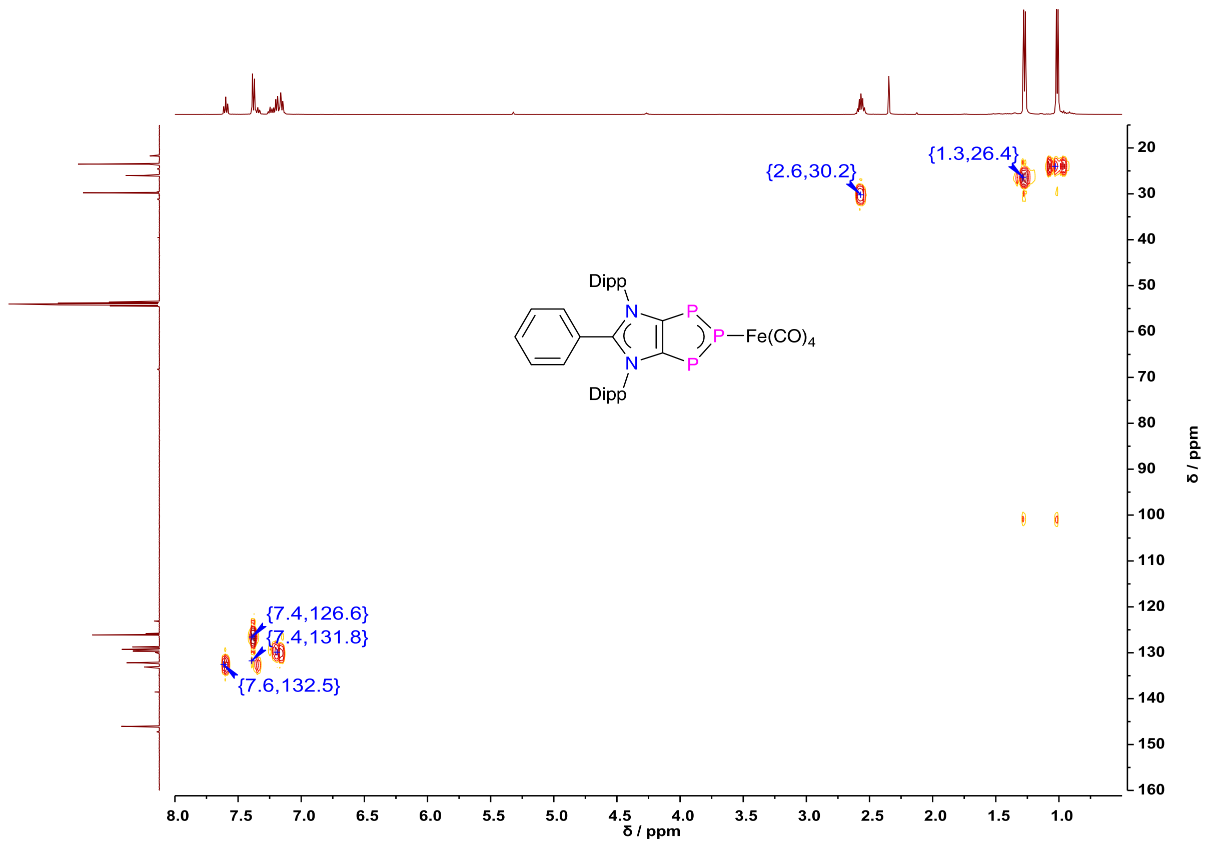

Fig. S19 ^{1}H-^{13}C-HMQC (500 MHz, CD_2Cl_2, 298 K) spectrum of compound **5a**.

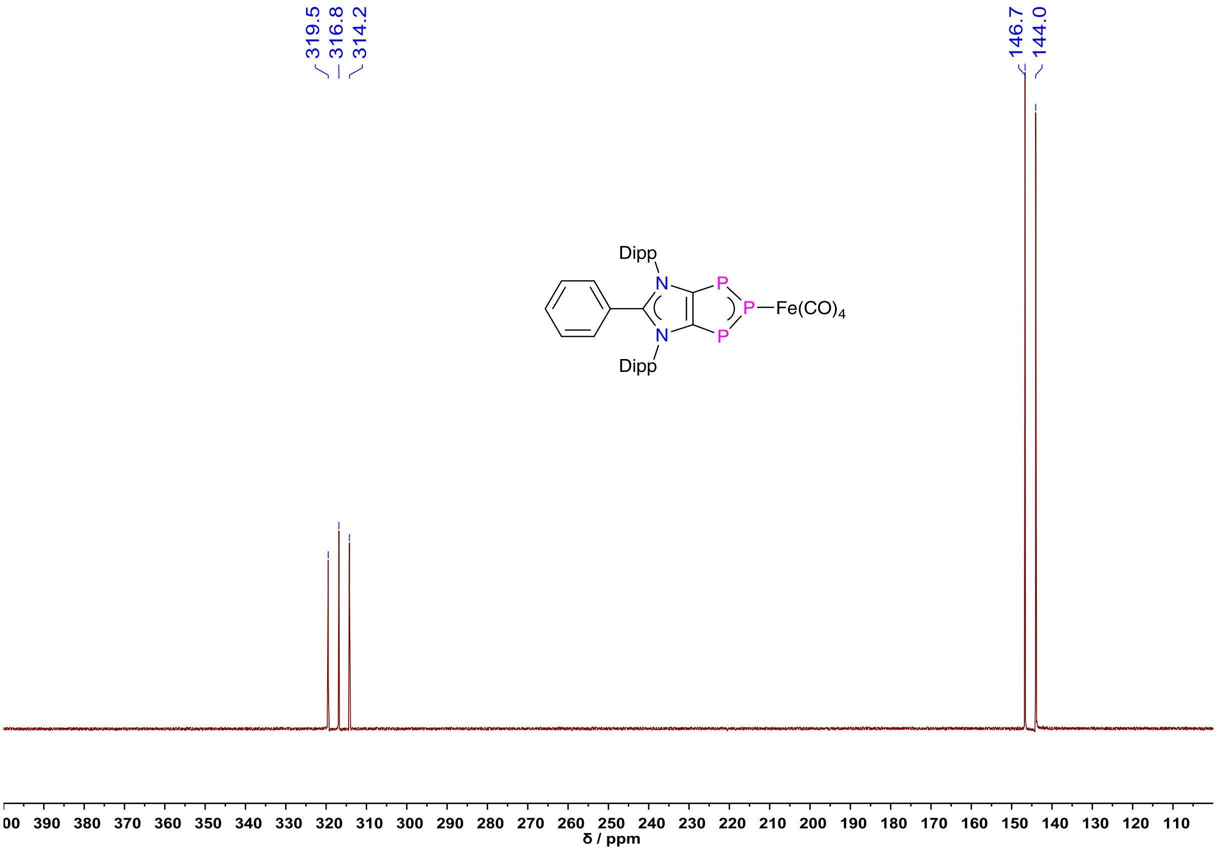

Fig. S20 $^{31}P\{^{1}H\}$ NMR (202 MHz, CD_2Cl_2, 298 K) spectrum of compound **5a**.

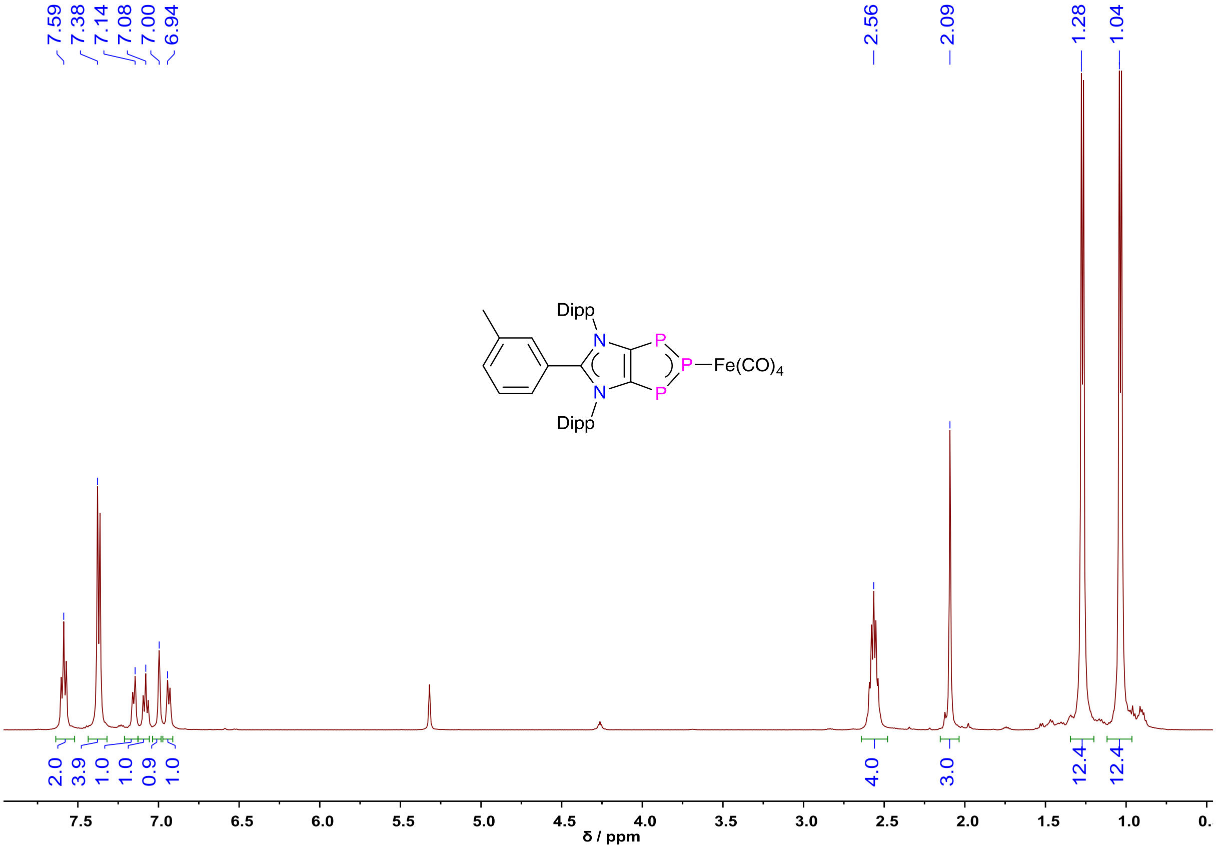

Fig. S21 ^{1}H NMR (500 MHz, CD_2Cl_2, 298 K) spectrum of compound **5b**.

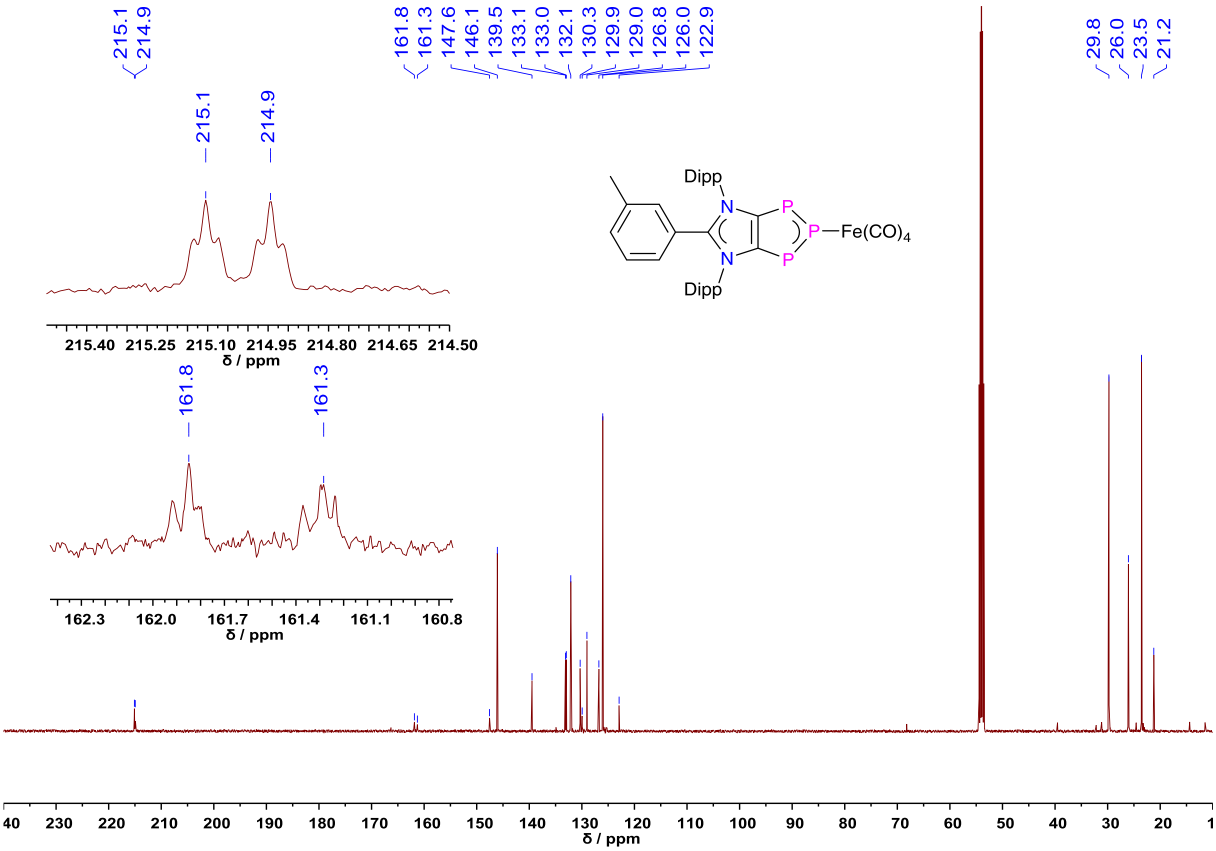

Fig. S22 ^{13}C NMR (125 MHz, CD_2Cl_2, 298 K) spectrum of compound **5b**.

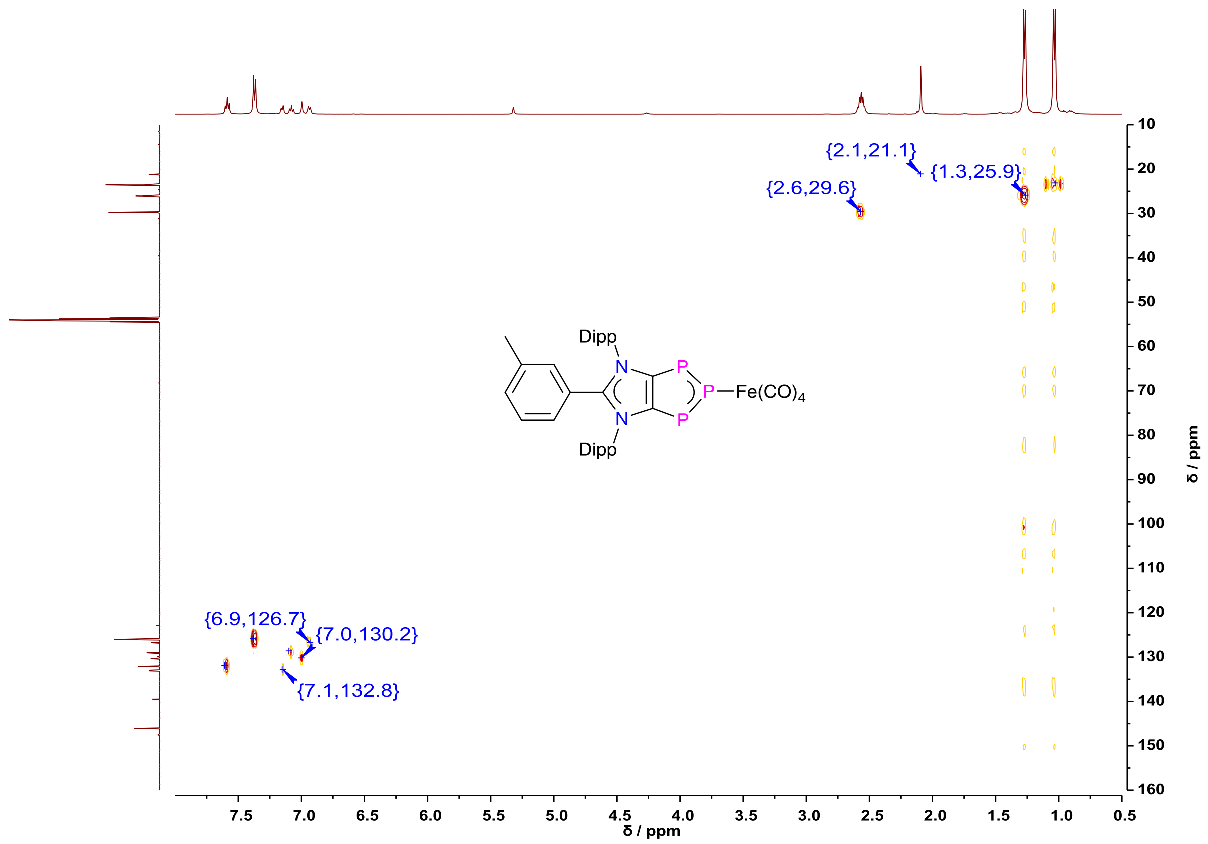

Fig. S23 ^{1}H-^{13}C-HMQC (500 MHz, CD_2Cl_2, 298 K) spectrum of compound **5b**.

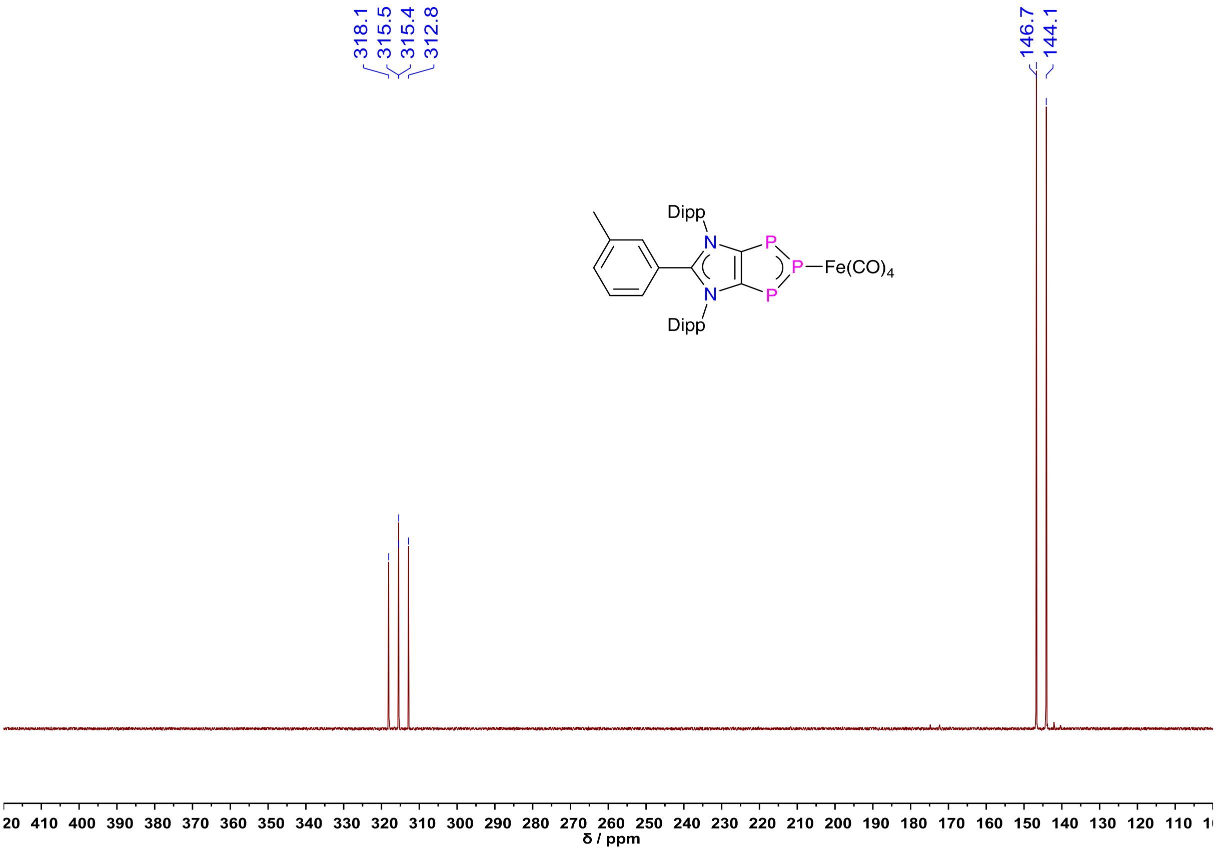

Fig. S24 ^{31}P{^{1}H} NMR (202 MHz, CD_2Cl_2, 298 K) spectrum of compound **5b**.

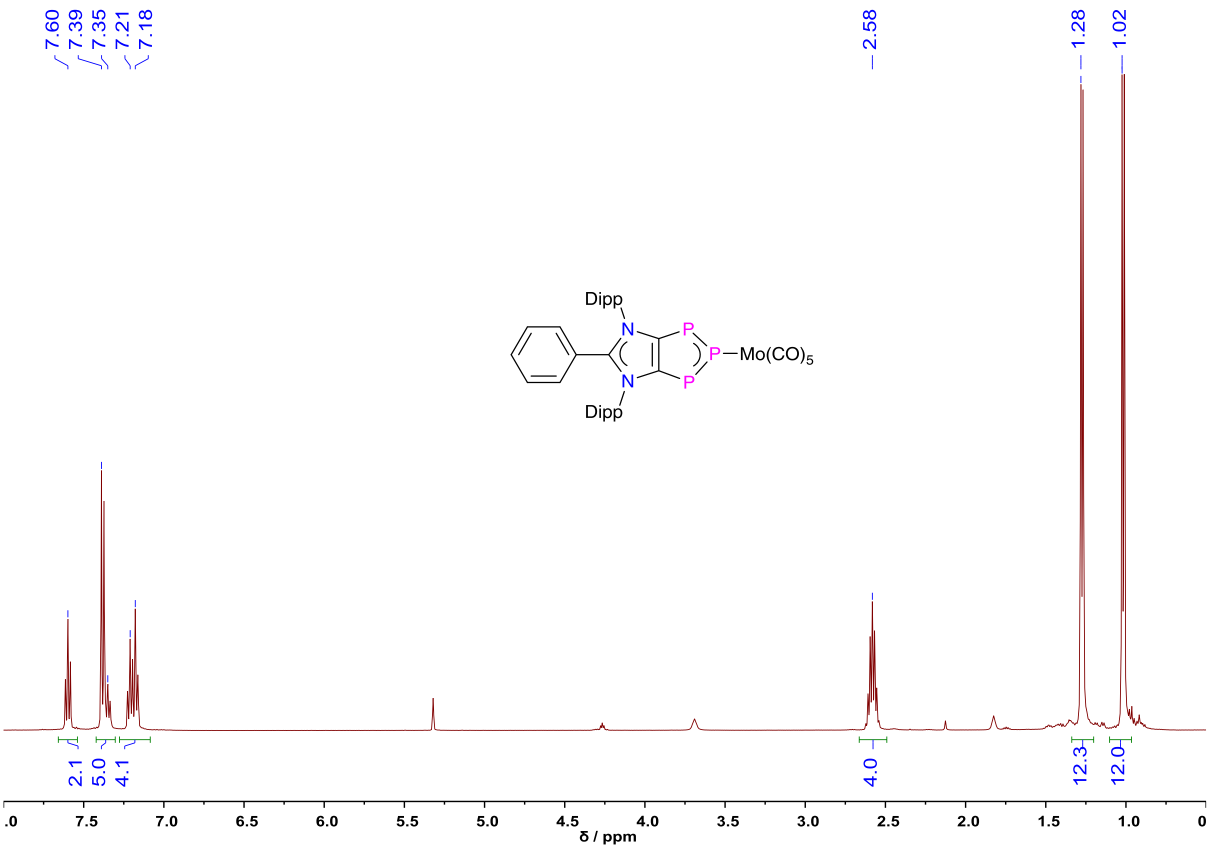

Fig. S25 ^{1}H NMR (500 MHz, CD_2Cl_2, 298 K) spectrum of compound **6**.

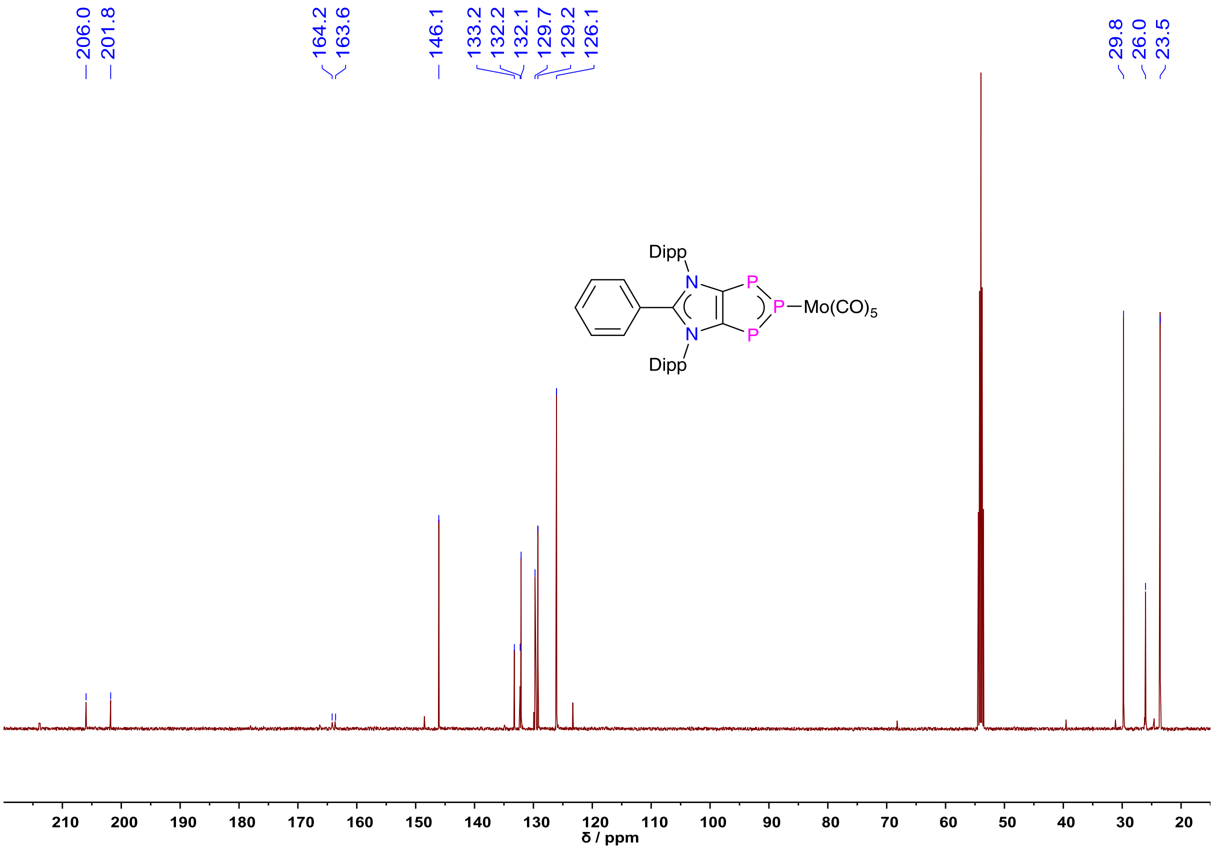

Fig. S26: ^{13}C NMR (125 MHz, CD_2Cl_2, 298 K) spectrum of compound **6**.

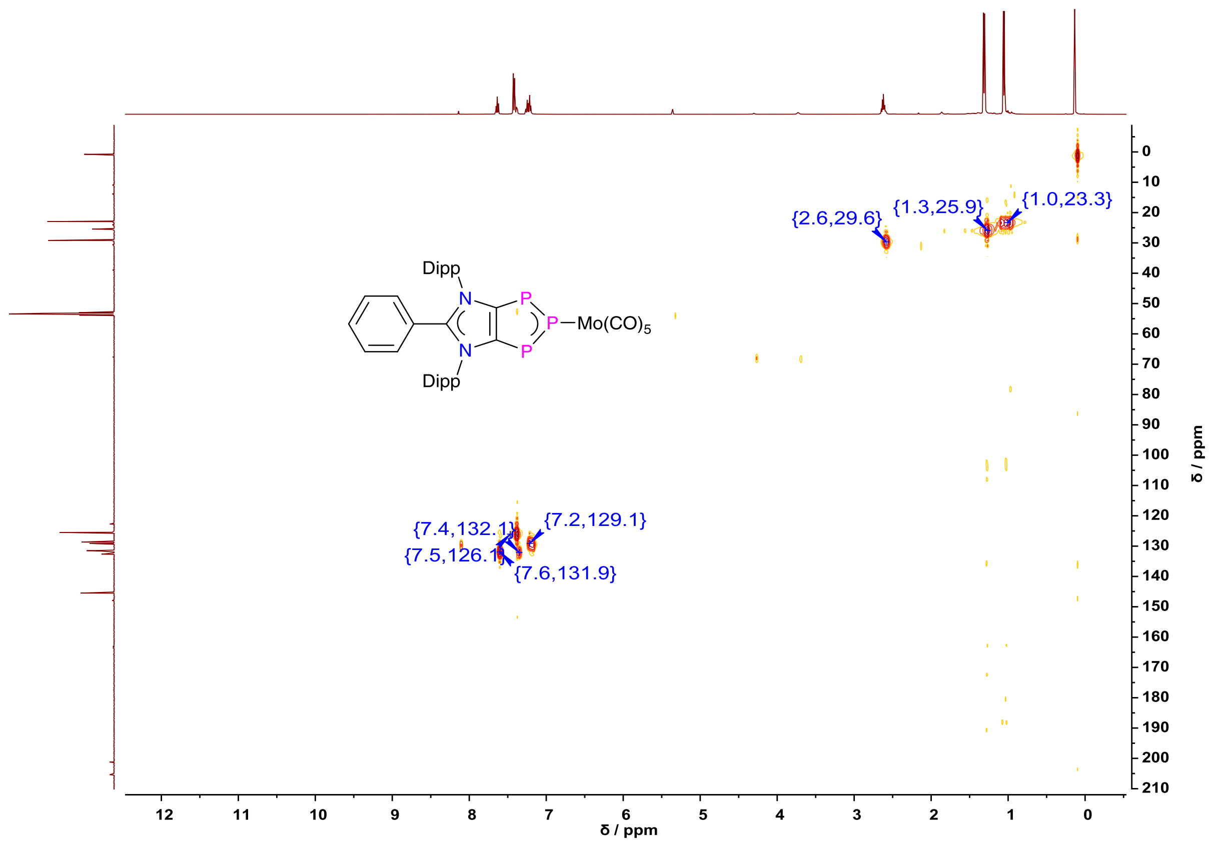

Fig. S27 ^{1}H-^{13}C-HMQC (500 MHz, CD_2Cl_2, 298 K) spectrum of compound **6**.

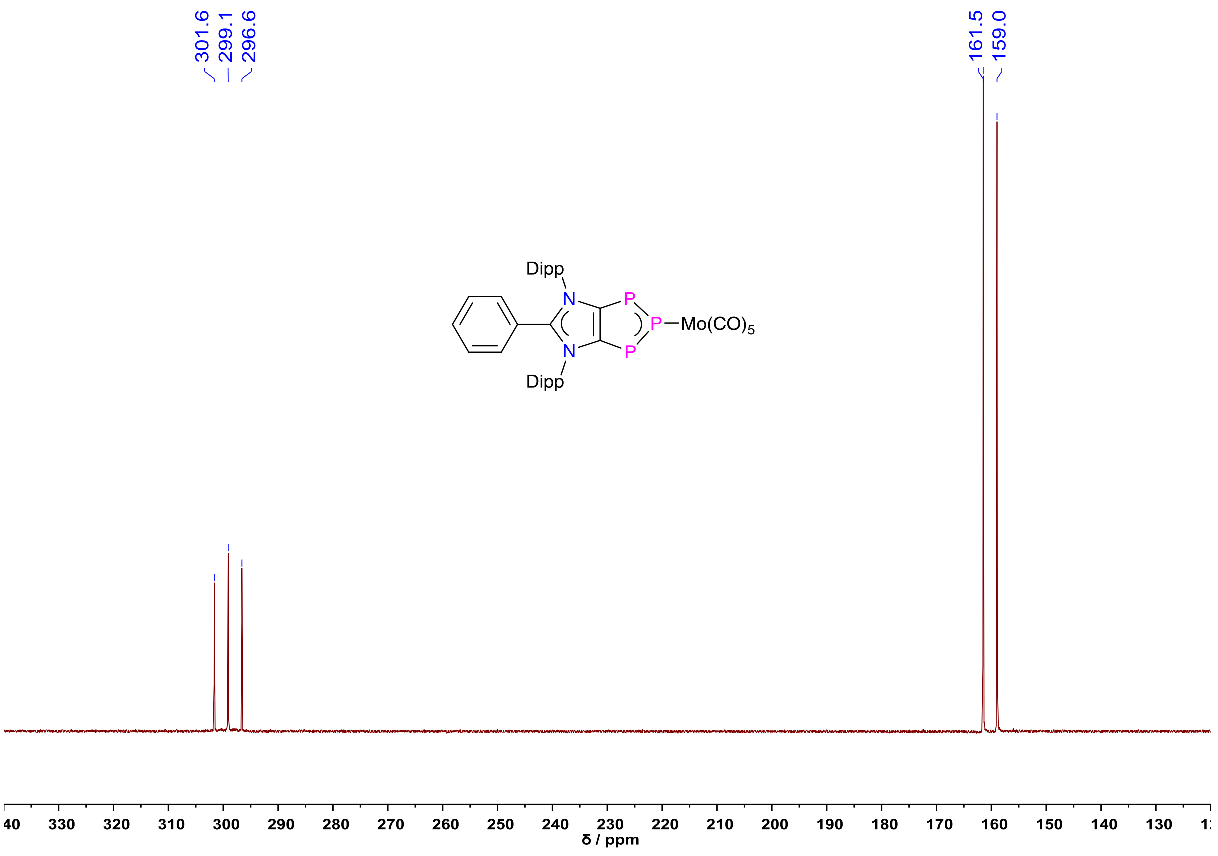

Fig. S28 $^{31}P\{^{1}H\}$ NMR (202 MHz, CD_2Cl_2, 298 K) spectrum of compound **6**.

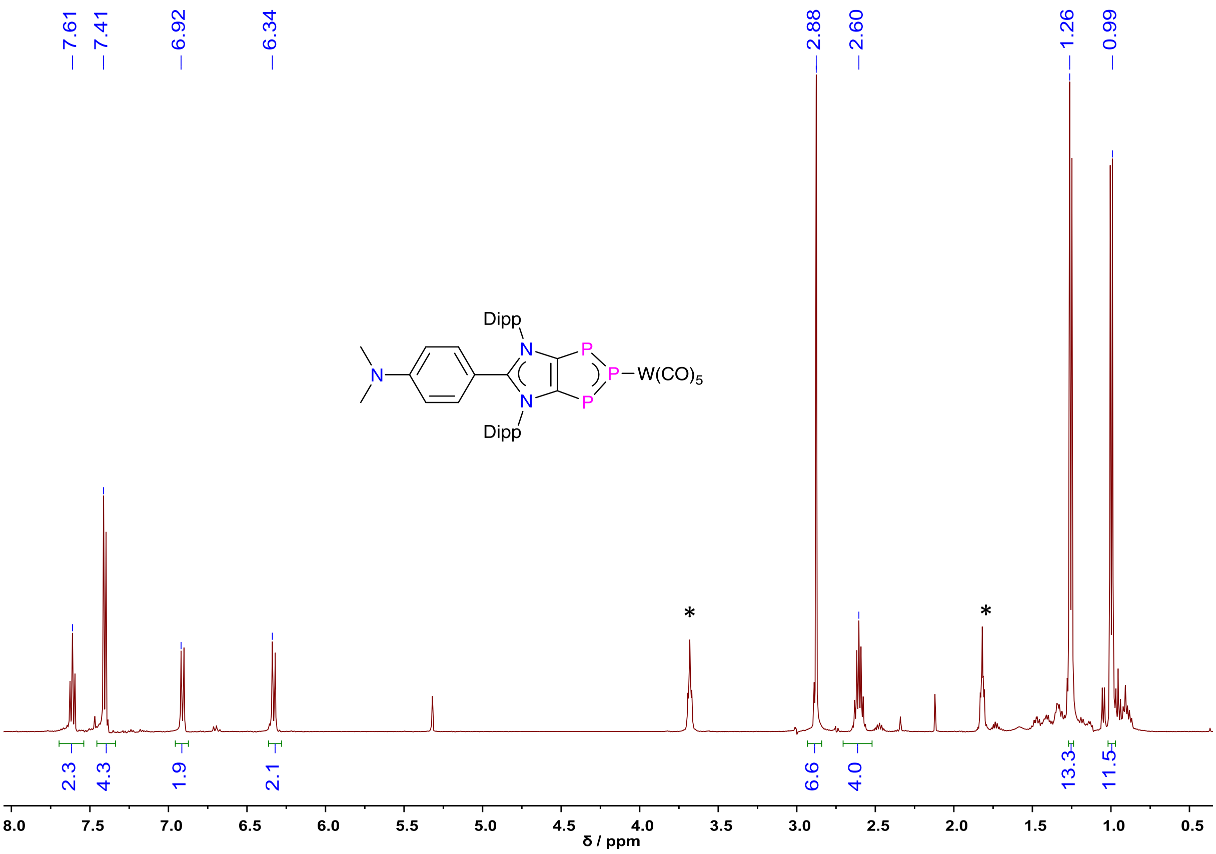

Fig. S29 ^{1}H NMR (500 MHz, CD_2Cl_2, 298 K) spectrum of compound **7**. * = THF.

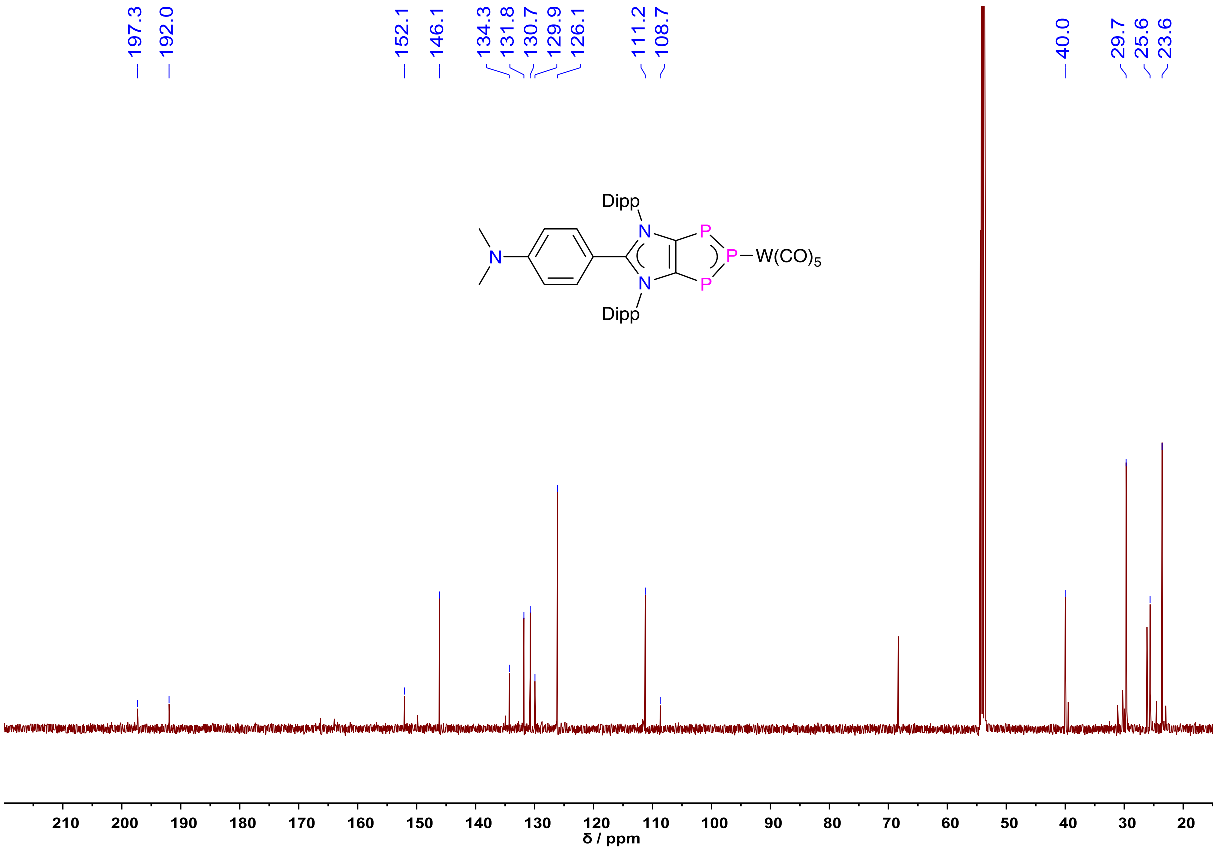

Fig. S30 ^{13}C NMR (125 MHz , CD_2Cl_2, 298 K) spectrum of compound **7**.

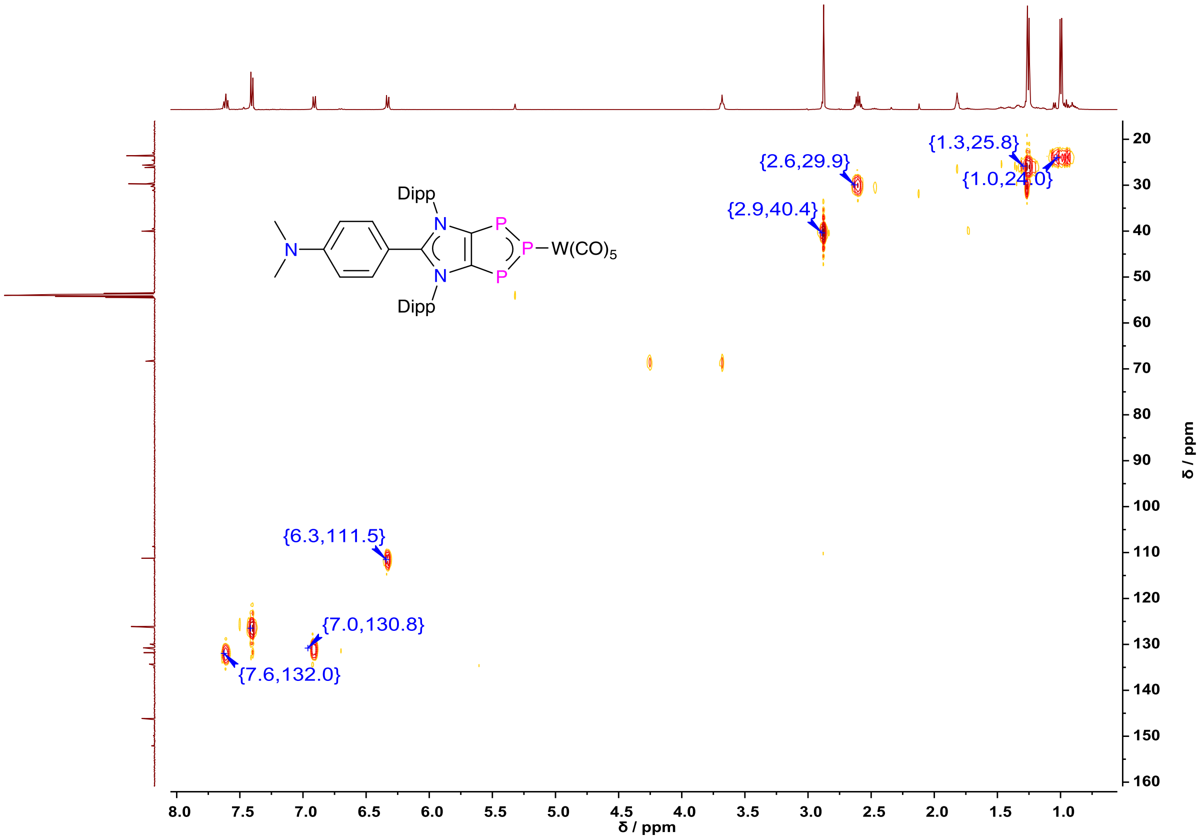

Fig. S31 ^{1}H-^{13}C-HMQC (500 MHz, CD_2Cl_2, 298 K) spectrum of compound **7**.

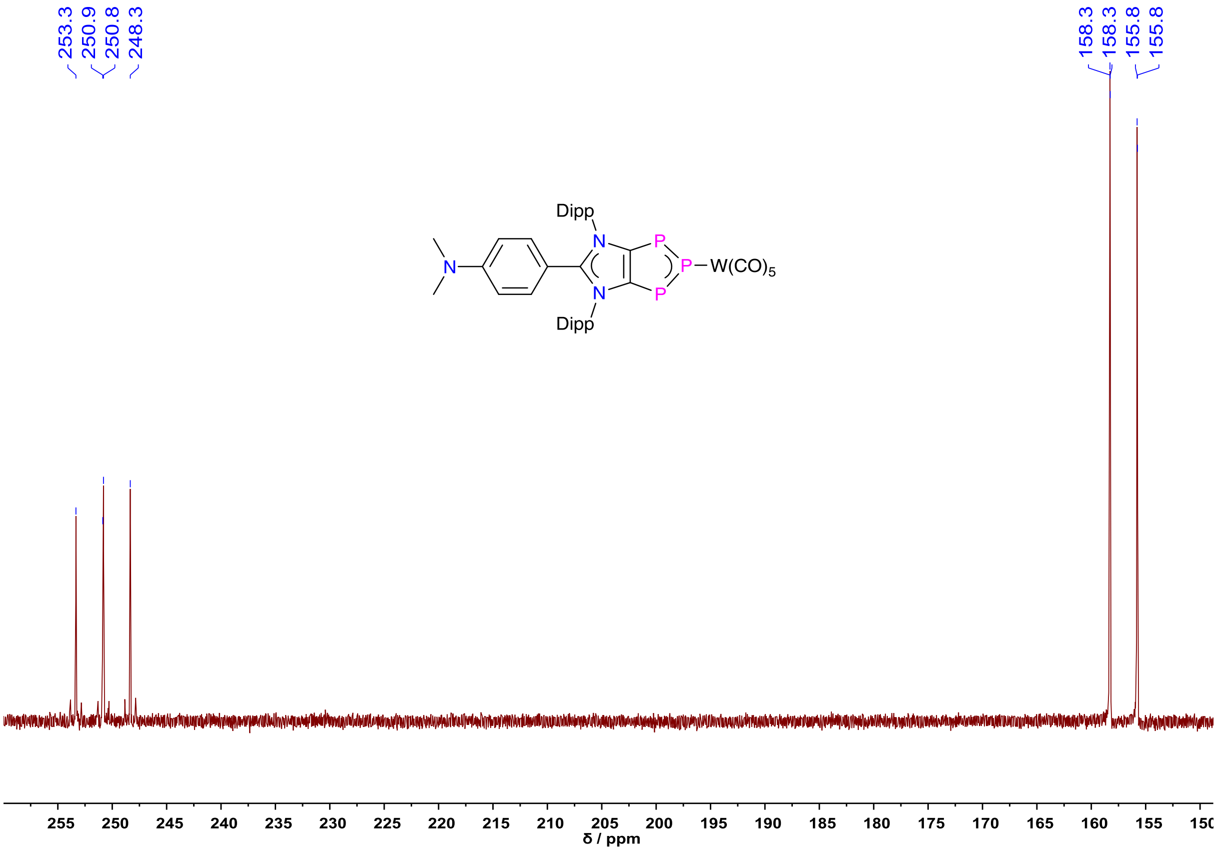

Fig. S32 ^{31}P{^{1}H} NMR (202 MHz, CD_2Cl_2, 298 K) spectrum of compound **7**.

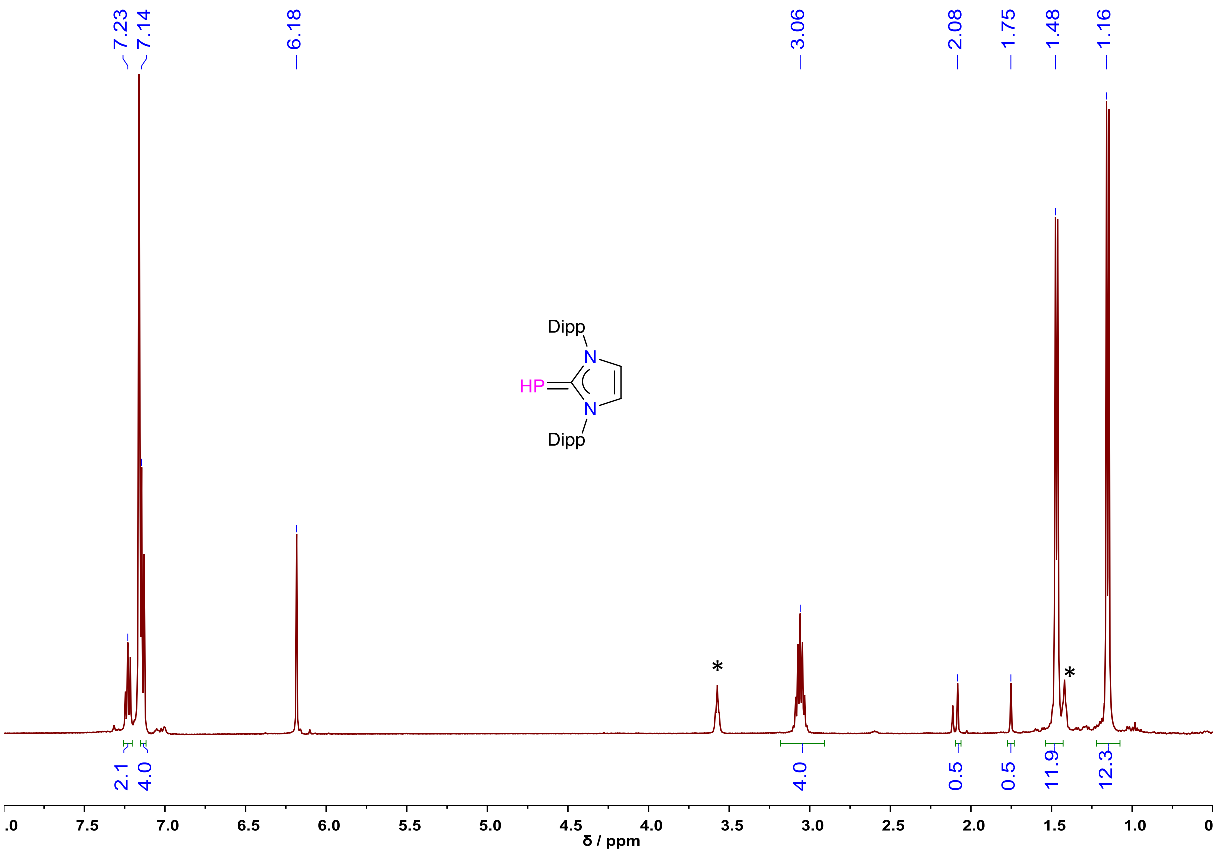

Fig. S33 ^{1}H NMR (500 MHz, C_6D_6, 298 K) spectrum of (IPr)PH prepared from (IPr)HCl and Li_3P_7. * = THF

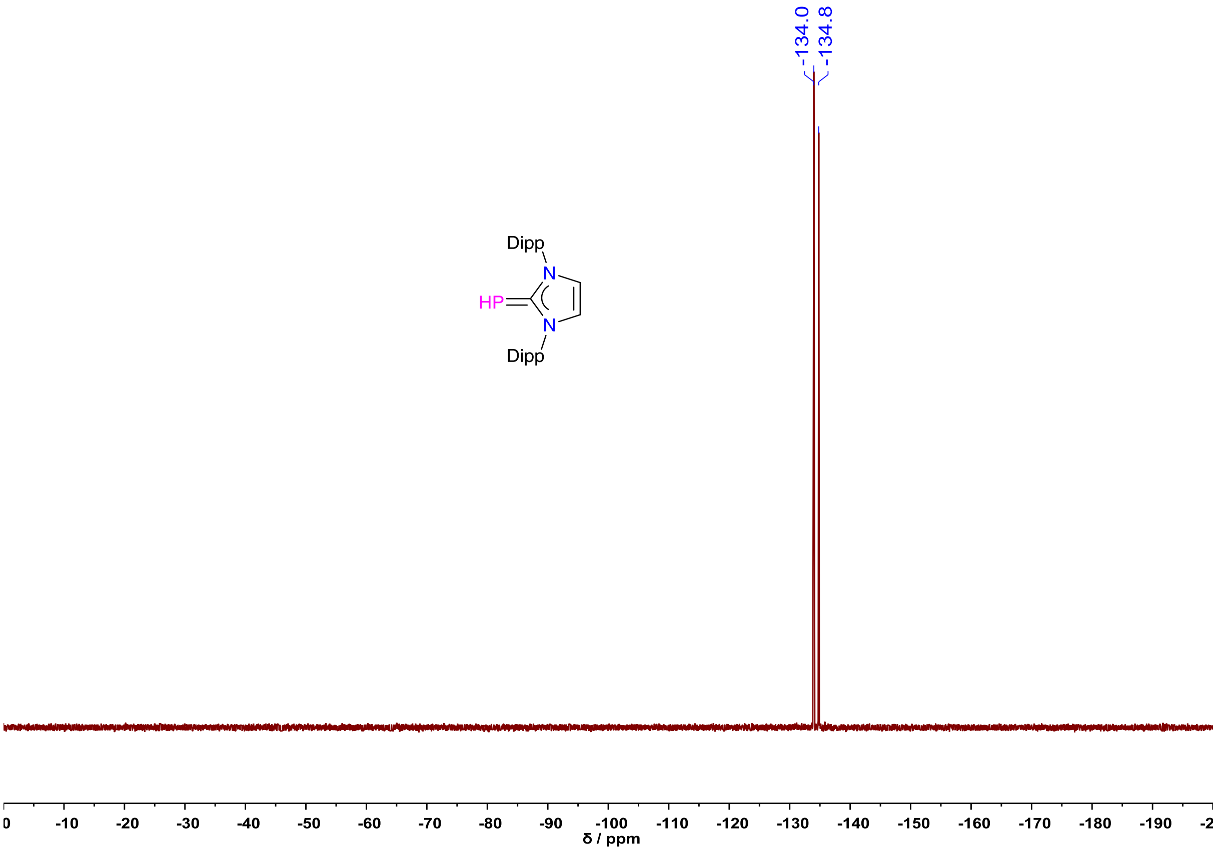

Fig. S34 ^{31}P NMR (202 MHz, C_6D_6, 298 K) of (IPr)PH prepared from (IPr)HCl and Li_3P_7.

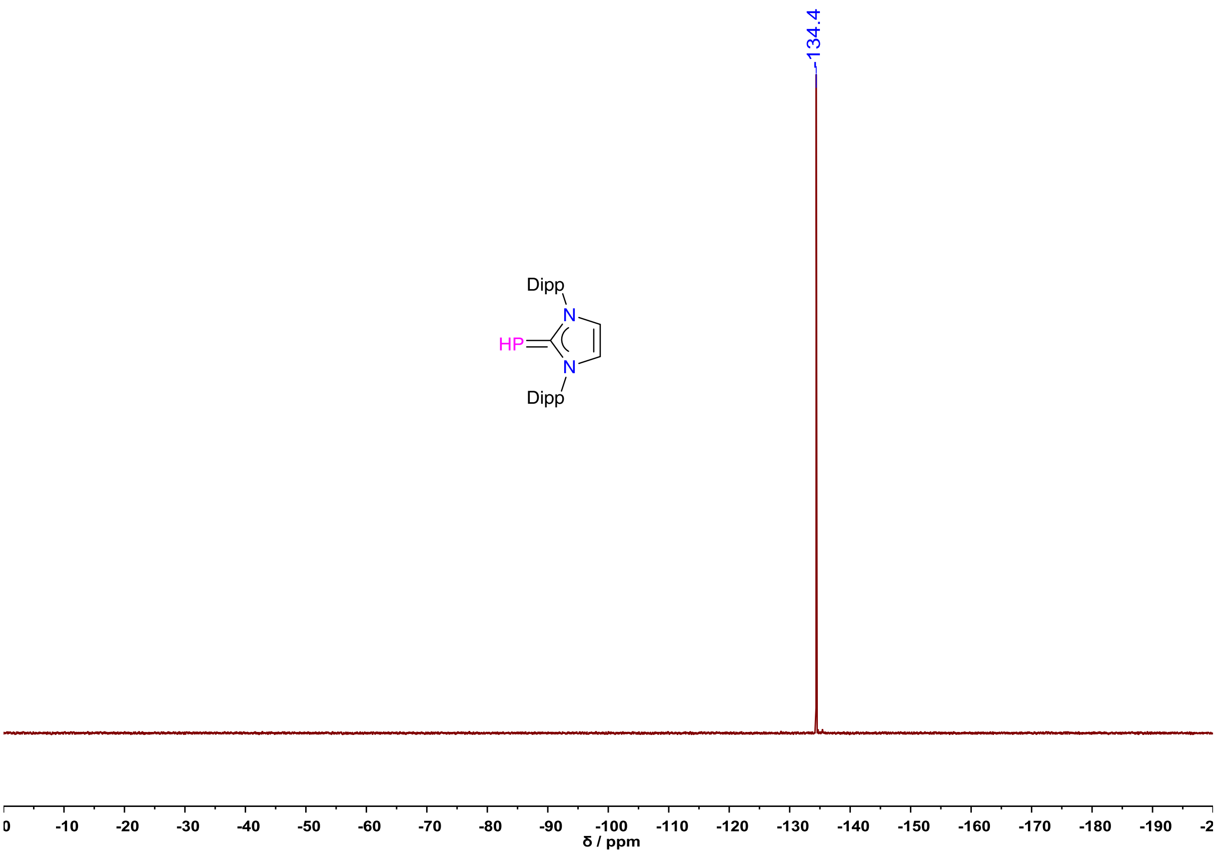

Fig. S35 $^{31}P\{^{1}H\}$ NMR (202 MHz, C_6D_6, 298 K) spectrum of (IPr)PH prepared from (IPr)HCl and Li_3P_7.

UV-vis spectroscopy

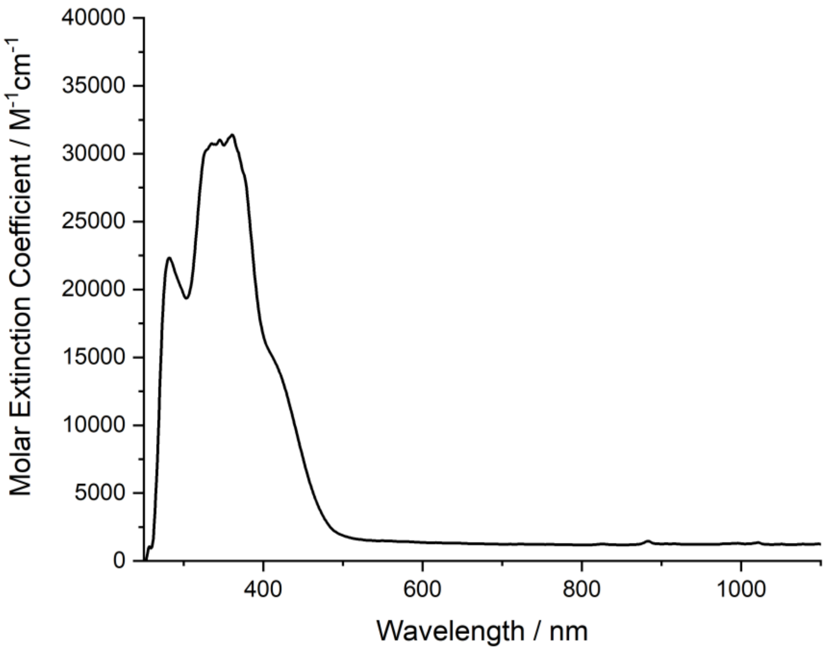

Fig. S36 UV-vis spectrum of compound **4a** ($1 \cdot 10^{-4}$ M solution in CH_2Cl_2).

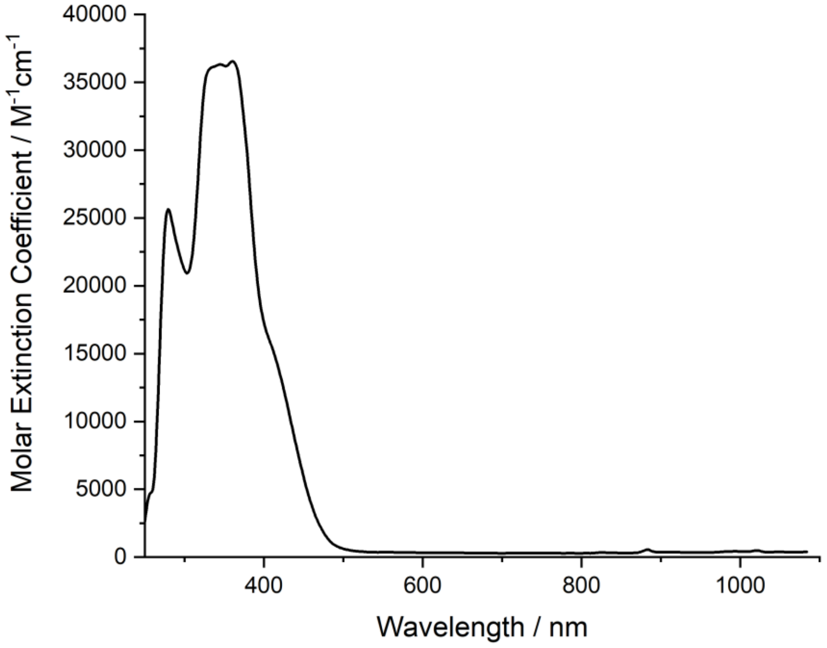

Fig. S37 UV-vis spectrum of compound **4b** ($1 \cdot 10^{-4}$ M solution in CH_2Cl_2).

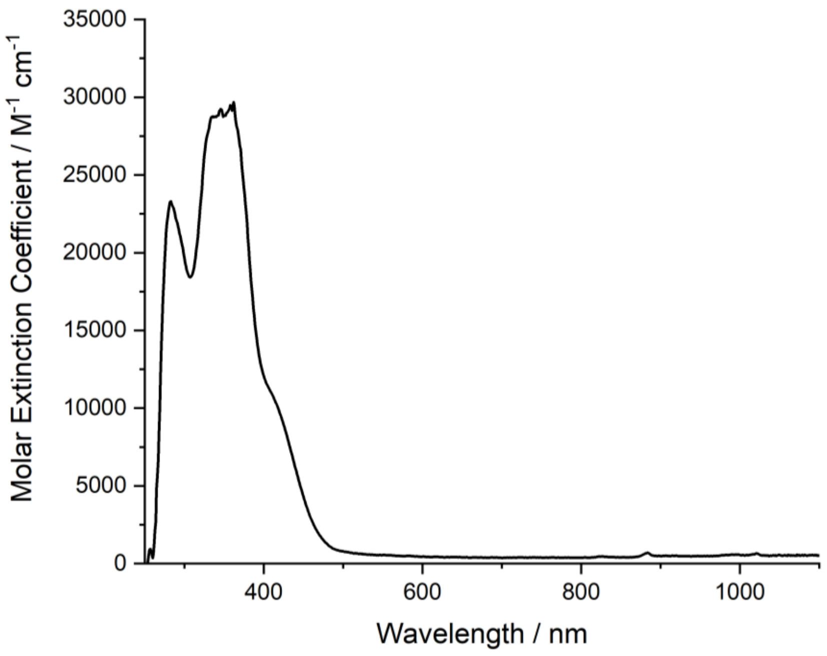

Fig. S38 UV-vis spectrum of compound **4c** ($1{\cdot}10^{-4}$ M solution in CH_2Cl_2).

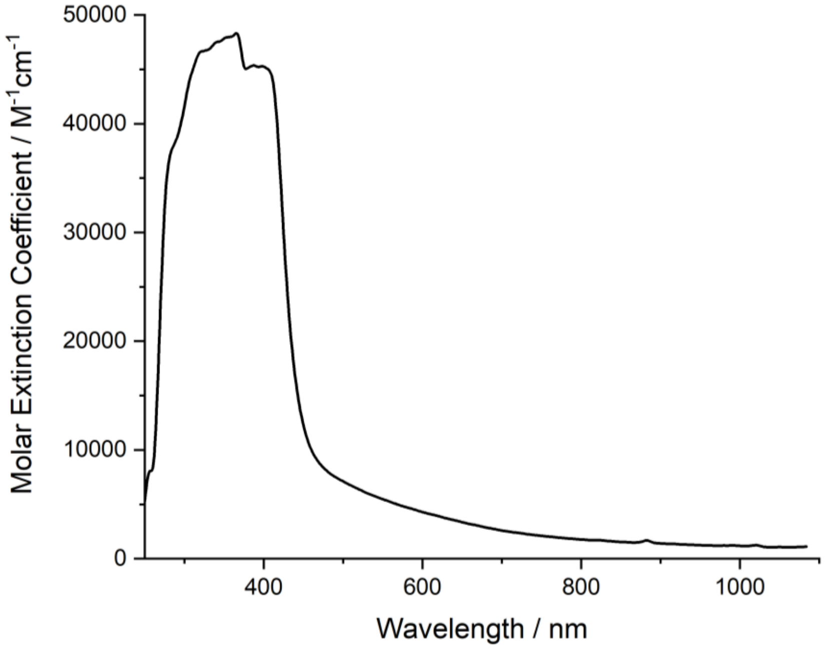

Fig. S39 UV-vis spectrum of compound **4d** ($1{\cdot}10^{-4}$ M solution in CH_2Cl_2).

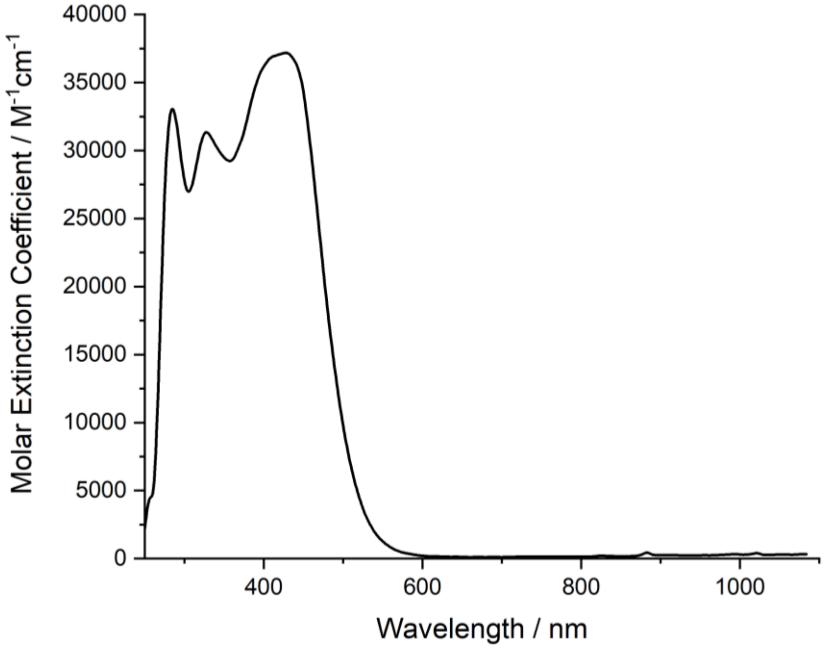

Fig. S40 UV-vis spectrum of compound **5a** ($1\cdot10^{-4}$ M solution in CH_2Cl_2).

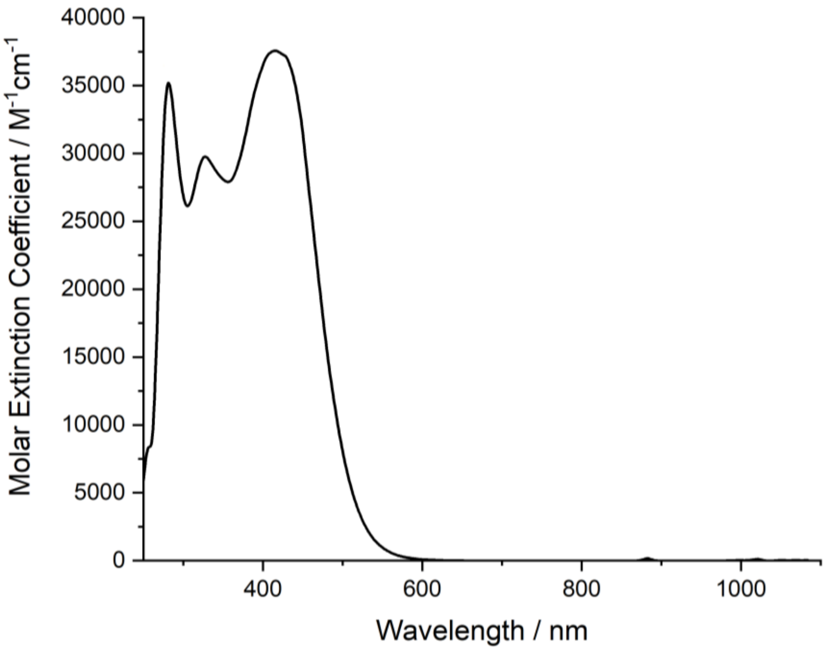

Fig. S41 UV-vis spectrum of compound **5b** ($1\cdot10^{-4}$ M solution in CH_2Cl_2).

IR spectroscopy

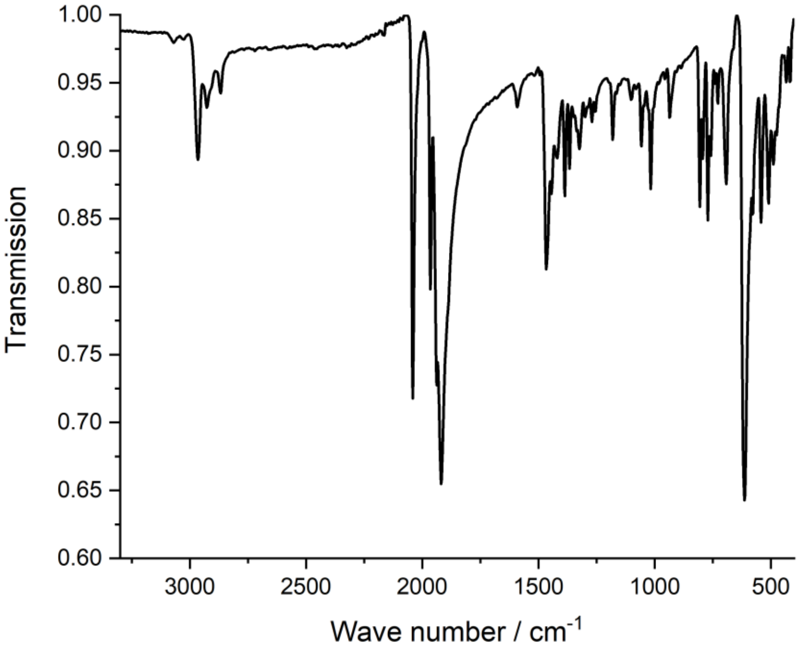

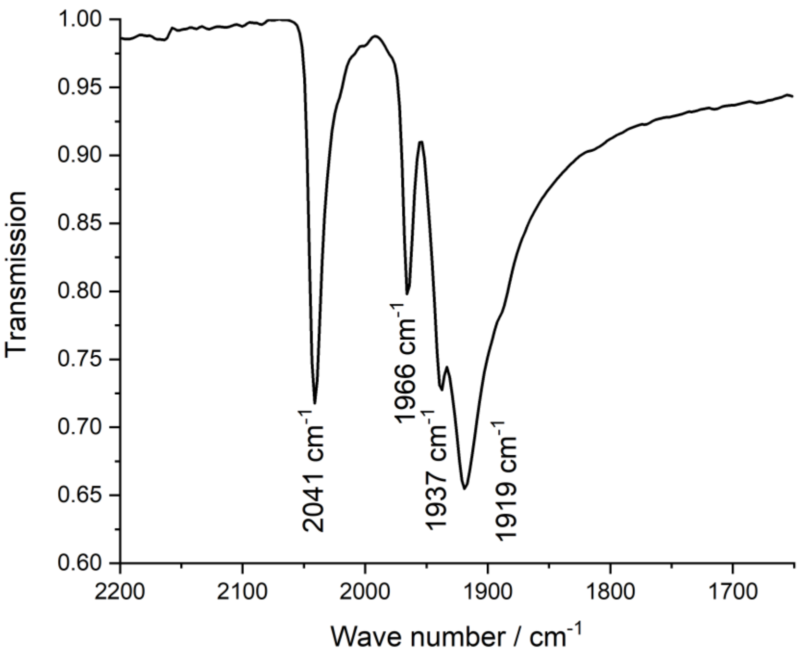

Fig. S42 IR spectrum of compound **5a** (top). Enlarged from the 2200–1650 cm^{-1} region (bottom).

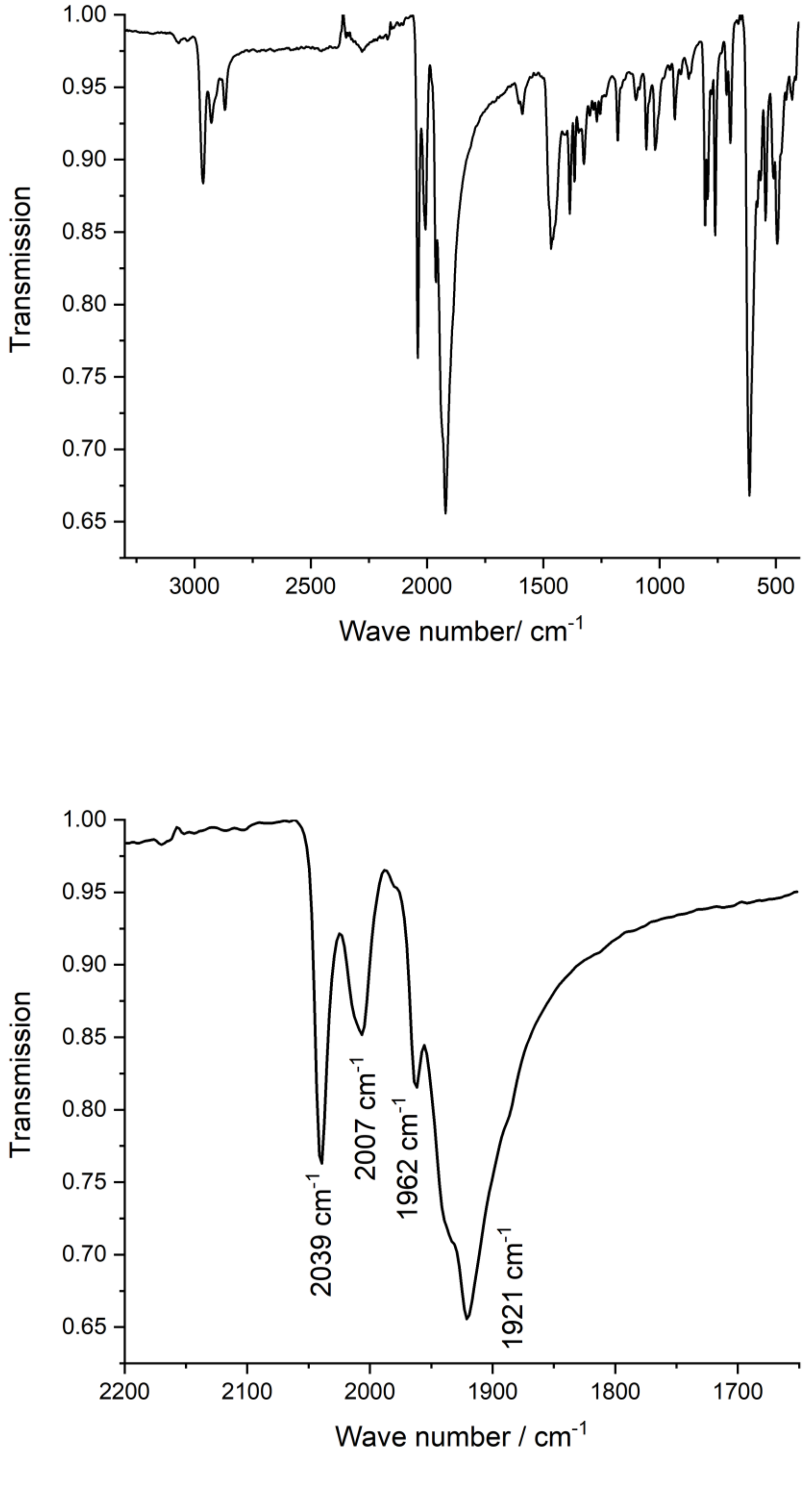

Fig. S43 IR spectrum of compound **5b** (top). Enlarged from the 2200–1650 cm^{-1} region (bottom).

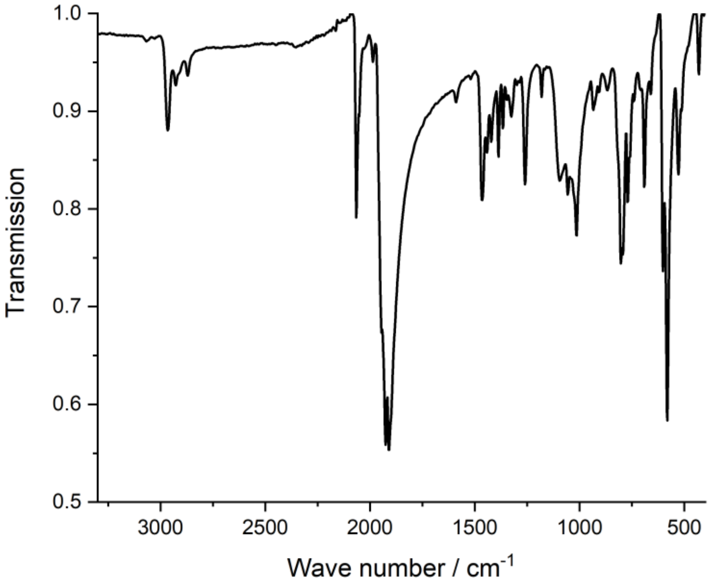

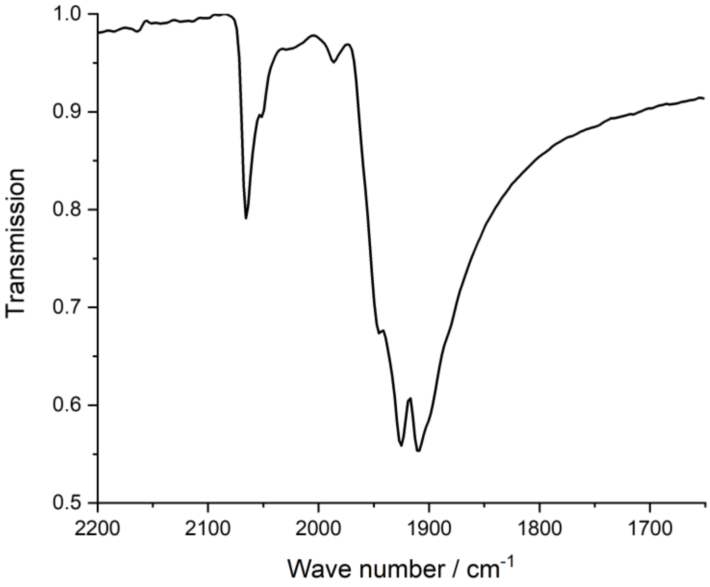

Fig. S44 IR spectrum of compound **6** (top). Enlarged from the 2200–1650 cm^{-1} region (bottom).

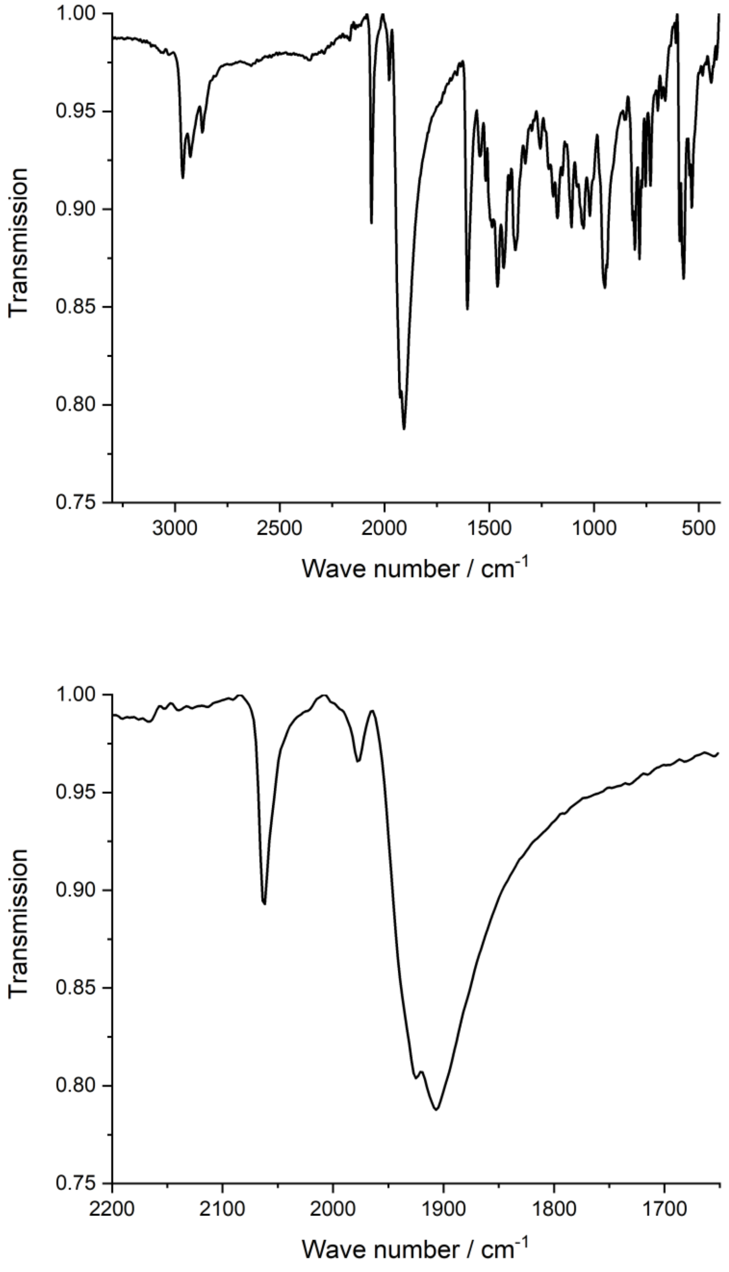

Fig. S45 IR spectrum of compound **7** (top). Enlarged from the 2200–1650 cm^{-1} region (bottom).

X-Ray diffraction studies

Single crystals of **1b**, **4a-4c**, **5a**, **5b**, **6**, and **7** were examined on a Rigaku Supernova diffractometer. The crystals were kept at 100.0(1) K during data collection. Using Olex2,[1] the structures were solved with the ShelXT[2] structure solution program using Intrinsic Phasing and refined with the ShelXL[3] refinement package using Least Squares minimization. In the structure of **4b**, the tolyl group is disordered on a twofold axis (50:50) leading to influenced disorder of methyl groups C10 and C14 with the same occupation. In the structure of **4c**, one solvent toluene molecule is disordered with ratio 58:42. In the structure of **5a**, one solvent toluene molecule is disordered over two sites (85:15). In **7** one dimethylaniline ligand (C29, C30, C32, C33, C34, and C35) is disordered over two sites with ratio 52:48 and one solvent toluene is disordered on a center of inversion.

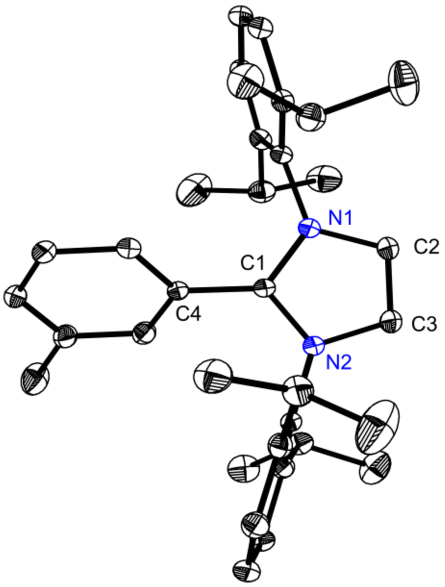

Fig. S46 Solid-state molecular structures of **1b**. Hydrogen atoms and the bromide counter anion are omitted for clarity. Selected bond lengths (Å) and angles (°): C1–N1 1.345(2), C1–N2 1.348(2), C2–N1 1.386(2), C3–N2 1.384(2), C1–C4 1.466(2), C2–C3 1.351(2), N1–C1–N2 107.0(1), N1–C2–C3 107.2(1), N2–C3–C2 107.0(1).

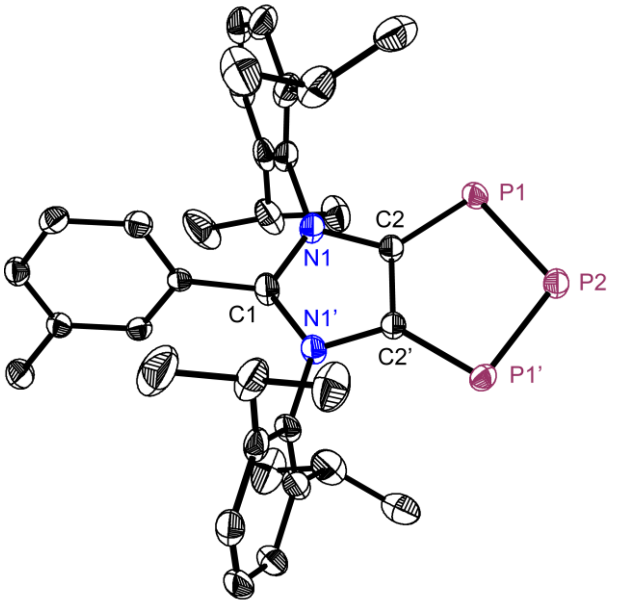

Fig. S47 Solid-state molecular structure of **4b**. Hydrogen atoms are omitted for clarity.

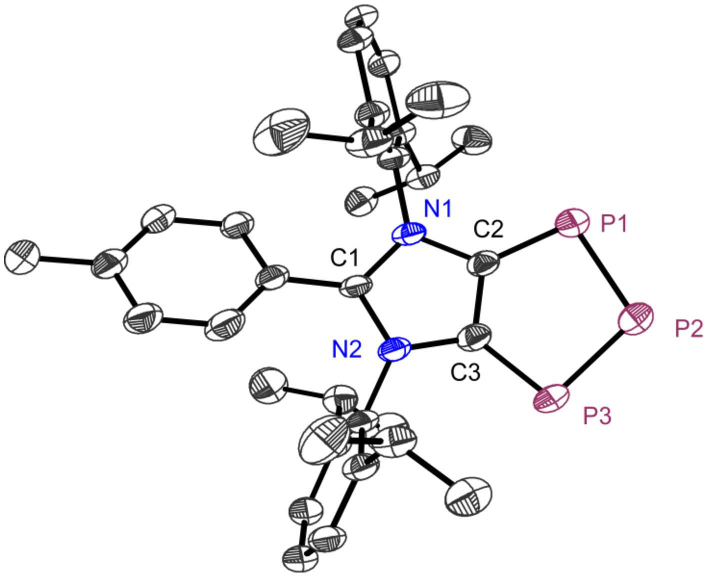

Fig. S48 Solid-state molecular structure of **4c**. Hydrogen atoms and one solvent toluene molecule are omitted for clarity.

Table S1. Selected experimental and calculated [at the M06-2X/def2SVP level of theory] bond lengths (Å) and angles (°) of compounds **4a**, **4b**, and **4c**.

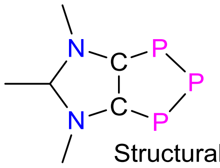<br>Structural motif	**4a**	**4b**	**4c**
C–C	1.395(5)<br>[1.402]	1.397(2)<br>[1.402]	1.395(5)<br>[1.402]
N–C	1.404(3)<br>[1.399]	1.406(1)<br>[1.399]	1.410(5), 1.406(5)<br>[1.399]
P–C	1.757(3)<br>[1.764]	1.752(1)<br>[1.764]	1.747(4), 1.755(4)<br>[1.764]
P–P	2.103(1)<br>[2.112]	2.103(1)<br>[2.112]	2.108(2), 2.107(2)<br>[2.112]
P–P–C	94.9(1)<br>[94.8]	95.1(1)<br>[94.9]	95.4(1), 95.3(2)<br>[94.9]
P–P–P	104.0(1)<br>[104.1]	103.7(1)<br>[104.0]	103.3(1)<br>[104.0]

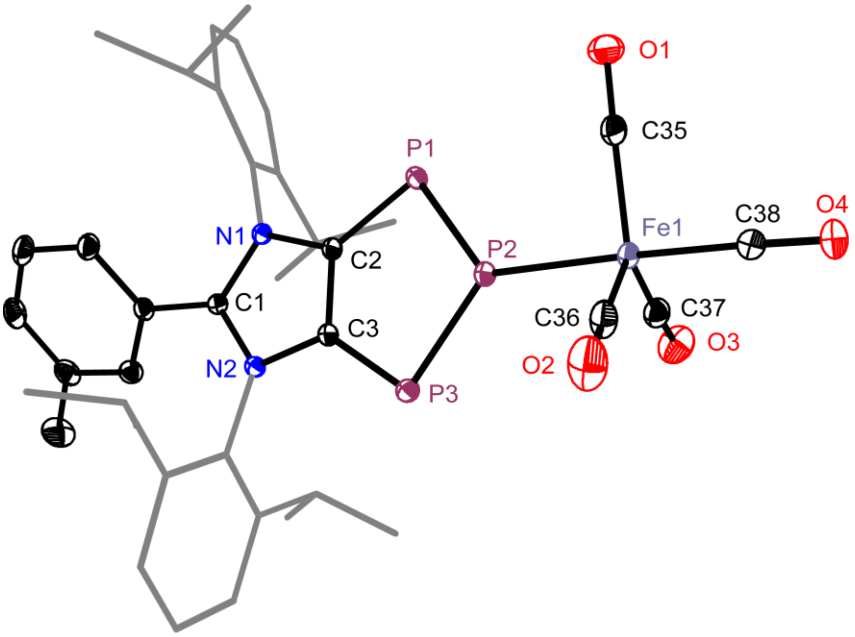

Fig. S49 Solid-state molecular structure of **5b**. Hydrogen atoms and one solvent toluene molecule are omitted for clarity. Selected bond lengths (Å) and angles (°): C–C 1.395(1), C2–N1 1.399(1), C3–N2 1.405(1), C2–P1 1.756(1), C3–P3 1.763(1), P1–P2 2.081(1), P2–P3 2.092(1), P2–Fe1 2.247(1), Fe1–C35 1.791(1), Fe1–C36 1.792(1), Fe1–C37 1.808(1), Fe1–C38 1.784(1), P1–P2–P3 108.7(1), P2–Fe1–C38 177.9(1).

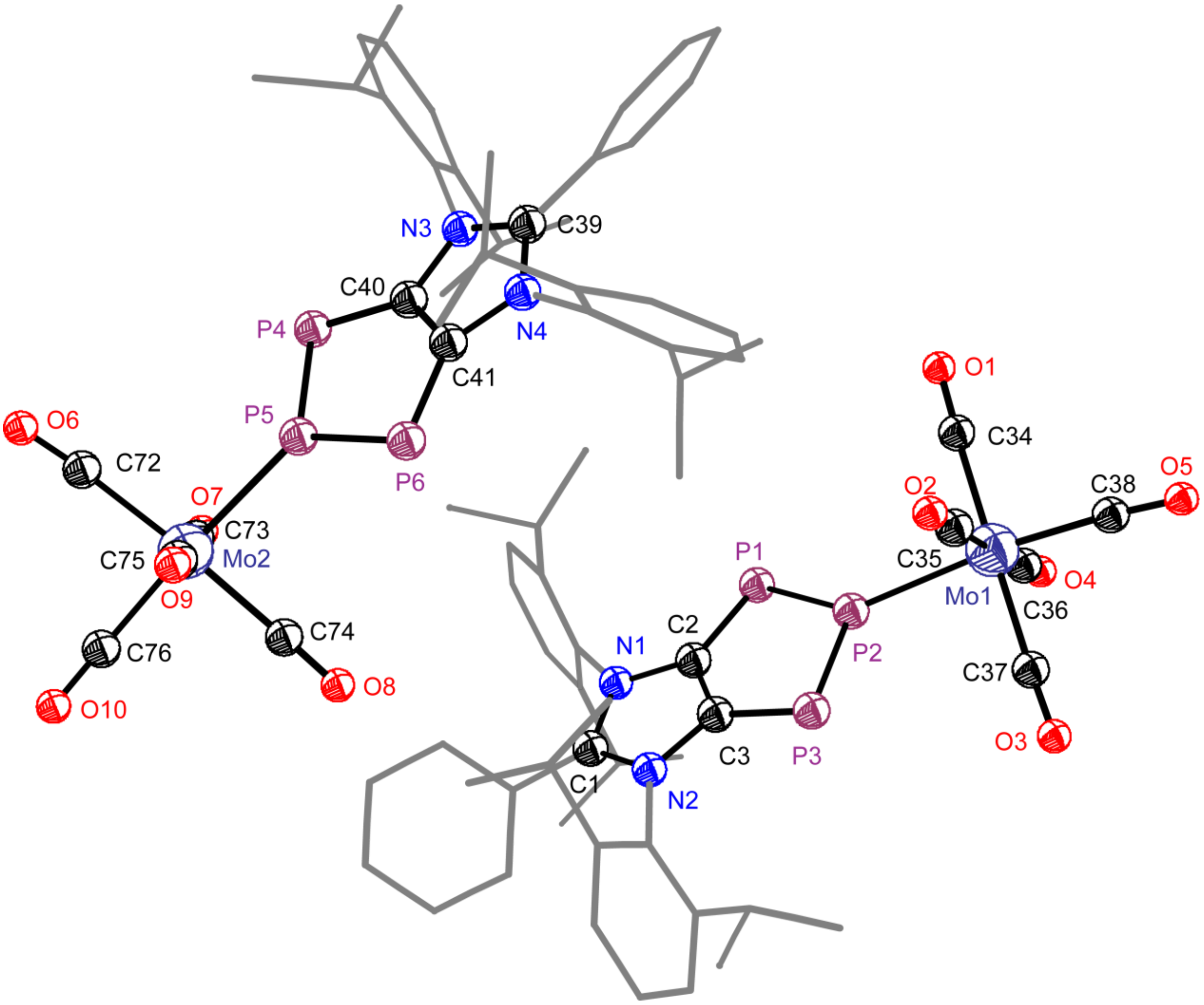

Fig. S50 Molecular Structure of **6**. Hydrogen atoms were omitted for clarity. Selected bond (Å) lengths and angles (°): N1–C2 1.405(1), N2–C3 1.397(1), C2–C3 1.396(2), C2–P1 1.763(1), C3–P3 1.754(1), P1–P2 2.091(1), P2–P3 2.085(1), P2–Mo1 2.508(1) [2.515(1)], Mo1–C34 2.056(1), Mo1–C35 2.039(1), Mo1–C36 2.047(1), Mo1–C37 2.056(1), Mo1–C38 1.995(1), N1–C2–C3 106.0(1), N2–C3–C2 106.5(1), C2–C3–P3 124.2(1), C3–C2–P1 123.6(1), P1–P2–P3 107.0(1), P1–P2–Mo1 135.4(1), P3–P2–Mo1 117.5(1), P2–Mo1–C34 95.0(1), P2–Mo1–C35 86.4(1), P2–Mo1–C36 92.8(1), P2–Mo1–C37 86.1(1), P2–Mo1–C38 171.5(1); N3–C40 1.402(1), N4–C41 1.409(1), C40–P4 1.762(1), C41–P6 1.761(1), P4–P5 2.090(1), P5–P6 2.099(1), P5–Mo2 2.515(1), Mo2–C72 2.048(1), Mo2–C73 2.051(1), Mo2–C74 2.040(1), Mo2–C75 2.048(1), Mo2–C76 1.999(1), N3–C40–C41 106.5(1), N4–C41–C40 106.1(1), C40–C41–P6 123.2(1), C41–C40–P4 124.9(1), P4–P5–P6 107.3(1), P4–P5–Mo2 128.1(1), P6–P5–Mo2 124.4(1), P5–Mo2–C72 95.6(1), P5–Mo2–C73 90.1(1), P5–Mo2–C74 87.7(1), P5–Mo2–C75 87.4(1), P5–Mo2–C76 174.6(1).

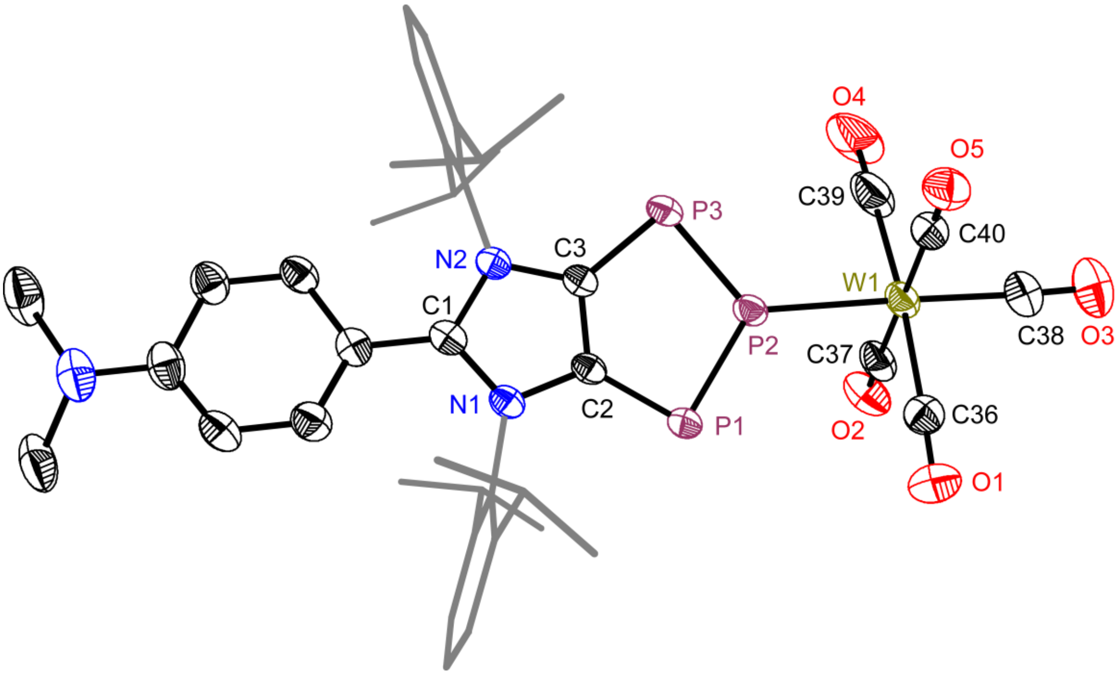

Fig. S51 Molecular Structure of **7**. Hydrogen atoms and two solvent toluene molecules were omitted for clarity. Selected bond (Å) lengths and angles (°): N1–C2 1.399(4), N2–C3 1.403(4), C2–C3 1.388(4), C2–P1 1.755(3), C3–P3 1.761(3), P1–P2 2.093(1), P2–P3 2.092(1), P2–W1 2.494(1), W1–C36 2.044(4), W1–C37 2.047(5), W1–C38 1.966(4), W1–C39 2.044(5), W1–C40 2.036(5), N1–C2–C3 106.2(3), N2–C3–C2 106.4(3), C2–C3–P3 124.0(3), C3–C2–P1 124.7(3), P1–P2–P3 107.5(1), P1–P2–W1 125.1(1), P3–P2–W1 107.5(1), P2–W1–C36 87.7(1), P2–W1–C37 91.9(1), P2–W1–C38 177.7(1), P2–W1–C39 88.3(2), P2–W1–C40 88.1(1).

Table S2. Crystallographic details of **1b** and **4a**.

	**1b**	**4a**
Empirical formula	$C_{34}H_{43}BrN_2$	$C_{33}H_{39}N_2P_3$
Formula weight	559.61	556.57
Crystal system	monoclinic	monoclinic
Space group	$P2_1/c$	C2/c
a/Å	10.3786(2)	10.5947(8)
b/Å	15.7209(3)	16.2615(8)
c/Å	19.3197(3)	17.8991(13)
β/°	100.6678(18)	97.414(7)
Volume/$Å^3$	3097.75(10)	3058.0(4)
Z	4	4
ρ_{calc} / g/cm^3	1.200	1.209
μ/mm^{-1}	1.350	1.959
F(000)	1184.0	1184.0
Crystal size / mm^3	0.27 × 0.19 × 0.07	0.17 × 0.03 × 0.02
Radiation	MoKα (λ = 0.71073)	CuKα (λ = 1.54184)
2Θ range for data collection	3.364 to 64.324	9.966 to 153.092
Index ranges	$-15 \leq h \leq 15$<br>$-22 \leq k \leq 22$<br>$-28 \leq l \leq 28$	$-12 \leq h \leq 13$<br>$-20 \leq k \leq 20$<br>$-22 \leq l \leq 22$
Reflections collected	76135	5324
Independent reflections	10359 [R_{int} = 0.0379, R_{sigma} = 0.0268]	5324 [R_{int} = 0.0747, R_{sigma} = 0.0869]
Reflections with I > 2σ(I)	8477	3981
Completeness up to Θ full	0.999	1.000
Data/restraints/parameters	10359/0/342	5324/0/179
Goodness-of-fit on F2	1.043	1.068
Final R indexes [I > 2σ(I)]	R_1 = 0.0357, wR_2 = 0.0815	R_1 = 0.0437, wR_2 = 0.1184
Final R indexes [all data]	R_1 = 0.0510, wR_2 = 0.0874	R_1 = 0.0610, wR_2 = 0.1298
Largest diff. peak/hole / e Å-3	0.96/-0.68	0.32/-0.31
CCDC number	1939608	1939609

Table S3. Crystallographic details of **4b** and **4c**.

	**4b**	**4c**(toluene)
Empirical formula	$C_{34}H_{41}N_2P_3$	$C_{41}H_{49}N_2P_3$
Formula weight	570.60	662.73
Crystal system	monoclinic	orthorhombic
Space group	C2/c	$P2_12_12_1$
a/Å	10.8550(3)	11.1499(5)
b/Å	15.5962(3)	17.6614(8)
c/Å	19.1059(4)	19.2232(6)
β/°	102.358(2)	90
Volume/Å^3	3159.62(13)	3785.5(3)
Z	4	4
ρ_{calc} / g/cm^3	1.200	1.163
μ/mm-1	0.213	1.659
F(000)	1216.0	1416.0
Crystal size/mm^3	0.29 x 0.21 x 0.11	0.15 × 0.11 × 0.09
Radiation	MoKα (λ = 0.71073)	CuKα (λ = 1.54184)
2Θ range for data collection	5.224 to 65.648	6.796 to 153.536
Index ranges	$-16 \leq h \leq 16$<br>$-23 \leq k \leq 23$<br>$-29 \leq l \leq 28$	$-13 \leq h \leq 13$<br>$-21 \leq k \leq 19$<br>$-23 \leq l \leq 24$
Reflections collected	70658	31369
Independent reflections	5715 [R_{int} = 0.0394, R_{sigma} = 0.0210]	7770 [R_{int} = 0.0500, R_{sigma} = 0.0417]
Reflections with I > 2σ(I)	4716	7012
Completeness up to Θ full	0.998	0.999
Data/restraints/parameters	5715/0/234	7770/0/491
Goodness-of-fit on F2	1.070	1.037
Final R indexes [I > 2σ(I)]	R_1 = 0.0388, wR_2 = 0.0970	R_1 = 0.0509, wR_2 = 0.1214
Final R indexes [all data]	R_1 = 0.0521, wR_2 = 0.1052	R_1 = 0.0566, wR_2 = 0.1253
Largest diff. peak/hole / e Å-3	0.61/-0.26	0.29/-0.45
CCDC number	1939610	1939611

Table S4. Crystallographic details of **5a** and **5b**.

	**5a**(toluene)	**5b**(toluene)
Empirical formula	$C_{44}H_{47}FeN_2O_4P_3$	$C_{45}H_{49}FeN_2O_4P_3$
Formula weight	816.59	830.62
Crystal system	monoclinic	monoclinic
Space group	$P2_1/c$	$P2_1/c$
a/Å	10.9413(2)	10.83254(14)
b/Å	23.5902(4)	24.9423(3)
c/Å	16.2953(3)	16.03521(18)
β/°	96.991(2)	96.0125(11)
Volume / $Å^3$	4174.67(13)	4308.69(9)
Z	4	4
ρ_{calc} / g/cm^3	1.299	1.280
μ/mm^{-1}	0.520	0.505
F(000)	1712.0	1744.0
Crystal size/mm^3	0.37 × 0.27 × 0.18	0.22 × 0.15 × 0.14
Radiation	MoKα (λ = 0.71073)	MoKα (λ = 0.71073)
2Θ range for data collection	5.068 to 65.676	5.052 to 65.760
Index ranges	$-16 \le h \le 16$<br>$-35 \le k \le 35$<br>$-24 \le l \le 24$	$-16 \le h \le 16$<br>$-38 \le k \le 37$<br>$-24 \le l \le 24$
Reflections collected	127619	261252
Independent reflections	14823 [R_{int} = 0.0417, R_{sigma} = 0.0286]	15612 [R_{int} = 0.0435, R_{sigma} = 0.0200]
Reflections with I > 2σ(I)	12232	13655
Completeness up to Θ full	0.999	0.999
Data/restraints/parameters	14823/15/549	15612/0/506
Goodness-of-fit on F2	1.044	1.039
Final R indexes [I > 2σ(I)]	R_1 = 0.0344, wR_2 = 0.0756	R_1 = 0.0311, wR_2 = 0.0789
Final R indexes [all data]	R_1 = 0.0482, wR_2 = 0.0823	R_1 = 0.0386, wR_2 = 0.0827
Largest diff. peak/hole / e Å-3	0.48/-0.32	0.55/-0.41
CCDC number	1939612	1939613

Table S5. Crystallographic details of **6** and **7**.

	**6**	**7**(2 toluene)
Empirical formula	$C_{38}H_{39}MoN_2O_5P_3$	$C_{50.5}H_{56}N_3O_5P_3W$
Formula weight	792.56	1061.74
Crystal system	triclinic	triclinic
Space group	P-1	P-1
a/Å	12.68020(10)	12.0860(2)
b/Å	13.72590(10)	13.1486(2)
c/Å	23.0210(2)	16.9350(2)
α/°	93.5620(10)	106.1610(10)
β/°	101.0270(10)	98.4870(10)
γ/°	98.0390(10)	100.5390(10)
Volume/Å^3	3877.80(6)	2484.47(7)
Z	4	2
ρ_{calc} / g/cm^3	1.358	1.419
μ/mm^{-1}	0.505	5.599
F(000)	1632.0	1078.0
Crystal size/mm3	0.20 × 0.17 × 0.16	0.18 × 0.14 × 0.07
Radiation	MoKα (λ = 0.71073)	Cu Kα (λ = 1.54184)
2Θ range for data collection	5.214 to 65.692	5.558 to 152.980
Index ranges	-19 ≤ h ≤ 19<br>-19 ≤ k ≤ 20<br>-34 ≤ l ≤ 34	-15 ≤ h ≤ 15<br>-16 ≤ k ≤ 16<br>-21 ≤ l ≤ 21
Reflections collected	240247	92149
Independent reflections	27490 [R_{int} = 0.0347, R_{sigma} = 0.0216]	10372 [R_{int} = 0.0474, R_{sigma} = 0.0201]
Reflections with I > 2σ(I)	24041	9927
Completeness up to Θ max	0.999	1.000
Data/restraints/parameters	27490/0/899	10372/0/664
Goodness-of-fit on F2	1.055	1.078
Final R indexes [I > 2σ(I)]	R_1 = 0.0279, wR_2 = 0.0619	R_1 = 0.0403, wR_2 = 0.1108
Final R indexes [all data]	R_1 = 0.0356, wR_2 = 0.0652	R_1 = 0.0416, wR_2 = 0.1120
Largest diff. peak/hole / e Å-3	0.64/-0.58	3.38/-1.11
CCDC number	1939614	1939615

Computational details

All geometries were optimized with the Gaussian 16 program suite[4] using the DFT functional M06-2X[5] in combination with the Ahlrichs def2-SVP[6] basis function as implemented. The stationary points were located with the Berny algorithm using redundant internal coordinates. Analytical Hessians were computed to determine the nature of stationary points (zero imaginary frequencies for minima).[7] The electronic energies were improved by single point calculations at the M06-2X/def2-TZVPP level of theory.

The Wiberg Bond Indices (WBI)[8] and NPA[9] atomic partial charges have been calculated at the M06-2X/def2-TZVPP//M06-2X/def2-SVP level of theory using the NBO 3.1 interface of Gaussian.[10]

Plots of the anisotropy of induced current density (AICD) were done with the help of the AICD 2.0.1 program of Herges and coworkers[11] and based on M06-2X/def2-TZVPP//def2-SVP CSGT calculations with IOp(10/93=1) keyword in Gaussian 16.

Time-dependent density functional theory (Full-TDDFT) was employed to calculate excitation energies as implemented in ORCA 4.0.1.[12] We used the functional M06-2X in combination the def2-SVP basis sets. The solvent (CH_2Cl_2) was described by the conductor-like polarizable continuum model, CPCM.[13]

Table S6. Electronic energies of selected MOs of compounds **4a–4d** and **5a**, calculated at M06-2X/def2-TZVPP//M06-2X/def2-SVP level of theory.

**orbital**	energy / eV				
	**4a**	**4b**	**4c**	**4d**	**5a**
L+3	+0.06	+0.06	+0.08	+0.19	−0.18
L+2	−0.08	−0.06	−0.04	+0.10	−0.54
L+1	−0.39	−0.35	−0.36	−0.22	−0.60
L	−1.25	−1.19	−1.16	−0.82	−1.51
H	−6.08	−6.06	−6.04	−5.90	−6.42
H−1	−6.61	−6.58	−6.55	−6.31	−6.65
H−2	−7.35	−7.32	−7.31	−7.16	−6.70
H−3	−7.50	−7.48	−7.46	−7.33	−7.27
HOMO/LUMO gap	4.83	4.87	4.88	5.08	4.90

Table S7. Wiberg bond indices (WBI) and natural population analysis (NPA) atomic charges of the main parts of compounds **4a–4d**, **5a** and **1a**, calculated at M06-2X/def2-TZVPP//def2-SVP level of theory. The numbering of the atoms is in accordance with Figure F4.

**bond**	**WBI**					
	**4a**	**4b**	**4c**	**4d**	**5a**	**1a**
C1–N1/2	1.26	1.26	1.26	1.26	1.26	1.28
C2–N1/C3–N2	1.04	1.04	1.04	1.04	1.06	1.14
C2–C3	1.31	1.31	1.31	1.31	1.32	1.64
C2–P1/C3–P3	1.18	1.18	1.18	1.18	1.16	–
P1/3–P2	1.40	1.40	1.40	1.40	1.35	–
Fe–P2	–	–	–	–	0.77	–
**atom**	**NPA atomic charge**					
C1	+0.44	+0.44	+0.44	+0.46	+0.44	+0.47
N1/N2	−0.35	−0.35	−0.35	−0.36	−0.35	−0.32
C2/C3	−0.24	−0.24	−0.24	−0.24	−0.23	−0.04
P1/P3	+0.12	+0.12	+0.12	+0.12	+0.19	–
P2	−0.10	−0.10	−0.10	−0.10	+0.25	–

Table S8. Nuclear independent chemical shifts (NICS) for the C_3N_2 and C_2P_3 moieties of compounds **4a–4d** and **5a**, calculated at the M06-2X/def2-TZVPP//def2-SVP level of theory. NICS values are given for the center of the respective moiety (NICS(0)) as well as for distances of 1 (NICS(1)) and 2 Å (NICS(2)) perpendicular to the moieties. NICS values of benzene, representing a typical aromatic system, and cyclobutadiene (CBD), as a typical antiaromatic system, are given for comparison.

C_3N_2/ C_2P_3	**4a**	**4b**	**4c**	**4d**	**5a**	C_6H_6/ CBD
NICS(0)	−7.08/ −10.19	−7.29/ −10.31	−7.29/ −10.31	−6.77/ −10.37	−7.57/ −9.95	−7.53/ 33.21
NICS(1)	−5.94/ −10.18	−6.11/ −10.28	−6.11/ −10.23	−5.64/ −10.21	−6.29/ −9.58	−10.19/ 21.09
NICS(2)	−2.43/ −5.51	−2.53/ −5.21	−2.53/ −5.53	−2.36/ −5.52	−2.52/ −5.12	−5.22/ 4.98

Table S9. Wavelength (λ), oscillator strength (f) and main assignment of the TD-PCM(CH_2Cl_2)/M06-2X/def2-SVP results for compound **4a**; threshold for printing excitations was chosen to be $f \geq 0.10$.

**state no.**	**λ / nm**	***f***	**Assignment**
**2**	366.5	0.2561	H−1 → L (c = 0.8582)
**27**	235.9	0.1000	H−9 → L (c = 0.4638)
**38**	233.5	0.2152	H−2 → L+3 (c = 0.3649)
**44**	221.8	0.1289	H−2 → L+7 (c = 0.2205)
**46**	225.6	0.1858	H−6 → L+1 (c = 0.6336)
**47**	219.9	0.1170	H−2 → L+5 (c = 0.2327)
**49**	218.2	0.1450	H−2 → L+5 (c = 0.3151)

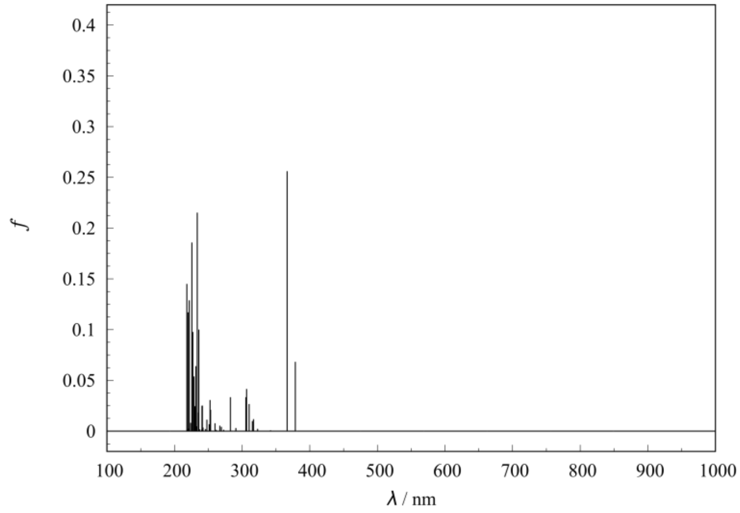

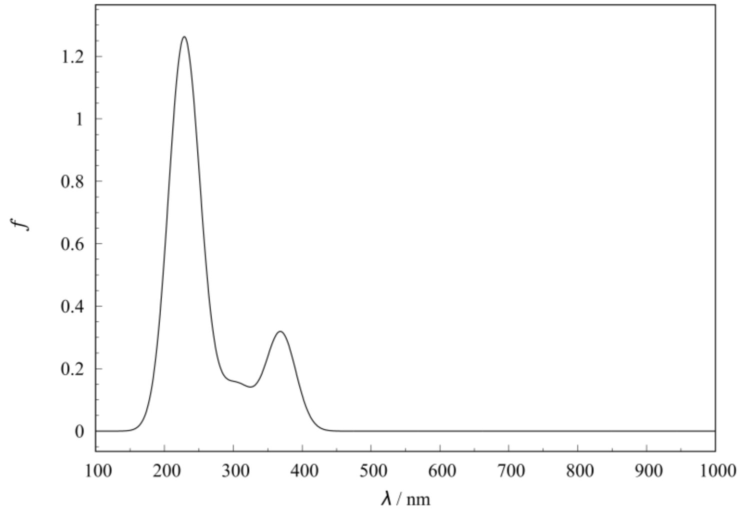

Fig. S52 UV-visible spectrum (lines: top; Gaussian line broadening with a full width at half maximum (fwhm) of 50 cm^{-1}: bottom) of **4a** calculated at TD-PCM(CH_2Cl_2)/M06-2X/def2-SVP.

Table S10. Wavelength (λ), oscillator strength (f) and main assignment of the TD-PCM(CH_2Cl_2)/M06-2X/def2-SVP results for compound **4b**; threshold for printing excitations was chosen to be $f \geq 0.10$.

state no.	λ / nm	f	Assignment
**2**	364.3	0.2607	H−1 → L (c = 0.8622)
**35**	227.6	0.1561	H−6 → L+2 (c = 0.1462)
**36**	227.7	0.1348	H−2 → L+5 (c = 0.1518)
**38**	231.4	0.2272	H−2 → L+3 (c = 0.3917)
**45**	220.6	0.1342	H−2 → L+7 (c = 0.1440)
**47**	219.4	0.1033	H−2 → L+5 (c = 0.2788)

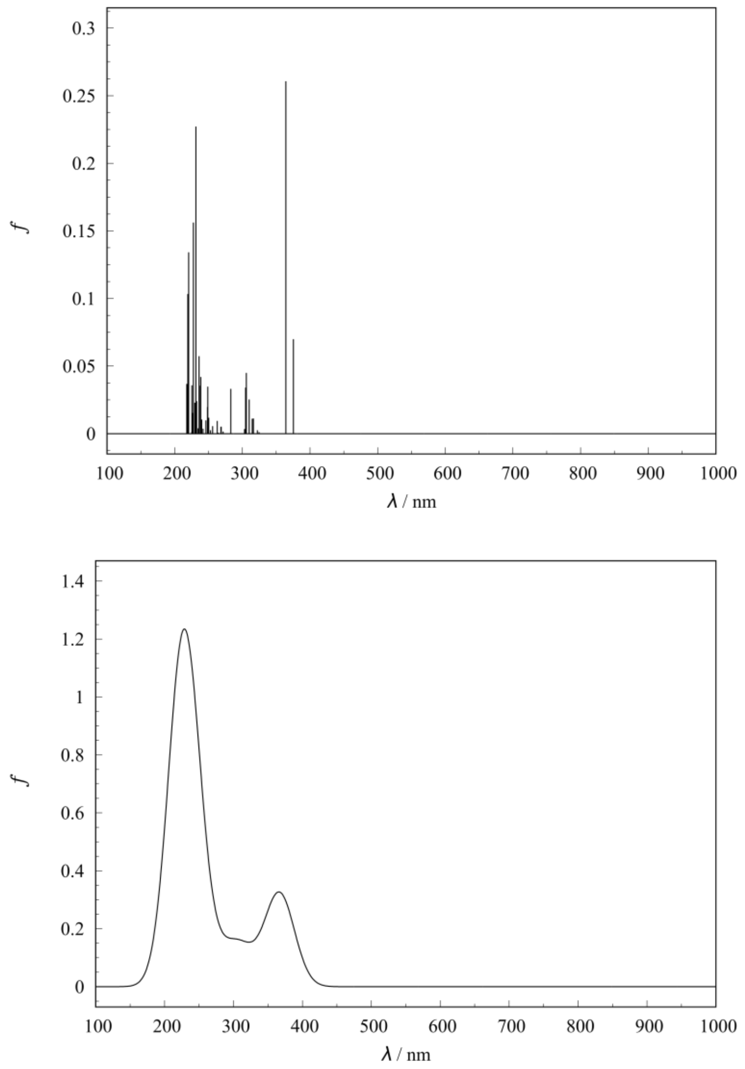

Fig. S53 UV-visible spectrum (lines: top; Gaussian line broadening with a full width at half maximum (fwhm) of 50 cm^{-1}: bottom) of **4b** calculated at TD-PCM(CH_2Cl_2)/M06-2X/def2-SVP.

Table S11. Wavelength (λ), oscillator strength (f) and main assignment of the TD-PCM(CH_2Cl_2)/M06-2X/def2-SVP results for compound **4c**; threshold for printing excitations was chosen to be $f \geq 0.10$.

**state no.**	**λ / nm**	***f***	**Assignment**
**2**	361.5	0.2992	H−1 → L (c = 0.8667)
**38**	233.3	0.2111	H−2 → L+3 (c = 0.3440)
**45**	220.7	0.2299	H−6 → L+1 (c = 0.1828)
**47**	219.7	0.1075	H−2→ L+4 (c = 0.1957)

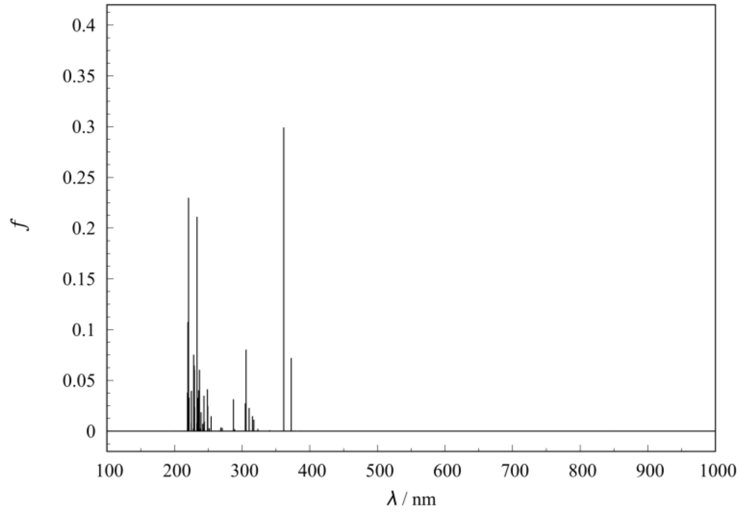

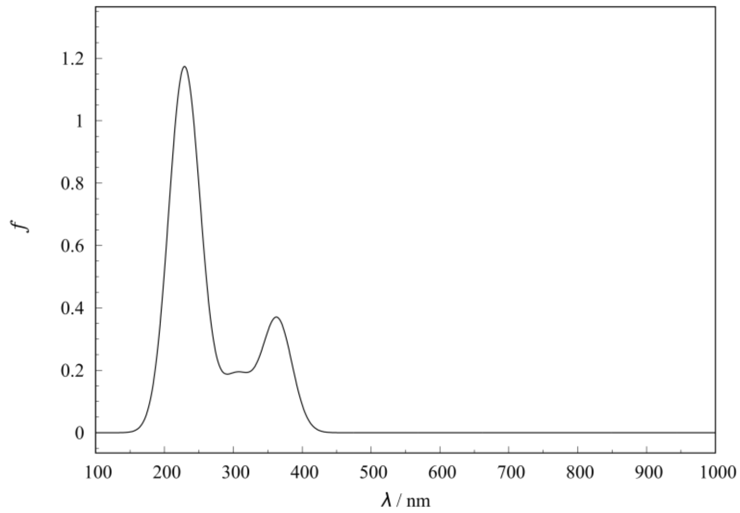

Fig. S54 UV-visible spectrum (lines: top; Gaussian line broadening with a full width at half maximum (fwhm) of 50 cm^{-1}: bottom) of **4c** calculated at TD-PCM(CH_2Cl_2)/M06-2X/def2-SVP.

Table S12. Wavelength (*λ*), oscillator strength (*f*) and main assignment of the TD-PCM(CH_2Cl_2)/M06-2X/def2-SVP results for compound **4d**; threshold for printing excitations was chosen to be $f \geq 0.10$.

state no.	*λ* / nm	*f*	Assignment
**2**	347.5	0.6039	H−1 → L (*c* = 0.8967)
**5**	302.4	0.1161	H−1 → L+1 (*c* = 0.5010)
**43**	229.0	0.2769	H−3 → L+3 (*c* = 0.5477)
**50**	218.9	0.1498	H → L+8 (*c* = 0.5420)

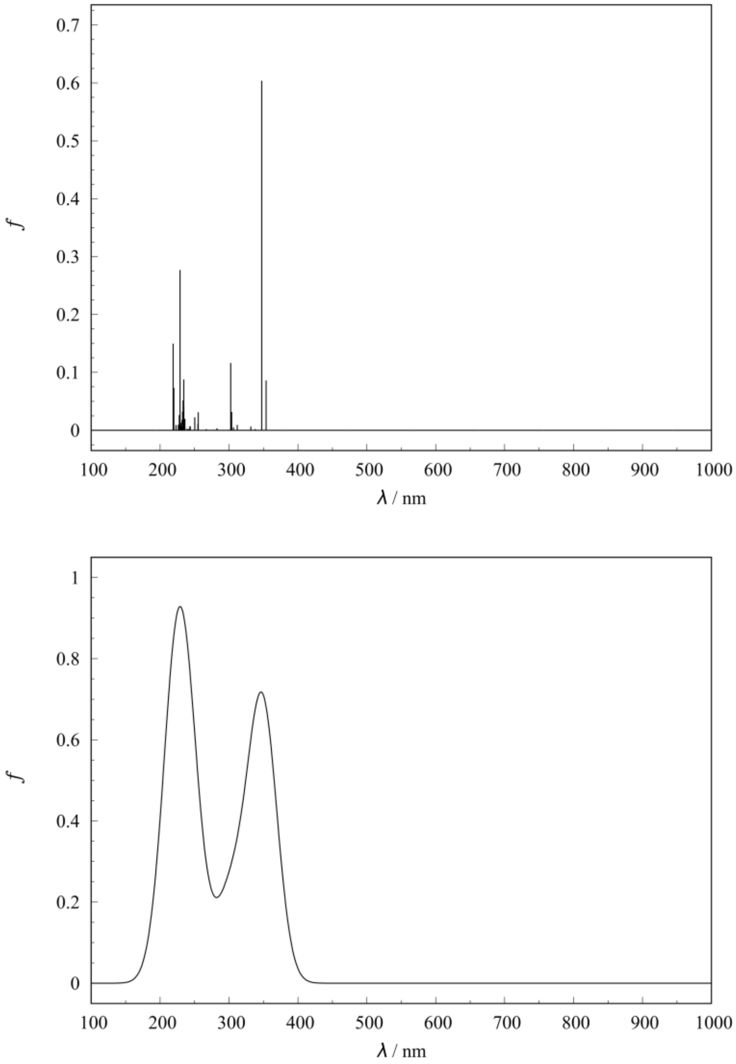

Fig. S55 UV-visible spectrum (lines: top; Gaussian line broadening with a full width at half maximum (fwhm) of 50 cm^{-1}: bottom) of **4d** calculated at TD-PCM(CH_2Cl_2)/M06-2X/def2-SVP.

Table S13. Wavelength (λ), oscillator strength (f) and main assignment of the TD-PCM(CH_2Cl_2)/M06-2X/def2-SVP results for compound **5a**; threshold for printing excitations was chosen to be $f \geq 0.07$.

**state no.**	**λ / nm**	**f**	**Assignment**
**6**	429.3	0.1456	H → L (c = 0.4725)
**9**	367.7	0.0760	H−2 → L+8 (c = 0.4109)
**10**	355.1	0.0701	H−1 → L (c = 0.9218)
**13**	313.2	0.2923	H−3 → L (c = 0.5462)
**39**	240.0	0.0754	H−1 → L+2 (c = 0.2765)

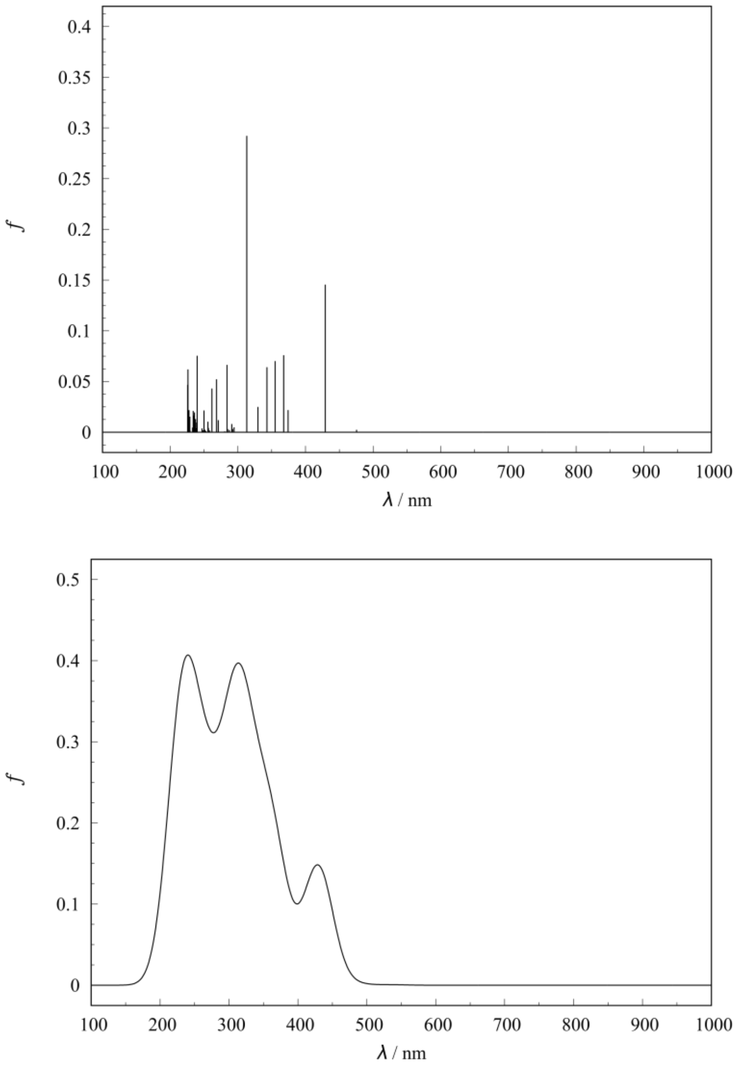

Fig. S56 UV-visible spectrum (lines: top; Gaussian line broadening with a full width at half maximum (fwhm) of 50 cm^{-1}: bottom) of **5a** calculated at TD-PCM(CH_2Cl_2)/M06-2X/def2-SVP.

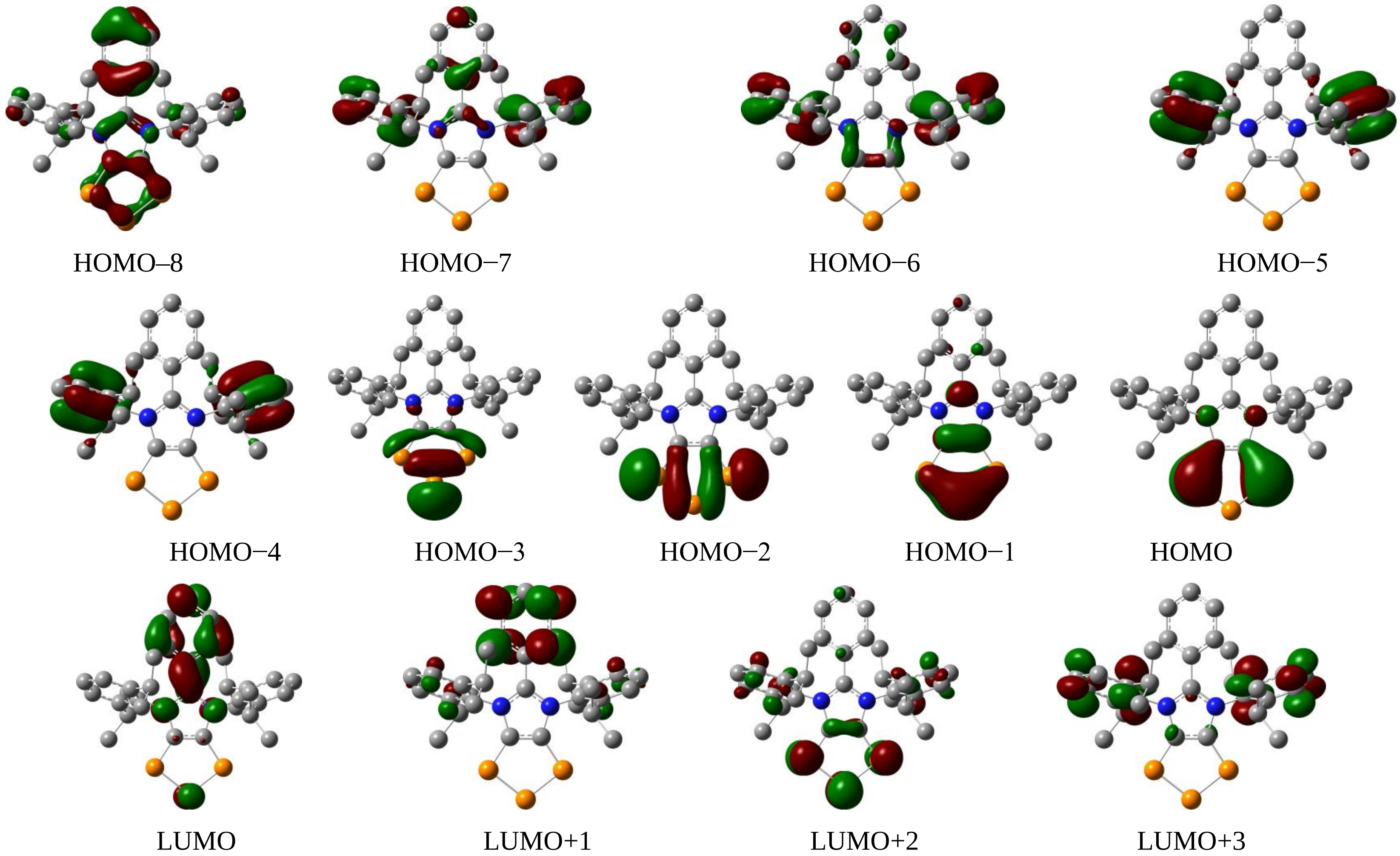

Fig. S57 Selected molecular orbitals (from HOMO−8 to LUMO+3) of compound **4a**, calculated at M06-2X/def2-TZVPP//def2-SVP. The isovalue was arbitrarily chosen to be 0.04. Hydrogen atoms were omitted for clarity reasons.

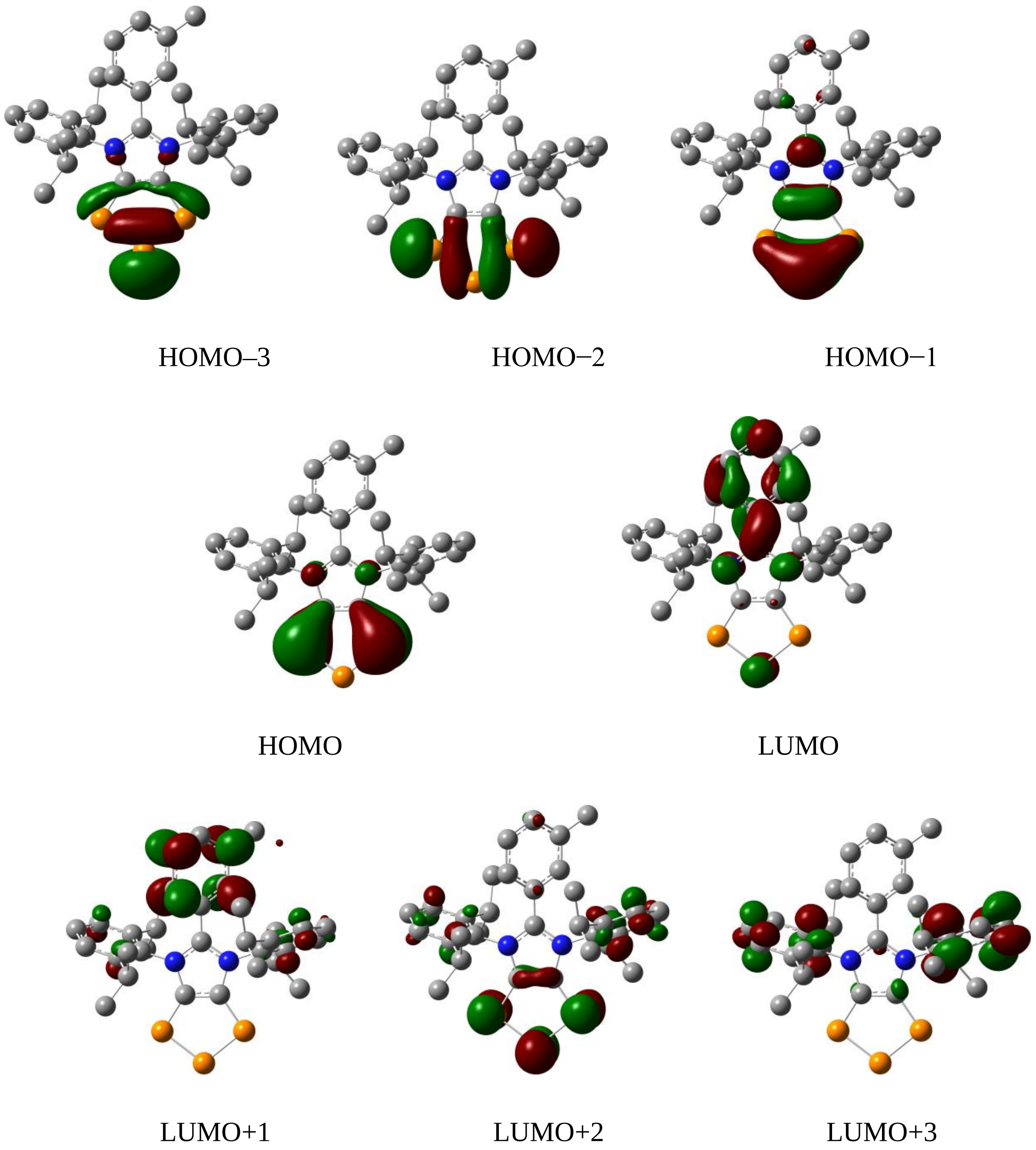

Fig. S58 Selected molecular orbitals (from HOMO−3 to LUMO+3) of compound **4b**, calculated at M06-2X/def2-TZVPP//def2-SVP. The isovalue was arbitrarily chosen to be 0.04. Hydrogen atoms were omitted for clarity reasons.

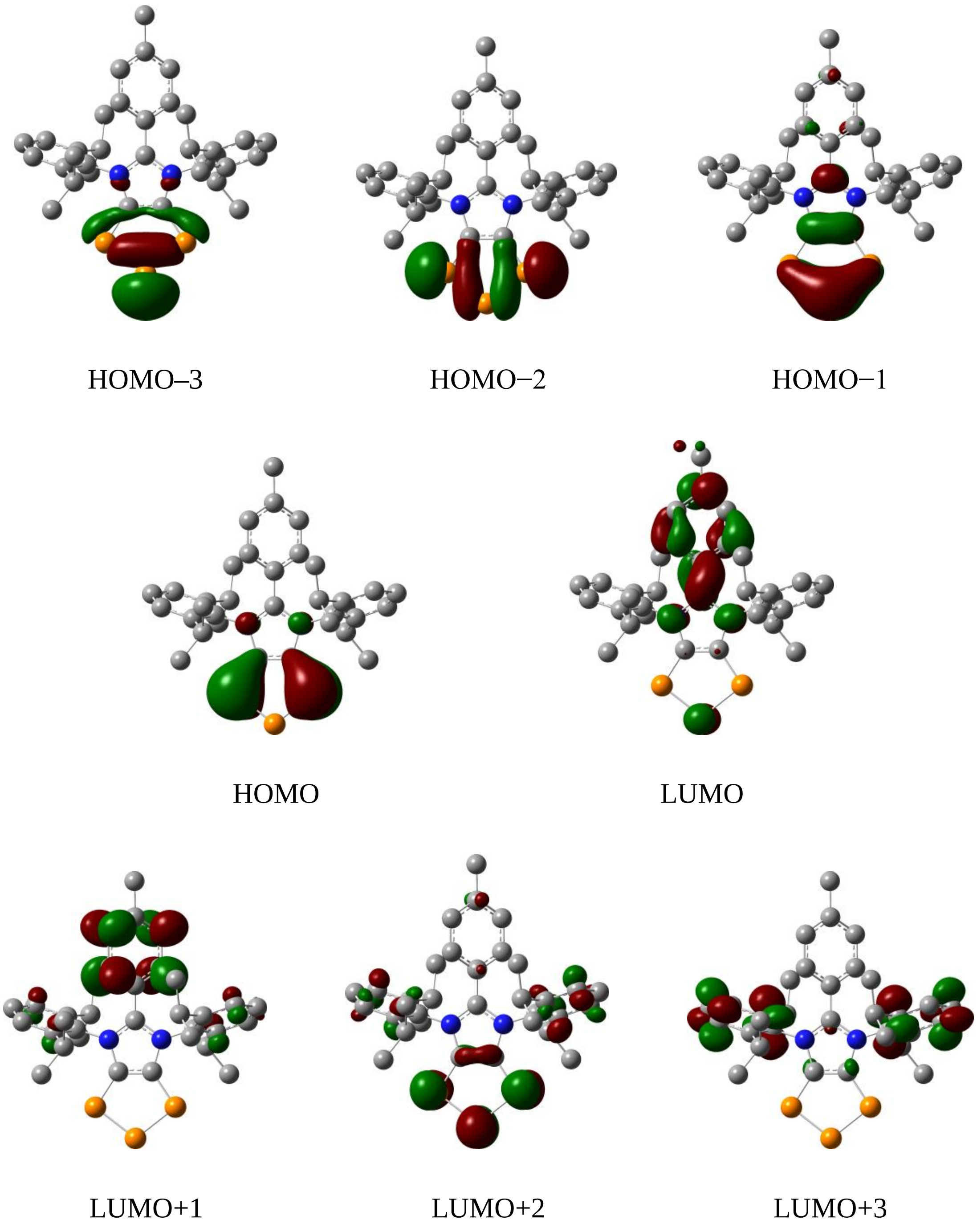

Fig. S59 Selected molecular orbitals (from HOMO−3 to LUMO+3) of compound **4c**, calculated at M06-2X/def2-TZVPP//def2-SVP. The isovalue was arbitrarily chosen to be 0.04. Hydrogen atoms were omitted for clarity reasons.

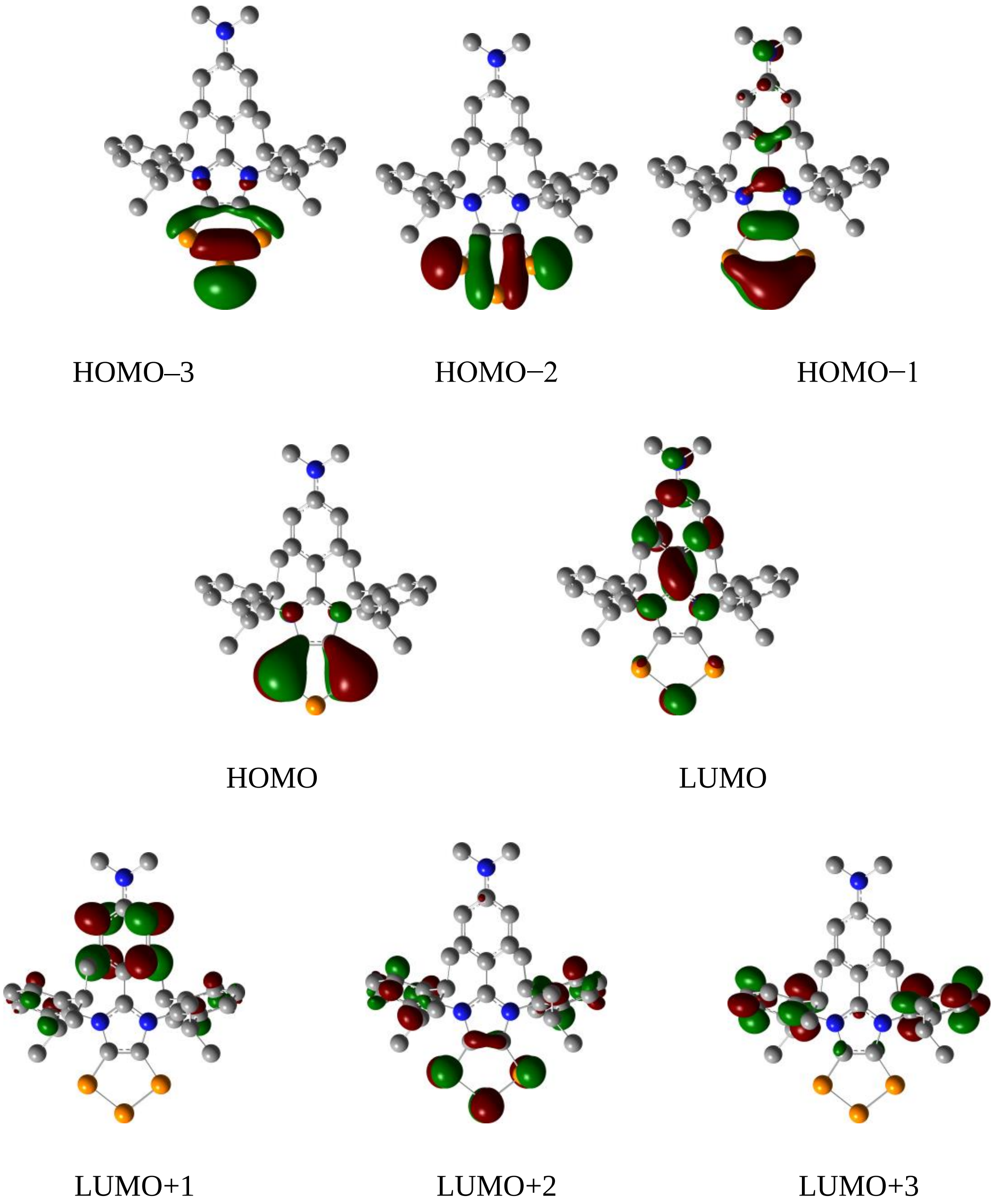

Fig. S60 Selected molecular orbitals (from HOMO−3 to LUMO+3) of compound **4d**, calculated at M06-2X/def2-TZVPP//def2-SVP. The isovalue was arbitrarily chosen to be 0.04. Hydrogen atoms were omitted for clarity reasons.

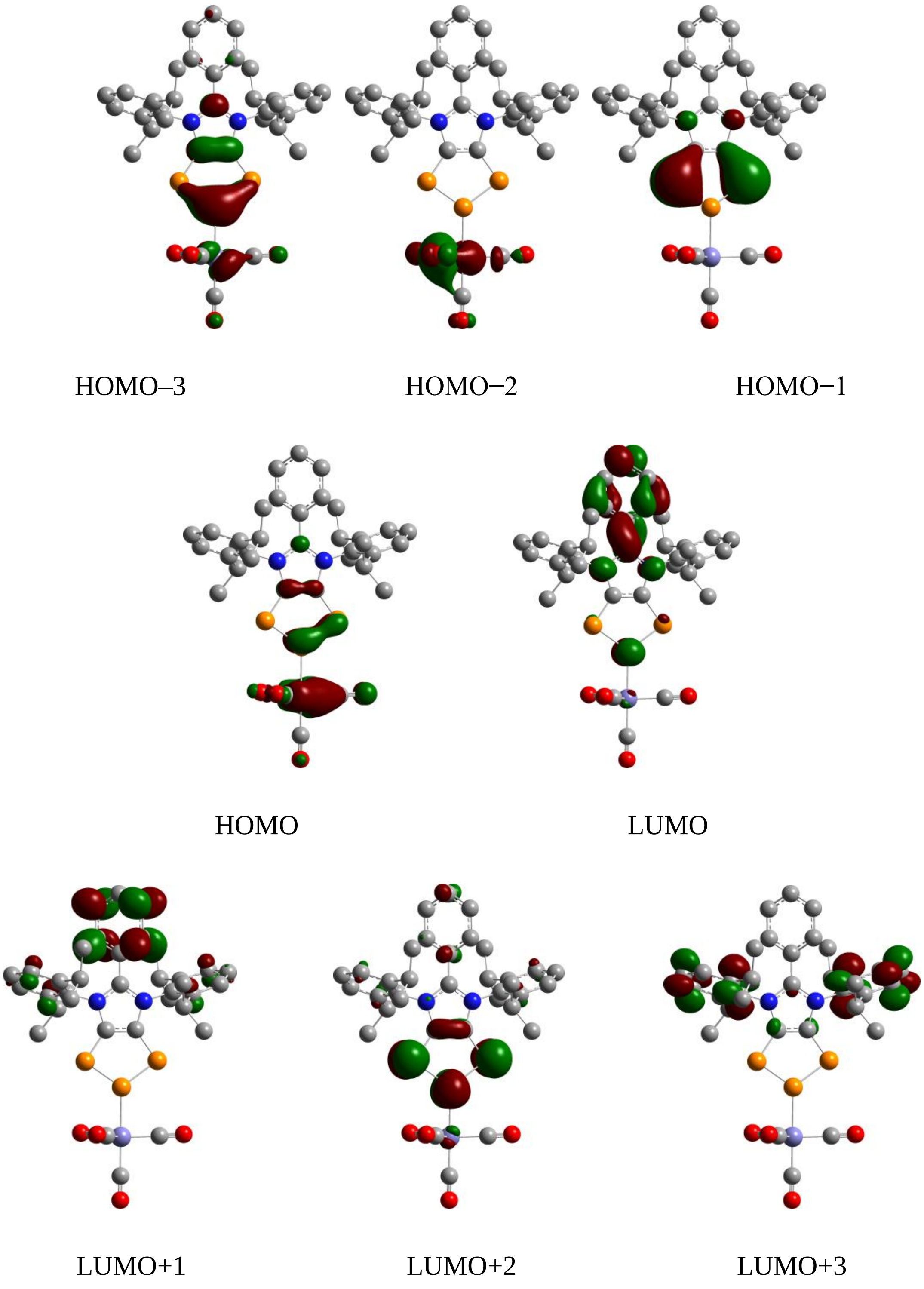

Fig. S61 Selected molecular orbitals (from HOMO−3 to LUMO+3) of compound **5a**, calculated at M06-2X/def2-TZVPP//def2-SVP. The isovalue was arbitrarily chosen to be 0.04. Hydrogen atoms were omitted for clarity reasons.

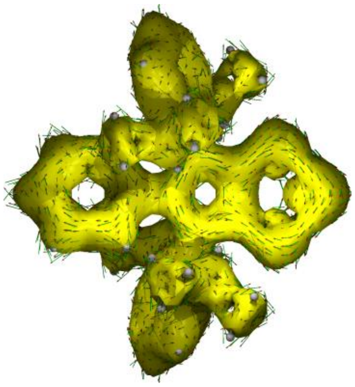

4a

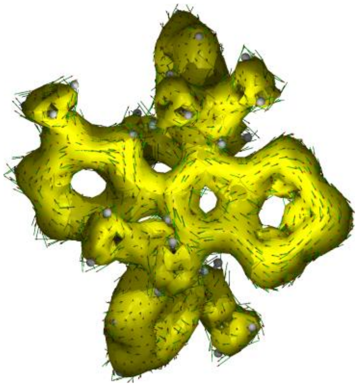

4b

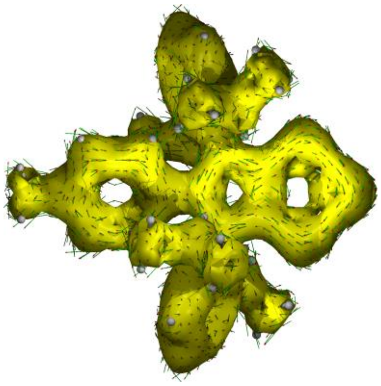

4c

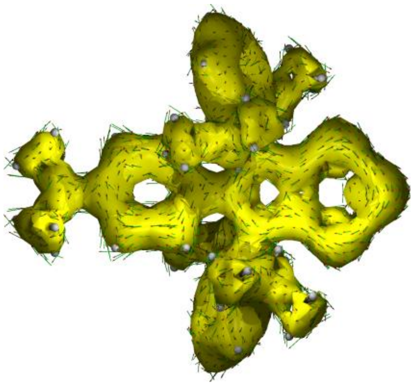

4d

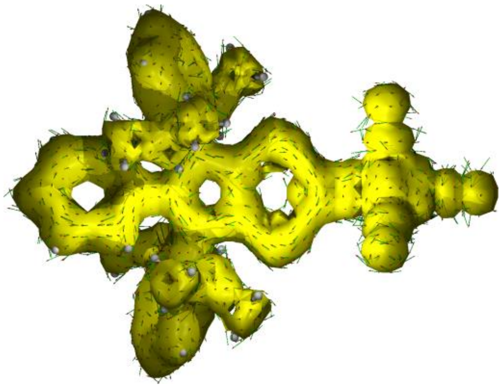

5a

Fig. S62 AICD plots (based on M06-2X/def2-TZVPP//def2-SVP calculations) of compounds **4a**–**4d** and **5a**. The isovalue was arbitrarily chosen to be 0.03, the magnetic field is orthogonal to the C_2P_3 plane and points towards the viewer, thus clockwise ring currents represent aromatic systems whereas counterclockwise ring currents are indicative for antiaromatic systems.

Table S14. Cartesian Coordinates of compound **4a** (in Å), calculated at M06-2X/def2-SVP level of theory, including positions of the NICS dummy atoms (Bq). (E = −2412.6322178, ν_{min} = 16.5 cm^{-1})

P	-1.651916	-3.191784	0.206029
P	-0.000007	-4.491167	-0.000019
C	-0.697239	-1.714832	0.072855
P	1.651905	-3.191787	-0.206067
N	-1.084730	-0.370667	0.081113
C	0.697231	-1.714834	-0.072878
C	-0.000000	0.423194	0.000002
C	-2.440257	0.061126	0.269769
N	1.084727	-0.370670	-0.081119
C	0.000004	1.895690	0.000010
C	-3.342828	-0.075087	-0.800146
C	-2.804125	0.595707	1.515866
C	2.440256	0.061123	-0.269768
C	-1.034264	2.609033	-0.626509
C	1.034277	2.609020	0.626533
C	-4.646705	0.385533	-0.597273
C	-2.949828	-0.663442	-2.148350
C	-4.120675	1.042778	1.663462
C	-1.843137	0.675674	2.690513
C	3.342824	-0.075107	0.800147
C	2.804127	0.595722	-1.515857
H	-1.844817	2.078540	-1.122041
C	-1.030893	4.000897	-0.624391
H	1.844827	2.078518	1.122060
C	1.030916	4.000884	0.624427
H	-5.373057	0.302959	-1.408020
C	-5.031814	0.945740	0.617347
H	-1.932266	-1.070307	-2.064720
C	-3.871053	-1.825901	-2.532078
C	-2.941937	0.406990	-3.246738
H	-4.436865	1.466428	2.618706
H	-0.852298	0.333740	2.357169
C	-2.289907	-0.276050	3.805929
C	-1.691883	2.113238	3.198095
C	4.646702	0.385514	0.597283
C	2.949821	-0.663482	2.148341
C	4.120678	1.042793	-1.663444
C	1.843143	0.675704	-2.690506
H	-1.840546	4.538539	-1.118861
C	0.000014	4.702037	0.000021
H	1.840574	4.538517	1.118901
H	-6.054610	1.300599	0.752211
H	-3.887488	-2.587966	-1.741157
H	-3.512746	-2.298969	-3.457581
H	-4.900226	-1.478160	-2.710383
H	-3.933037	0.874915	-3.350809
H	-2.681124	-0.047513	-4.213626
H	-2.209364	1.202393	-3.045006
H	-3.275688	0.014939	4.199818
H	-1.571705	-0.253717	4.638635
H	-2.359768	-1.307727	3.433234
H	-1.387182	2.795201	2.389892
H	-0.933322	2.158027	3.993535
H	-2.637516	2.485972	3.620387
H	5.373053	0.302927	1.408029
C	5.031814	0.945738	-0.617328
H	1.932259	-1.070346	2.064702
C	3.871045	-1.825945	2.532055
C	2.941925	0.406935	3.246743
H	4.436871	1.466457	-2.618681
H	0.852302	0.333767	-2.357171
C	2.289917	-0.276010	-3.805930
C	1.691892	2.113273	-3.198074
H	0.000018	5.792852	0.000025
H	6.054612	1.300598	-0.752184
H	3.887482	-2.587999	1.741123
H	3.512734	-2.299027	3.457550
H	4.900217	-1.478207	2.710369

H	3.933023	0.874861	3.350825
H	2.681108	-0.047580	4.213625
H	2.209351	1.202340	3.045019
H	3.275702	0.014980	-4.199809
H	1.571721	-0.253665	-4.638641
H	2.359772	-1.307691	-3.433246
H	1.387187	2.795227	-2.389864
H	0.933334	2.158070	-3.993516
H	2.637526	2.486011	-3.620358
Bq	0.000000	-2.861000	0.000000
Bq	0.121000	-2.861000	0.993000
Bq	0.242000	-2.861000	1.986000
Bq	0.000000	-0.750000	0.000000
Bq	0.083000	-0.750000	0.997000
Bq	0.166000	-0.750000	1.994000

Table S15. Cartesian Coordinates of compound **4b** (in Å), calculated at M06-2X/def2-SVP level of theory, including positions of the NICS dummy atoms (Bq). ($E = -2451.8998374$, $\nu_{min} = 13.5\ cm^{-1}$)

P	1.068075	-3.532852	-0.095343
P	-0.749770	-4.539488	-0.471927
C	0.347630	-1.923149	-0.083051
P	-2.183190	-2.991414	-0.563183
N	0.929230	-0.661072	0.080510
C	-1.025653	-1.697622	-0.252759
C	-0.023425	0.291156	0.067987
C	2.326808	-0.459208	0.337226
N	-1.209646	-0.317025	-0.119400
C	0.195907	1.738440	0.226297
C	2.724580	-0.112448	1.638200
C	3.233831	-0.611987	-0.726817
C	-2.478978	0.333750	-0.275883
C	1.347388	2.336399	-0.302554
C	-0.735133	2.536949	0.909585
C	4.086193	0.121632	1.853420
C	1.748361	-0.005414	2.798020
C	4.583230	-0.369011	-0.455560
C	2.803395	-0.987974	-2.138039
C	-2.720440	1.055332	-1.455774
C	-3.423282	0.217873	0.759771
H	2.076473	1.731372	-0.839023
C	1.591360	3.705009	-0.161144
C	-0.499494	3.899670	1.054525
H	-1.636320	2.098332	1.332799
H	4.429234	0.395566	2.853053
C	5.006675	0.001281	0.817780
H	0.730242	-0.169325	2.415575
C	1.786325	1.386309	3.437924
C	2.020873	-1.109792	3.825338
H	5.314988	-0.471463	-1.259222
H	1.733267	-1.238054	-2.118354
C	3.549887	-2.232851	-2.628267
C	2.998707	0.179576	-3.113627
C	-3.949875	1.711729	-1.567181
C	-1.721746	1.123096	-2.599417
C	-4.636608	0.891829	0.595122
C	-3.161839	-0.568034	2.037381
C	2.832278	4.311399	-0.761253
C	0.653462	4.480635	0.526888
H	-1.222371	4.514248	1.592134
H	6.065019	0.187697	1.006116
H	2.761493	1.577763	3.911214
H	1.016911	1.466370	4.220097
H	1.606782	2.176757	2.693426
H	1.955425	-2.102205	3.357527
H	1.287290	-1.060630	4.643337
H	3.024978	-1.001287	4.263071
H	3.419112	-3.067295	-1.926053
H	4.626245	-2.033094	-2.744583
H	3.159228	-2.543694	-3.607819
H	2.391302	1.055152	-2.840281
H	2.704865	-0.124959	-4.128704
H	4.053284	0.493969	-3.148596
H	-4.169588	2.284482	-2.470315
C	-4.896546	1.637332	-0.551180
H	-0.806064	0.594144	-2.296992
C	-2.272836	0.391838	-3.828640
C	-1.335965	2.569619	-2.925819
H	-5.390849	0.831010	1.381948
H	-2.218123	-1.118497	1.918105
C	-3.015170	0.363667	3.246818
C	-4.261351	-1.606239	2.282373
H	-5.849847	2.157229	-0.656848
H	-1.533334	0.400411	-4.642771
H	-3.189206	0.877451	-4.197423
H	-2.511845	-0.653135	-3.585775
H	-0.955087	3.094621	-2.036672

H	-2.199773	3.132828	-3.310757
H	-0.555645	2.588956	-3.701170
H	-2.163010	1.050771	3.138298
H	-2.852255	-0.227066	4.160079
H	-3.923812	0.967772	3.393593
H	-5.229034	-1.123703	2.488940
H	-4.004441	-2.226322	3.153138
H	-4.374598	-2.266957	1.412151
H	0.829024	5.551143	0.651913
H	3.021411	5.314612	-0.358558
H	2.732149	4.399803	-1.853809
H	3.711378	3.683487	-0.559909
Bq	-0.509000	-2.937000	-0.293000
Bq	-0.662000	-3.024000	0.691000
Bq	-0.815000	-3.111000	1.675000
Bq	-0.196000	-0.908000	-0.061000
Bq	-0.312000	-1.002000	0.928000
Bq	-0.428000	-1.096000	1.917000

Table S16. Cartesian Coordinates of compound **4c** (in Å), calculated at M06-2X/def2-SVP level of theory, including positions of the NICS dummy atoms (Bq). (E = −2451.8998366, ν_{min} = 16.5 cm^{-1})

P	1.662266	-3.359298	0.204715
P	0.014098	-4.663944	0.001533
C	0.703217	-1.885173	0.072874
P	-1.641541	-3.368942	-0.202640
N	1.086862	-0.539366	0.081222
C	-0.690904	-1.889276	-0.071906
C	0.000004	0.251727	-0.000514
C	2.440951	-0.103694	0.269102
N	-1.082272	-0.545736	-0.081374
C	-0.004481	1.722924	-0.001655
C	2.804198	0.433197	1.514414
C	3.343414	-0.238184	-0.801070
C	-2.438798	-0.117761	-0.269123
C	1.027743	2.443986	-0.618478
C	-1.043191	2.439297	0.616303
C	4.119604	0.883936	1.660916
C	1.842940	0.514018	2.688766
C	4.646253	0.225940	-0.599414
C	2.950141	-0.827011	-2.148937
C	-2.805405	0.416414	-1.514600
C	-3.340095	-0.256393	0.801548
H	1.846925	1.922111	-1.109124
C	1.017389	3.835906	-0.615866
C	-1.041252	3.828161	0.612477
H	-1.858005	1.911597	1.108026
H	4.435029	1.309519	2.615587
C	5.030565	0.788226	0.614499
H	0.854689	0.162681	2.357637
C	1.681549	1.954152	3.186186
C	2.296426	-0.426302	3.811050
H	5.372308	0.144629	-1.410553
H	1.935596	-1.240602	-2.062587
C	3.877158	-1.982926	-2.538017
C	2.930988	0.245979	-3.244661
C	-4.123207	0.860305	-1.660719
C	-1.845389	0.500603	-2.689738
C	-4.645368	0.200932	0.600269
C	-2.943172	-0.842476	2.149545
H	1.831094	4.373603	-1.106025
C	-0.013260	4.554626	-0.003082
H	-1.857667	4.361857	1.103558
H	6.052533	1.145812	0.748545
H	2.625466	2.336953	3.603416
H	0.924498	1.998715	3.983135
H	1.369451	2.627495	2.373532
H	2.374463	-1.459996	3.445709
H	1.577514	-0.403717	4.643148
H	3.279623	-0.125056	4.203711
H	3.900316	-2.746520	-1.748697
H	4.903787	-1.629001	-2.718859
H	3.518753	-2.456220	-3.463376
H	2.193688	1.035632	-3.037524
H	2.669587	-0.207736	-4.211790
H	3.918502	0.720996	-3.351057
H	-4.441336	1.283518	-2.615545
C	-5.033129	0.760581	-0.613787
H	-0.854780	0.156122	-2.358506
C	-2.293948	-0.445157	-3.809447
C	-1.693120	1.940496	-3.190606
H	-5.370623	0.116338	1.411791
H	-1.926353	-1.250437	2.063108
C	-2.929793	0.230959	3.244922
C	-3.863649	-2.003414	2.539215
C	-0.031418	6.058834	-0.000119
H	-6.056992	1.112826	-0.747534
H	-1.575864	-0.420071	-4.642185
H	-3.279340	-0.150952	-4.201955
H	-2.365252	-1.478446	-3.441574
H	-1.384662	2.617580	-2.379681

H	-2.639516	2.316560	-3.608356
H	-0.936689	1.987901	-3.987956
H	-2.197127	1.024789	3.037306
H	-2.665443	-0.220966	4.212088
H	-3.919995	0.700297	3.351549
H	-4.892189	-1.655138	2.720170
H	-3.502389	-2.474410	3.464635
H	-3.882714	-2.767367	1.750136
H	0.859649	6.470767	-0.490010
H	-0.069736	6.447359	1.027863
H	-0.919022	6.438863	-0.526920
Bq	0.009000	-3.033000	0.001000
Bq	-0.111000	-3.033000	0.994000
Bq	-0.231000	-3.033000	1.987000
Bq	0.003000	-0.922000	0.000000
Bq	-0.080000	-0.922000	0.997000
Bq	-0.163000	-0.922000	1.994000

Table S17. Cartesian Coordinates of compound **4d** (in Å), calculated at M06-2X/def2-SVP level of theory, including positions of the NICS dummy atoms (Bq). (E = −2546.4439887, ν_{min} = 11.7 cm^{-1})

P	-3.701488	-1.649741	-0.210689
P	-5.003303	0.000040	0.000006
C	-2.224826	-0.695668	-0.076772
P	-3.701460	1.649801	0.210692
N	-0.878218	-1.083355	-0.091807
C	-2.224814	0.695703	0.076773
C	-0.081493	-0.000001	-0.000003
C	-0.448124	-2.435023	-0.299538
N	-0.878200	1.083367	0.091804
C	1.384404	-0.000012	-0.000006
C	-0.580757	-3.351833	0.758505
C	0.082757	-2.783934	-1.551585
C	-0.448082	2.435027	0.299538
C	2.115197	-1.051632	0.578309
C	2.115210	1.051598	-0.578323
C	-0.119945	-4.652949	0.538762
C	-1.162134	-2.972052	2.113328
C	0.531371	-4.097868	-1.716708
C	0.159961	-1.807190	-2.713207
C	-0.580704	3.351843	-0.758501
C	0.082809	2.783926	1.551585
H	1.597406	-1.888704	1.042901
C	3.500239	-1.057468	0.583119
H	1.597429	1.888678	-1.042911
C	3.500252	1.057414	-0.583138
H	-0.200271	-5.389366	1.340646
C	0.438065	-5.022285	-0.681849
H	-1.583845	-1.960251	2.036008
C	-2.308639	-3.908700	2.506076
C	-0.079230	-2.950108	3.199150
H	0.952736	-4.401401	-2.677174
H	-0.199107	-0.826297	-2.369261
C	-0.773969	-2.252352	-3.844022
C	1.600322	-1.629054	-3.204202
C	-0.119872	4.652951	-0.538755
C	-1.162093	2.972076	-2.113322
C	0.531444	4.097852	1.716711
C	0.160001	1.807177	2.713204
H	4.010787	-1.896426	1.050879
C	4.239441	-0.000034	-0.000014
H	4.010810	1.896367	-1.050897
H	0.793371	-6.043095	-0.830455
H	-3.078966	-3.929576	1.723145
H	-2.775865	-3.559594	3.438091
H	-1.948148	-4.934888	2.676407
H	0.404724	-3.934464	3.295426
H	-0.525496	-2.697228	4.172086
H	0.701916	-2.205120	2.986989
H	-0.464575	-3.227602	-4.250109
H	-0.755001	-1.521609	-4.665898
H	-1.807896	-2.343078	-3.482317
H	2.267615	-1.317356	-2.386433
H	1.640722	-0.864908	-3.994703
H	1.992313	-2.566394	-3.628092
H	-0.200189	5.389372	-1.340636
C	0.438148	5.022275	0.681855
H	-1.583817	1.960280	-2.036003
C	-2.308587	3.908741	-2.506062
C	-0.079194	2.950119	-3.199149
H	0.952817	4.401375	2.677176
H	-0.199088	0.826291	2.369258
C	-0.773914	2.252354	3.844026
C	1.600361	1.629012	3.204191
N	5.606601	-0.000045	-0.000020
H	0.793471	6.043079	0.830464
H	-3.078910	3.929625	-1.723127
H	-2.775822	3.559645	-3.438076
H	-1.948081	4.934925	-2.676391
H	0.404773	3.934469	-3.295424

H	-0.525467	2.697248	-4.172084
H	0.701943	2.205121	-2.986993
H	-0.464498	3.227598	4.250113
H	-0.754954	1.521609	4.665900
H	-1.807841	2.343101	3.482328
H	2.267644	1.317303	2.386417
H	1.640751	0.864863	3.994690
H	1.992372	2.566344	3.628080
C	6.325460	-1.099801	0.602097
C	6.325473	1.099730	-0.602085
H	6.087035	2.059371	-0.113809
H	6.095918	1.199265	-1.676230
H	7.401818	0.925487	-0.500117
H	6.086993	-2.059461	0.113875
H	6.095920	-1.199276	1.676252
H	7.401806	-0.925588	0.500099
Bq	-3.371000	0.000000	0.000000
Bq	-3.371000	-0.124000	0.992000
Bq	-3.371000	-0.248000	1.984000
Bq	-1.258000	0.000000	0.000000
Bq	-1.258000	-0.092000	0.996000
Bq	-1.258000	-0.184000	1.992000

Table S18. Cartesian Coordinates of compound **5a** (in Å), calculated at M06-2X/def2-SVP level of theory, including positions of the NICS dummy atoms (Bq). ($E = -4128.9310008$, $\nu_{min} = 8.2$ cm^{-1})

Fe	5.210851	0.023213	0.000164
P	2.868672	0.051938	0.018752
C	5.162857	1.823141	-0.261258
C	5.156870	-0.650681	1.691813
C	5.113721	-1.120629	-1.415138
C	7.051136	0.004609	-0.025097
P	1.614301	1.717857	-0.197454
P	1.655976	-1.643086	0.228552
O	5.085423	2.951447	-0.422150
O	5.096439	-1.075265	2.749765
O	5.023344	-1.840716	-2.296683
O	8.187105	-0.004304	-0.041870
C	0.164283	0.714377	-0.061220
C	0.180824	-0.678384	0.086747
N	-1.181566	1.085498	-0.076208
N	-1.155023	-1.083070	0.091270
C	-1.962502	-0.008895	0.004172
C	-1.627804	2.437694	-0.266671
C	-1.567397	-2.447655	0.268779
C	-3.434414	-0.027604	-0.002668
C	-2.163343	2.794117	-1.514683
C	-1.502063	3.341463	0.803763
C	-1.408338	-3.339698	-0.807245
C	-2.104855	-2.826917	1.509051
C	-4.130923	-1.071271	-0.632426
C	-4.162725	0.998205	0.620309
C	-2.619999	4.107056	-1.664465
C	-2.238577	1.830645	-2.687757
C	-1.972023	4.641472	0.597383
C	-0.916755	2.955473	2.155645
C	-1.845987	-4.652973	-0.614507
C	-0.822032	-2.928189	-2.151243
C	-2.528368	-4.152439	1.645141
C	-2.216303	-1.873940	2.687782
C	-5.522657	-1.084930	-0.637206
C	-5.554352	0.977077	0.611854
C	-2.531653	5.020298	-0.619414
C	-1.303471	2.288348	-3.812798
C	-3.678325	1.660297	-3.183713
C	0.227685	3.894228	2.551665
C	-1.995218	2.928943	3.246046
C	-2.406565	-5.055240	0.594371
C	0.350724	-3.832515	-2.544598
C	-1.891771	-2.922211	-3.250567
C	-1.281494	-2.315828	3.819374
C	-3.664879	-1.743539	3.169926
C	-6.239219	-0.062676	-0.016033
H	-3.588284	-1.875397	-1.125262
H	-3.644925	1.815591	1.118047
H	-6.104675	1.779684	1.103744
H	-7.329908	-0.076252	-0.021226
H	-2.775898	2.182149	3.039269
H	-2.481069	3.911737	3.345453
H	-1.543105	2.677132	4.216310
H	0.994957	3.934021	1.766612
H	0.702140	3.538022	3.477088
H	-0.139213	4.915458	2.735933
H	-0.494779	1.943671	2.075254
H	-1.897320	5.369738	1.407027
H	-2.893282	6.040312	-0.756791
H	-3.044288	4.418832	-2.620799
H	-1.881276	0.844426	-2.356211
H	-4.065660	2.600944	-3.603789
H	-4.350711	1.347769	-2.370557
H	-3.719251	0.900856	-3.978438
H	-1.612549	3.268355	-4.206928
H	-1.323242	1.567712	-4.643303
H	-0.268925	2.375200	-3.451954
H	-4.336589	-1.441964	2.352108

H	-3.733047	-0.991348	3.969638
H	-4.032620	-2.696808	3.579031
H	-1.880704	-0.876782	2.366405
H	-1.569828	-3.305975	4.203759
H	-1.327901	-1.601768	4.654461
H	-0.241394	-2.374145	3.468963
H	-2.953160	-4.482318	2.595159
H	-2.742771	-6.085304	0.720978
H	-1.745034	-5.372959	-1.428706
H	-0.428199	-1.906030	-2.060025
H	0.013074	-4.861602	-2.740800
H	1.112127	-3.858088	-1.753324
H	0.824477	-3.455607	-3.462076
H	-2.349892	-3.916983	-3.361841
H	-1.438350	-2.650659	-4.214891
H	-2.694146	-2.198455	-3.044782
H	-6.048021	-1.901004	-1.134192
Bq	1.297000	0.033000	0.015000
Bq	1.293000	0.156000	1.007000
Bq	1.289000	0.289000	1.999000
Bq	-0.791000	0.006000	0.009000
Bq	-0.796000	0.091000	1.005000
Bq	-0.801000	0.176000	2.001000

Table S19. Cartesian Coordinates of compound **1a** (in Å), calculated at M06-2X/def2-SVP level of theory. (E = −1389.9096992, ν_{min} = 14.6 cm^{-1})

C	-0.676507	-0.067897	-2.292540
N	-1.078476	-0.101706	-0.973918
C	0.676466	0.068308	-2.292540
C	-0.000001	0.000012	-0.176634
C	-2.450341	-0.271195	-0.550836
N	1.078459	0.101877	-0.973919
C	0.000014	-0.000127	1.290572
C	-3.323014	0.820992	-0.700095
C	-2.839837	-1.511362	-0.021049
C	2.450330	0.271300	-0.550830
C	-0.966085	0.736321	1.990221
C	0.966132	-0.736706	1.990057
C	-4.652659	0.626424	-0.318441
C	-2.874869	2.168114	-1.247331
C	-4.180584	-1.643796	0.354088
C	-1.890939	-2.684769	0.159540
C	3.323006	-0.820857	-0.700296
C	2.839828	1.511375	-0.020830
H	-1.711878	1.318872	1.448758
C	-0.961673	0.733598	3.381833
H	1.711915	-1.319151	1.448466
C	0.961753	-0.734250	3.381670
H	-5.365969	1.445231	-0.424638
C	-5.078245	-0.592699	0.201614
H	-1.773898	2.175100	-1.293214
C	-3.407761	2.380548	-2.669587
C	-3.296363	3.325984	-0.336500
H	-4.526672	-2.591974	0.769211
H	-0.884457	-2.380060	-0.167039
C	-2.311601	-3.869027	-0.717805
C	-1.796001	-3.089028	1.635486
C	4.652657	-0.626348	-0.318628
C	2.874858	-2.167885	-1.247760
C	4.180580	1.643750	0.354310
C	1.890926	2.684745	0.159980
H	-1.709961	1.310667	3.925504
C	0.000048	-0.000393	4.077455
H	1.710055	-1.311421	3.925214
H	-6.120581	-0.722609	0.495128
H	-3.089057	1.580204	-3.352410
H	-3.055465	3.339496	-3.074791
H	-4.507786	2.395432	-2.669173
H	-4.389674	3.436042	-0.305982
H	-2.882445	4.271219	-0.714474
H	-2.942889	3.186878	0.695498
H	-3.304842	-4.241349	-0.427120
H	-1.598361	-4.698062	-0.607178
H	-2.354204	-3.587453	-1.779460
H	-1.525958	-2.233841	2.272566
H	-1.037766	-3.874386	1.767018
H	-2.755263	-3.490422	1.994389
H	5.365970	-1.445134	-0.424980
C	5.078244	0.592685	0.201635
H	1.773887	-2.174868	-1.293621
C	3.407722	-2.380065	-2.670065
C	3.296377	-3.325913	-0.337142
H	4.526669	2.591856	0.769596
H	0.884445	2.380095	-0.166656
C	2.311584	3.869171	-0.717140
C	1.795985	3.088724	1.636003
H	0.000061	-0.000496	5.168197
H	6.120584	0.722548	0.495156
H	3.088996	-1.579604	-3.352741
H	3.055424	-3.338944	-3.075430
H	4.507747	-2.394941	-2.669676
H	4.389690	-3.435970	-0.306667
H	2.882456	-4.271083	-0.715272
H	2.942925	-3.186989	0.694889
H	3.304823	4.241443	-0.426382

H	1.598340	4.698181	-0.606358
H	2.354191	3.587797	-1.778848
H	1.525938	2.233417	2.272920
H	1.037750	3.874058	1.767682
H	2.755247	3.490048	1.994985
H	-1.392234	-0.149523	-3.104349
H	1.392178	0.150084	-3.104347

References

1. O. V. Dolomanov, L. J. Bourhis, R. J. Gildea, J. A. K. Howard and H. Puschmann, *J. Appl. Cryst.* 2009 **42**, 339–341.

2. G. M. Sheldrick, *Acta Cryst. A* 2015, **71**, 3–8.

3. G. M. Sheldrick, *Acta Cryst. C* 2015, **71**, 3–8.

4. M. J. Frisch, G. W. Trucks, H. B. Schlegel, G. E. Scuseria, M. A. Robb, J. R. Cheeseman, G. Scalmani, V. Barone, B. Mennucci, G. A. Petersson, H. Nakatsuji, M. Caricato, X. Li, H. P. Hratchian, A. F. Izmaylov, J. Bloino, G. Zheng, J. L. Sonnenberg, M. Hada, M. Ehara, K. Toyota, R. Fukuda, J. Hasegawa, M. Ishida, T. Nakajima, Y. Honda, O. Kitao, H. Nakai, T. Vreven, J. J. A. Montgomery, J. E. Peralta, F. Ogliaro, M. Bearpark, J. J. Heyd, E. Brothers, K. N. Kudin, V. N. Staroverov, R. Kobayashi, J. Normand, K. Raghavachari, A. Rendell, J. C. Burant, S. S. Iyengar, J. Tomasi, M. Cossi, N. Rega, J. M. Millam, M. Klene, J. E. Knox, J. B. Cross, V. Bakken, C. Adamo, J. Jaramillo, R. Gomperts, R. E. Stratmann, O. Yazyev, A. J. Austin, R. Cammi, C. Pomelli, J. W. Ochterski, R. L. Martin, K. Morokuma, V. G. Zakrzewski, G. A. Voth, P. Salvador, J. J. Dannenberg, S. Dapprich, A. D. Daniels, O. Farkas, J. B. Foresman, J. V. Ortiz, J. Cioslowski and D. J. Fox, *Gaussian 16, Revision A.03*, Gaussian, Inc., Wallingford CT, 2016.

5. Y. Zhao and D. G. Truhlar, *Theor. Chem. Acc.* 2008, **120**, 215–241.

6. F. Weigend and R. Ahlrichs, *Phys. Chem. Chem. Phys.* 2005, **7**, 3297–3305.

7. J. W. McIver, and A. Komornic, *J. Am. Chem. Soc.* 1972, **94**, 2625–2633.

8. K. B. Wiberg, *Tetrahedron* 1968, **24**, 1083–1096.

9. (a) A. E. Reed and F. Weinhold, *J. Chem. Phys.* 1985, **83**, 1736–1740; (b) A. E. Reed, R. B. Weinstock and F. Weinhold, *J. Chem. Phys.* 1985, **83**, 735–746.

10. E. D. Glendening, A. E. Reed, J. E. Carpenter and F. Weinhold, NBO Version 3.1.

11. a) D. Geuenich, K. Hess, F. Koehler and R. Herges, *Chem. Rev.* 2005, **105**, 3758–3772; b) R. Herges and D. Geuenich, *J. Phys. Chem. A* 2001, **105**, 3214–3220.

12. F. Neese, *WIREs Comput. Mol. Sci.* 2017, **8**, e1327.

13 V. Barone and M. Cossi, *J. Phys. Chem. A* 1998, **102**, 1995–2001.
